# Macro/Microfracture evolution and instability behaviors of high-temperature granite under water-cooling subjected to Brazilian splitting test using the DIC technique

**DOI:** 10.1371/journal.pone.0294258

**Published:** 2023-11-29

**Authors:** Xinghui Wu, Xu Wu, Shukun Zhang, Yu Zhu

**Affiliations:** 1 School of City and Architecture Engineering, Zaozhuang University, Zaozhuang, Shandong, China; 2 Beijing Municipal Engineering Research Institute, Beijing, China; UNICAMP, University of Campinas, BRAZIL

## Abstract

To investigate the evolution and stability characteristics of granite thermal damage, a series of Brazilian splitting tests is conducted on high-temperature granite samples using digital image correlation (DIC) technology. The results show that the Brazilian tensile strength and P-wave velocity exhibit a clear decline beyond a temperature threshold of 450~600°C, with a linear relationship between them. The presence of micro-cracks alters the stress transfer path, disrupting the stress balance on the Brazilian disc and leading to complex fracture patterns. At temperatures below 450°C, high strain areas and the development of micro-cracks occur at both the upper and lower loading ends of the granite Brazilian disc. However, these phenomena are only observed at the upper loading end when the temperature exceeds 450°C. Thermal cracks also cause changes in the internal structure of rock samples, and temperature variations can affect both the P-wave velocity and tensile strength. In terms of the relationship between P-wave velocity and Brazilian tensile strength (BTS) of high-temperature granite under water cooling, the negative exponential function model proposed in this study fits the experimental data very well.

## 1. Introduction

Geothermal resources, broadly distributed and efficiently utilized, represent an increasingly valued renewable energy source with the potential for large-scale industrial development [1, 2]. Hot dry rock, a resource abundant in magmatic rocks like granite, specifically offers high thermal energy value [3]. Its exploitation through Enhanced Geothermal Systems (EGS) involves extracting heat from circulating water interacting with these high-temperature rocks [4]. In the process of heat transfer between high-temperature rock and circulating water, the physical and mechanical properties of high-temperature granite show a changing trend [5, 6]. Hot dry rock belongs to high temperature granite, there are also differences in the physical and mechanical properties with the conventional granite [7–9]. Chen et al. [10] conducted high temperature treatment of Beishan granite, explained the thermal rupture phenomenon of granite by using various monitoring methods, and quantified the degree of thermal damage of high temperature to granite.

In terms of the mechanical properties of rocks, the tensile performance is far less than the compressive performance, and the tensile performance of rocks is the key to judge the stability of deep geothermal engineering [11, 12]. It is quite common to determine the tensile properties of rock splitting properties indirectly by the Brazilian splitting test. For the study of the splitting characteristics of high temperature granite, Fang et al. [13] compared and analyzed the splitting and fracture pattern, load-displacement curve, and tensile strength of granite under the influence of 25°C ~1000°C, and found that the granite had "negative damage" when the temperature was 100°C. Wu et al. [14] carried out Brazilian splitting test on the granite rocks of Qinghai Gonghe Basin from 25°C ~600°C, and arrived at a conclusion that the tensile strength of the rock increases firstly and then decreases with the increasing temperature, and the granite changes from brittlement to ductile at 400°C ~600. By utilizing DIC, cracks with different initiation mechanisms could be identified and analyzed to quantify the impact of flaw inclination on crack types [15, 16]. Gao et al. employed digital image correlation (DIC) to reveal rate-dependent variations in fracture time, toughness, and crack growth velocity and affirm that the stress intensity and crack tip positions can be effectively determined from displacement fields obtained through DIC [17]. The method employs traditional DIC for displacement measurement, reconstructs the displacement field at the discontinuity using a modified subset splitting technique, and employs thorough post-processing for precise quantification of crack identification and displacement jump measurement [18]. High temperature has a significant impact on the splitting characteristics of rocks. With the increase of temperature, the rock thermal damage gradually accumulates, and the strength of the engineering rock mass is further degraded. However, most of the above research results are based on thermal damage caused by the heating process, while geothermal development is a heat transfer process of high-temperature rocks. Therefore, the study of the splitting characteristics and fissure evolution law of high temperature granite plays a significant role in the safety assessment and construction of geothermal engineering.

The granite after high temperature treatment analyzed the P-wave velocity, load-displacement curve and tensile strength of Brazil under different temperatures (25°C ~1050°C). At the same time, DIC technology is used to record the splitting process, describe the fracture initiation and expansion process, and establish the mathematical model of P-wave velocity and Brazilian tensile strength with temperature. The research results can provide a reference basis for the prediction of rock splitting characteristics of geothermal engineering.

## 2. Test method

### 2.1 Sample preparation

**Fig 1:**
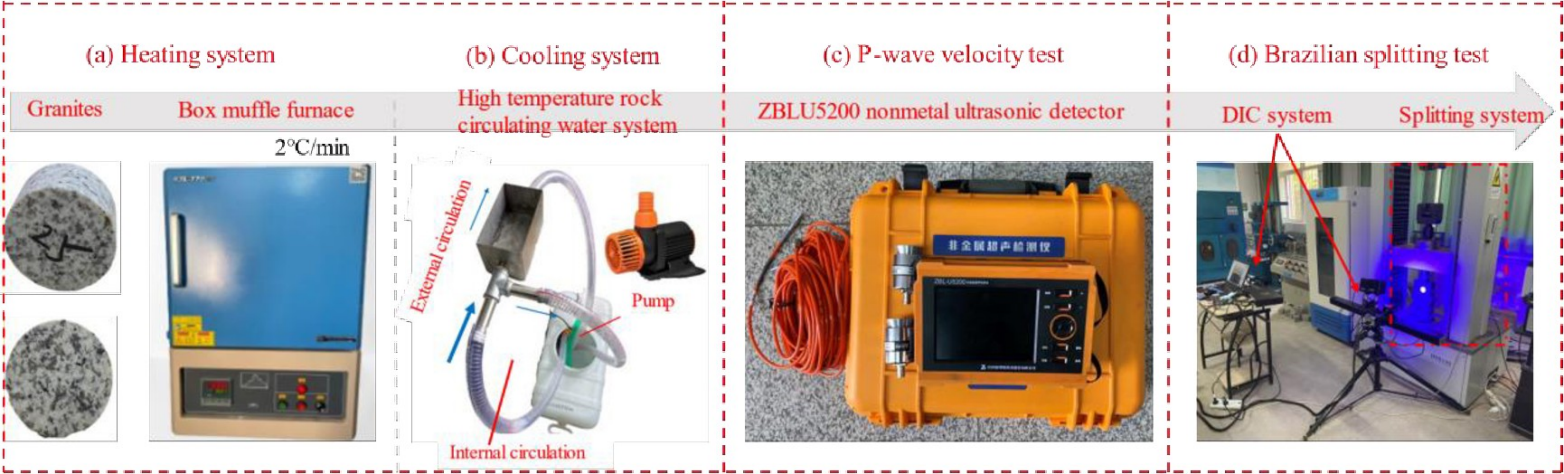
Testing setup.

The granite in the test was taken from Hubei Province, and all rock samples were drilled from the same granite block to reduce the discretization of the test results due to the inhomogeneity of the samples. The granite samples were grayish white, with uniform texture and without visible cracks. According to the X-ray diffraction results, the granite was mainly composed of 48.65% quartz, 17.53% sodium feldspar, 17.36% potassium feldspar, 5.23% calcium feldspar, 8.21% black mica and 3.02% other substances. According to the recommendations of the International Society for Rock Mechanics (ISRM), the basic physical and mechanical parameters of granite samples were obtained through tests, as shown in Table 1. The Brazilian disc samples with a diameter of 50 mm and a height of 25 mm (Fig 1A) were labeled according to the target temperature of the thermal treatment.

**Table 1:** Basic properties of the granite sample.

Properties	Values
Density, ρ (kg/cm^3^)	2616.48
P-wave velocity, *V*p, (km/s)	4.36
Porosity, *p*, (%)	0.57
Elastic modulus, *E* (GPa)	52.97
Tensile strength, *σ*_t_ (MPa)	8.87
Uniaxial compressive strength, UCS (MPa)	216.44

### 2.2 Test process

The testing process was mainly composed of a thermal treatment system, a P-wave testing system, a loading system, and a DIC system, as shown in Fig 1. The loading system and the DIC system worked synchronously during the experiment, and the relationships between the Brazilian splitting behavior and the surface deformation field of the samples were obtained in real-time.

#### 2.2.1 Thermal treatment system

The rock sample was heated with KSL-1700X high-temperature box mav furnace. The heating rate was set at 2°C/min and the target temperature was set at 150°C, 300°C, 450°C, 600°C, 750°C, 900°C and 1050°C respectively, as shown in Fig 1(A). In order to simulate the geothermal exploitation process of dry hot granite, the homemade high temperature rock circulating water system was used to sharply cool the high temperature granite rock. This process might cause some damage to the rock samples. In the high temperature rock circulating water system, the external circulation was mainly used to reduce the temperature of the high temperature granite, while the internal circulation was used to keep the temperature in the tank constant, so that it could not be affected by the heat from the external circulation [15]. To keep the water temperature in the tank the same as the room temperature, the water should be kept in the laboratory for 12 hours at least in advance before the test. Took the rock sample with the target temperature of 150°C as an example, the rock sample was first placed in the maver furnace at a heating rate of 2°C/min. After the furnace temperature reached 150°C, the constant temperature was treated for 2 hours, so that the sample could be completely heated to 150°C. During the constant temperature, open the high temperature rock circulating water system. After the constant temperature, the high temperature rock was placed in the high temperature chamber until cooled to the water temperature, as shown in Fig 1(B). Finally, the cooled sample was dried for the subsequent P-wave speed test and the Brazilian split test.

#### 2.2.2 P-wave testing system

After thermal treatment, the samples were dried to eliminate the influence of moisture on rock properties. Vertical wave velocity was sensitive to hot damaged rock by ZBLU5200 non-metal ultrasonic test. For good coupling between the sensor and the sample, vaseline was applied between the sensor and the sample.

#### 2.2.3 Brazilian splitting testing system

After nondestructive test of P-wave velocity, the splitting test was used to test the tensile properties of thermal damage rock. The loading equipment was a DNS electronic universal testing machine, which was loaded by displacement control, and the loading rate was set to 1 mm / min. In order to monitor the rupture, cracking and extension of rock during the test, DIC was used to monitor the split process. In the test device, the displacement field monitoring equipment was opened while the sample was loaded, keeping them synchronized to start operation. During the loading process, the test machine recorded the axial load and displacement of the disk sample in real time. The tensile strength of the sample was related to the axial load when the sample rupture. The expression is [16–18]:

$$\sigma_{t}=\frac{2P}{\pi Dh}$$ (1)

Where, *σ*_t_ is the tensile strength of the sample, MPa; *P* is the axial load when the sample breaks, kN; *D* is the diameter of the disc sample, mm; *h* is the thickness of the disc sample, mm.

#### 2.2.4 DIC system

DIC is a method to match the corresponding points on the surface image of the object being measured [19]. By collecting the relevant changes of the speckles (natural or artificial) images on the surface of the object, matched the images before and after according to the time sequence, and used the changes of the speckles to obtain displacement and deformation [20, 21]. Took the speckle pattern before the object deformation as the reference image, matched the deformed speckle pattern with it, and obtained the deformed image after successful matching. Circled a square area with a certain side length in the reference image as the reference area. Assumed that the coordinates of any two points in the reference area are M (*x*_0_, *y*_0_) and N (*x*_i_, *y*_i_), the corresponding area was the target area, and the deformed point coordinates were M ’(*x*_0_’, *y*_0_ ’) and N’ (*x*_i_ ’, *y*_i_ ’). Through a certain search method and calculation according to the correlation function, the displacement and strain of the point in the area could be obtained [22].

**Fig 2:**
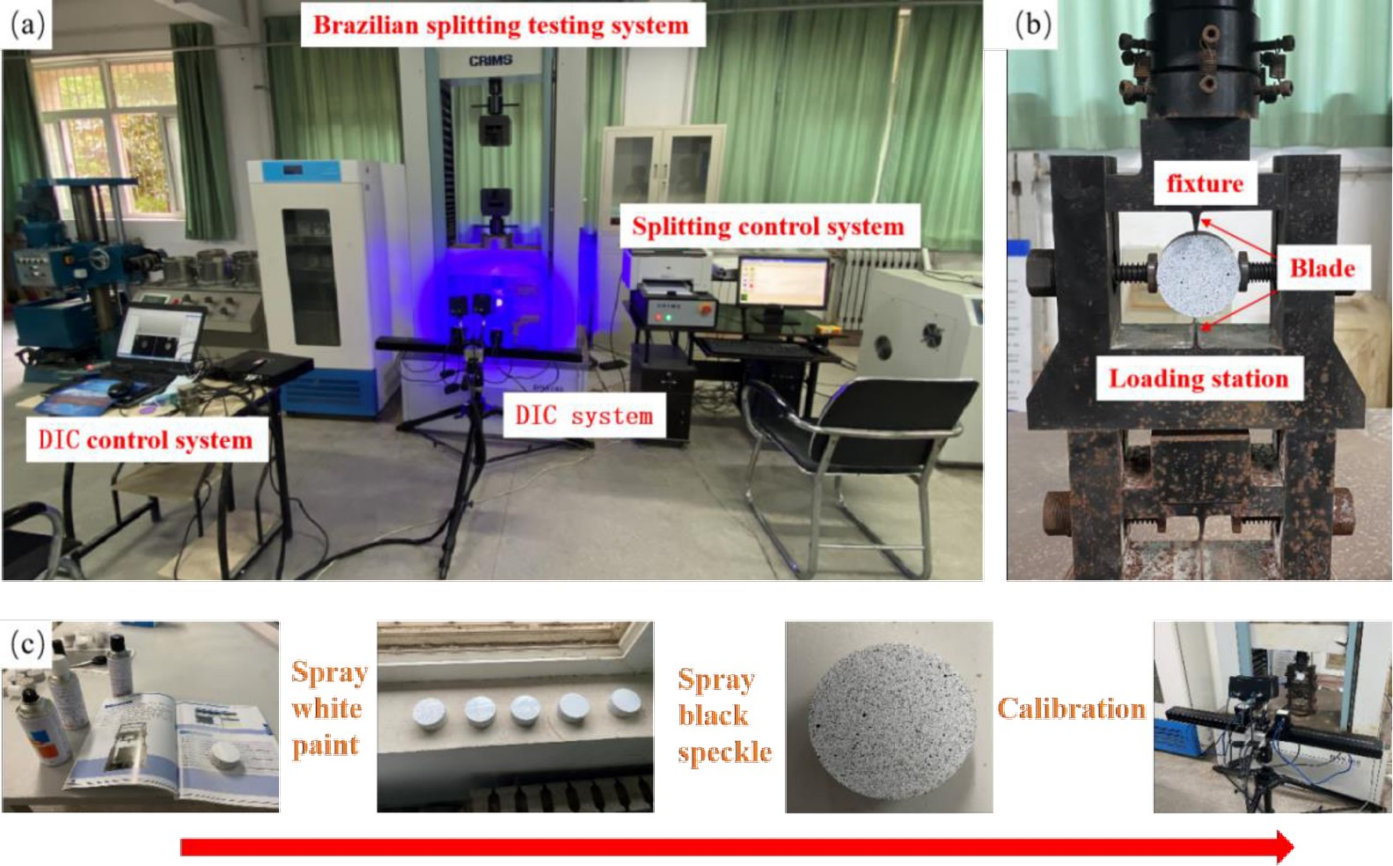
Testing setup for the Brazilian splitting test.

Before using the DIC system to measure, it was necessary to spray the Brazilian disc with speckle. The purpose of spraying speckles was to obtain a high-contrast gray distribution image. The quality of the speckle had a great impact on the test results. As shown in Fig 2, cleaned the surface of the rock samples to remove attachments first, and then white-hot matte paint shall be uniformly sprayed as the base material layer. Finally, the black matte paint was randomly sprayed. After each painting, let the samples stand for 20 minutes to dry the base material layer and the speckle. Primarily, the camera was stabilized on a tripod, precisely positioned two meters in front of the surface of the rock sample for optimal capture. To ensure ample illumination, each side of the camera was equipped with an LED cold light source. The camera was then meticulously calibrated to chronicle images at a frequency of 50 frames per second, a process that commenced simultaneously with the DNS loading and concluded only upon the specimen’s catastrophic failure. With an image resolution of 2048 × 2048 pixels and an actual sampling window measuring 164 × 164 mm, the resulting ratio of actual length to image pixels was fine-tuned to 0.08 mm/pixel. In view of calculating efficiency and precision, the subset size was assigned 20 pixels with a step size of 7 pixels. This setup affirmed a displacement measurement accuracy of 10–20 μm within the DIC system. Exploiting the correlation algorithm, the deformation image was matched and analyzed against its post-deformation counterpart. This comparison facilitated an insight into the comprehensive deformation characteristics of the specimens.

## 3. Results and analysis

### 3.1 Variation law of the P-wave velocity

**Fig 3:**
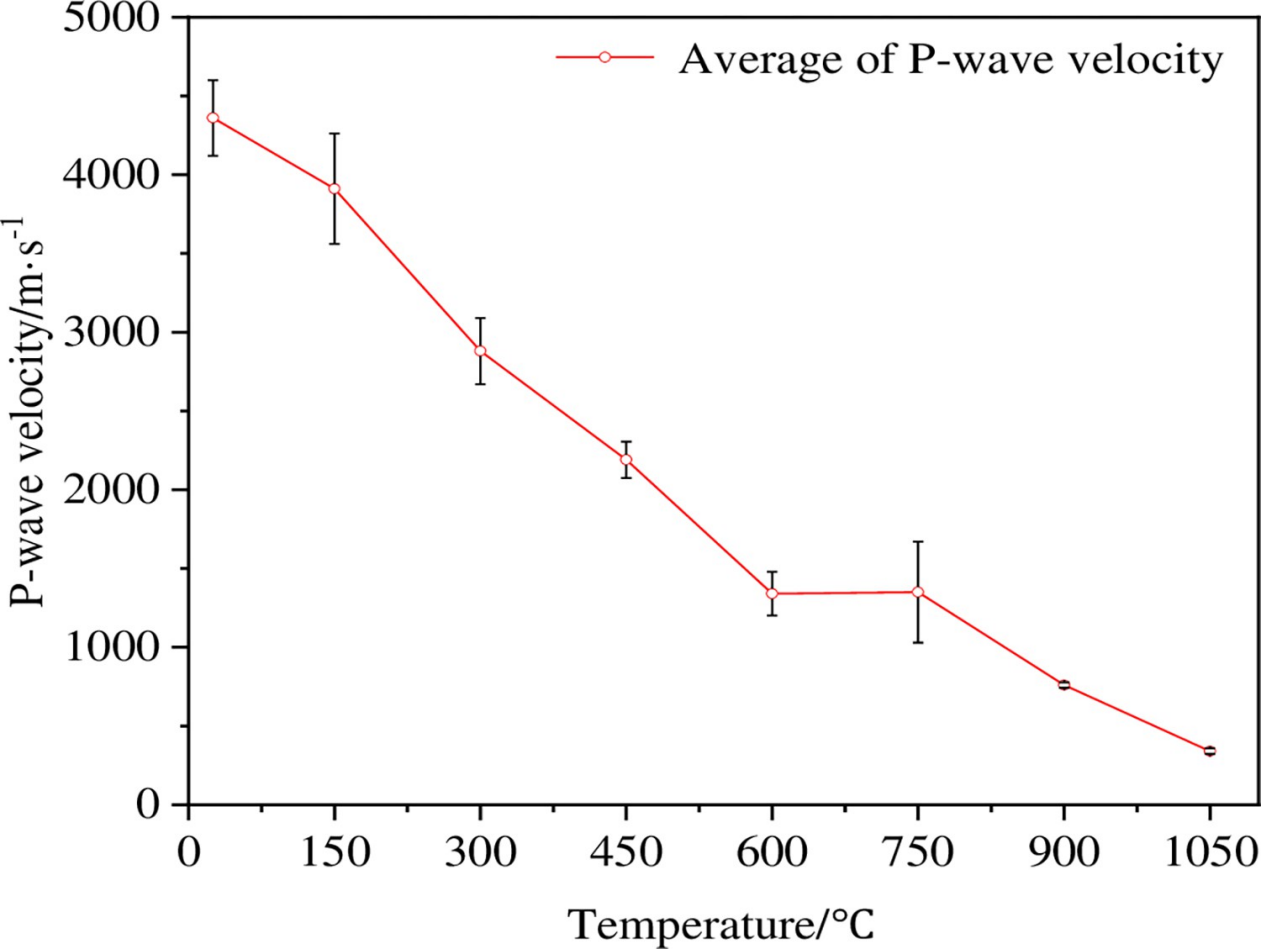
Variation curve of the P-wave velocity of high-temperature granite under water-cooling.

The P-wave velocity can reflect the change degree of microstructure inside the rock, especially for the development of micro-cracks, mineral composition change and bonding degree of thermal damage rock [23], so the P-wave velocity is an important indicator to evaluate the damage degree of dry hot granite. Fig 3 shows the change of the P-wave velocity after the thermal impact of the granite at different temperatures, where the change curve shows the average wave velocity of the three samples at the same temperature, and the test results of the other samples are shown in Table 2. As can be seen from Fig 3, the degree of thermal damage at different temperatures has a significant impact on the P-wave velocity of the rock. As the thermal treatment temperature increases from room temperature to 1050°C, the average P-wave speed of the rock samples rapidly decreases from 4360 m / s to 340 m / s. When the thermal treatment temperature becomes higher than 600°C, the reduction rate of the average P-wave velocity of the sample begins to decrease, which indicates that the changes in the internal structure of the rock mainly occurs before 600°C. When the thermal treatment temperature is between 450 and 600°C, the average P-wave speed reduction rate of the sample reaches the maximum value, indicating that the internal structure of the sample may have been greatly changed in this temperature range, and there turns out to be a threshold to change the mechanical properties of the rock.

**Table 2:** P-wave velocity and tensile strength of high-temperature granite under water-cooling.

Temperature	P-wave velocity/m·s^-1^				Brazilian tensile strength/MPa			
	Sample 1	Sample 2	Sample 3	Average	Sample 1	Sample 2	Sample 3	Average
25	4360	4120	4600	4360	9.37	8.87	8.37	8.87
150	4300	3810	3620	3910	12.04	11.04	10.04	11.04
300	3060	2930	2650	2880	6.84	6.85	6.5	6.73
450	2280	2230	2060	2190	5.02	5.1	5.12	5.78
600	1260	1500	1260	1340	1.82	1.45	1.61	1.63
750	1510	1560	980	1350	1.36	1.26	0.76	1.13
900	750	750	780	760	0.94	0.84	0.34	0.71
1050	350	350	320	340	0.72	0.12	0.62	0.49

### 3.2 Change of the tensile stress displacement curve

**Fig 4:**
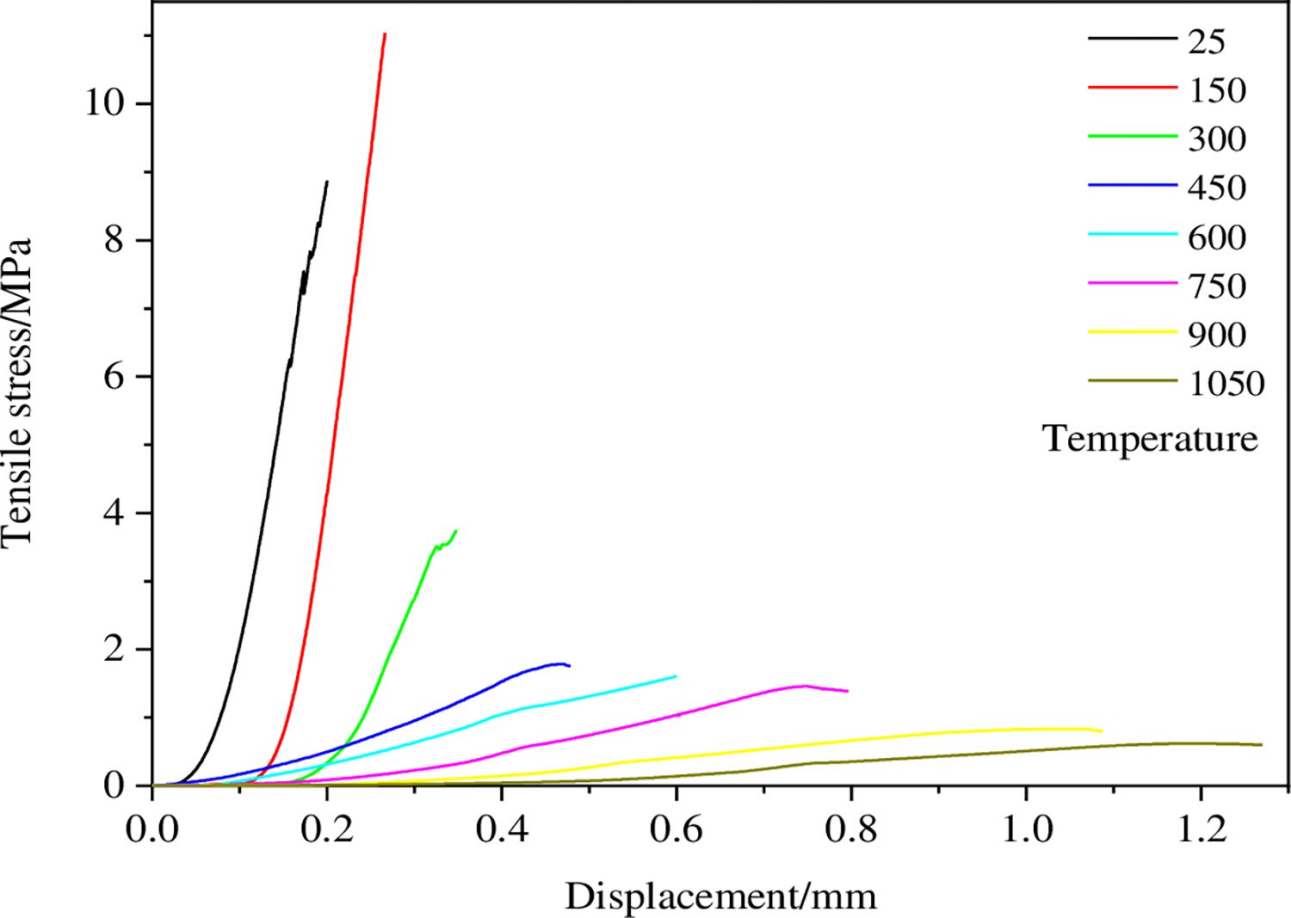
Tensile stress displacement curve of high-temperature granite under water-cooling.

According to the results of rock P-wave velocity, the internal structure of dry and hot granite varies at different temperatures. The change of the internal structure of the rock can cause the change of the macroscopic mechanical characteristics of the rock inevitably, which can make the rock samples show different stress-displacement curves in the splitting process. Fig 4 shows the representative tensile stress-displacement curve of the rock samples with different thermal treatments during the splitting process. The effect of thermal damage on the Brazilian splitting behavior of dry-hot granite can be seen from the tensile stress-displacement curve. As can be seen from the figure, there are nonlinear parts in the stress curve of the samples at different temperatures, which are mainly caused by the micro-cracks inside the rock [24]. In addition to the original fissures, there are also new micro-fissures formed by the influence of temperature. The two kinds of fissures control the stress curve of the sample under the load jointly. Furthermore, the non-linearity of the tensile stress-displacement curve gradually increases with the increasing temperature of the thermal treatment on the sample. Overall, the stress curve shows weak non-linearity before the peak, and the pre-peak curve becomes more gradual with the increasing temperature, indicating that the increasing temperature could enhance the ductility characteristics of dry-hot granite.

### 3.3 Change of Brazilian tensile strength

**Fig 5:**
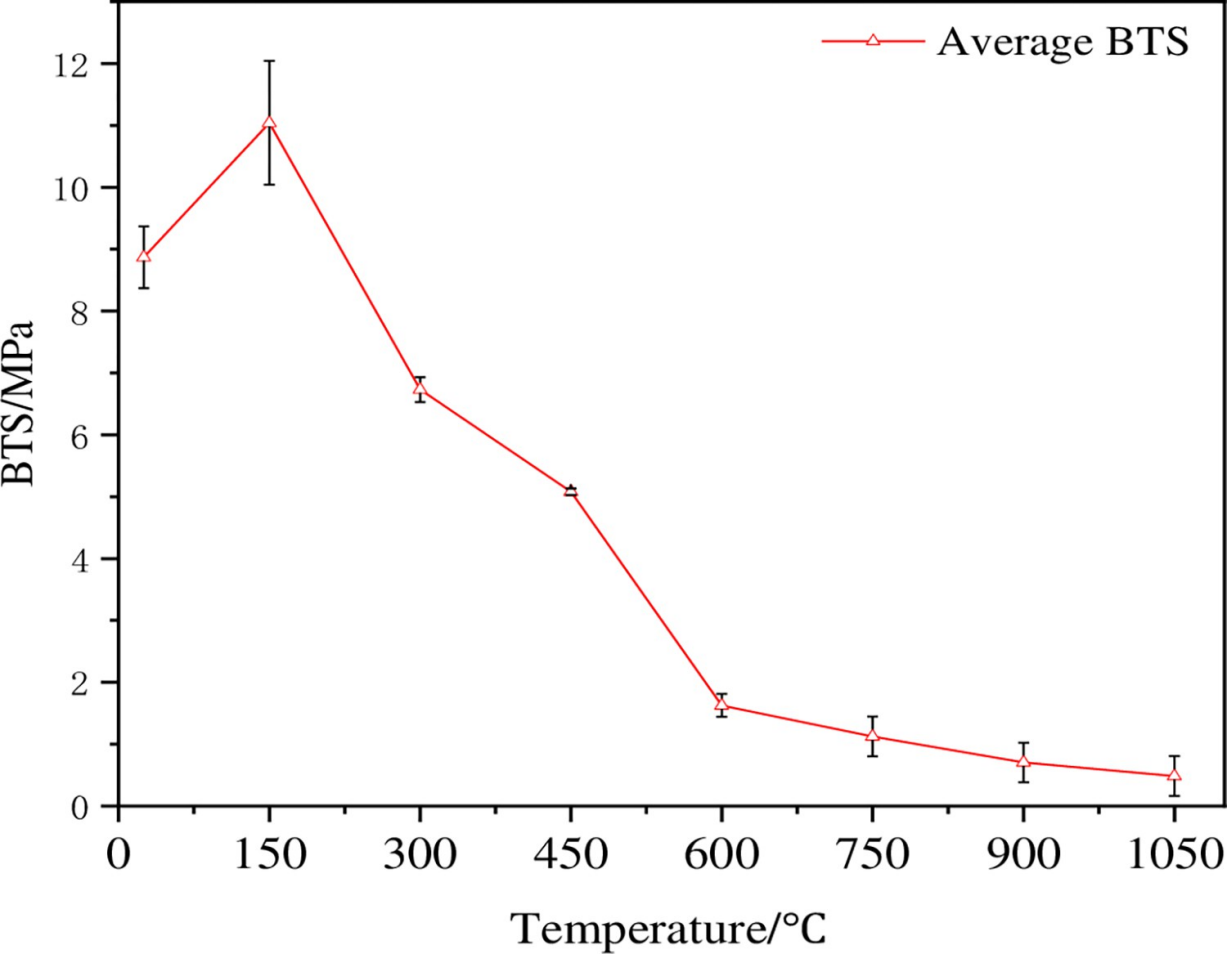
BTS change curve of high-temperature granite under water-cooling.

Fig 5 shows the changing characteristics of Brazilian tensile strength (BTS) of dry hot granite at different temperatures with the increasing temperature. The test data are shown in Table 2. The change curve of BTS of the samples with the increasing temperature is different from the change trend of P-wave velocity, and the change between 25 and 150°C turns to be different. The reason for this phenomenon is complex, and the water, mineral composition and particle structure changes can occur under the influence of temperature. When the temperature is 150°C, the free water inside the rock escapes, and the expanding mineral particles make the primary fissure close. When the thermal stress generated by the mineral expansion is insufficient to damage the rock structure, the enhancement of the rock strength is the thermal hardening of the rock [25], and the temperature of 150°C produces the thermal hardening phenomenon. The BTS curve in Fig 5 shows a trend of local increase at a temperature of 150°C, which verifies the existence of the thermal hardening phenomenon. The change of the P-wave velocity of the sample at 150°C is mainly due to the comprehensive influence of various factors such as the elastic modulus and density inside the rock. It is obvious that the sensitivity of the P-wave velocity to the elastic modulus and density is stronger than the structural change caused by the expansion of mineral particles. After further warming, BTS of the sample begins to decrease gradually. As the temperature increases from 150°C to 1050°C, BTS in the test decreases by 98.56%. When the temperature exceeds 600°C, the change tendency of the sample BTS is similar to that of the P-wave speed, the reduction rate begins to decrease, and the change curve becomes flat. Studies shows that BTS of the sample is significantly reduced between 450 and 600°C. When the temperature exceeds 900°C, the effect of it increases on BTS of the sample can be ignored, and the sample has lost most of its mechanical strength.

### 3.4 Analysis of rock cracking

In recent years, the research on the cracking situation in the process of rock splitting has attracted the attention of scholars both domestically and internationally. Due to the brittle characteristics of rock materials, it is difficult to achieve direct tensile measurement, so the Brazilian splitting method is used widespread to measure the tensile strength indirectly. When testing the rock strength by the Brazilian splitting method, assuming the rock damage is obedient to the Griffith strength criterion [26], that is,

$$\sigma_{t}=-\frac{{\left(\sigma_{1}-\sigma_{3}\right)}^{2}}{8(\sigma_{1}+\sigma_{3})}$$ (2)

Where, *σ*_1_ and *σ*_3_ are the maximum and minimum principal stress of the rock, respectively, MPa.

**Fig 6:**
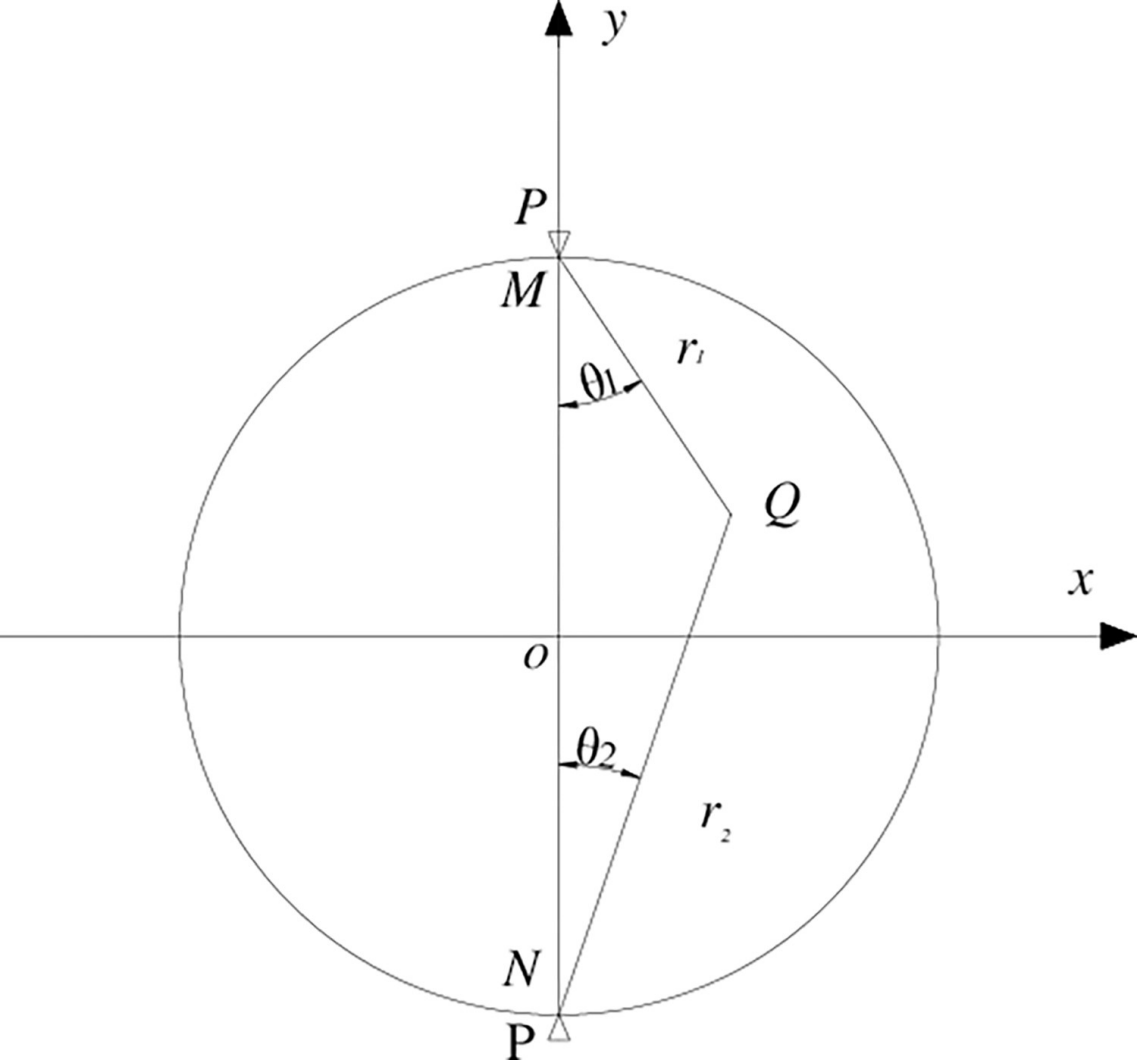
Two-dimensional plane stress diagram of disc sample.


Strength formula of rock Eq (1) can be obtained from Eq (2). The default condition of Eq (1) is that the sample is broken first by the center point of the disc. However, the researchers found that the first crack did not necessarily crack from the center point of the disc, and even improved the fixture of the split tester, but the phenomenon of non-disc center crack still existed [27–29]. The stress distribution of the disk sample in the Brazilian split test has a significant effect on the crack onset. The position of crack is analyzed from the perspective of sample stress distribution. The stress distribution of the disc sample in the Brazilian split test has a significant influence on crack initiation. The crack initiation position from the perspective of the sample stress distribution was analyzed in this paper. As shown in Fig 6, when the disk receives a radial force, the stress component of any point *Q* on its surface can be expressed as [30, 31]:

$$\sigma_{x}=\frac{2P}{\pi h}(\frac{\sin^{2}\theta_{1}\cos\theta_{1}}{r_{1}}+\frac{\sin^{2}\theta_{2}\cos\theta_{2}}{r_{2}})-\frac{2P}{\pi Dh}$$ (3)

$$\sigma_{y}=\frac{2P}{\pi h}(\frac{\cos^{3}\theta_{1}}{r_{1}}+\frac{\cos^{3}\theta_{2}}{r_{2}})-\frac{2P}{\pi Dh}$$ (4)

$$\tau_{x}=\frac{2P}{\pi h}(\frac{\cos^{2}\theta_{1}\sin\theta_{1}}{r_{1}}+\frac{\cos^{2}\theta_{2}\sin\theta_{2}}{r_{2}})$$ (5)

Where, the stress component represents the uniformly distributed stress in the two-dimensional plane, in which *σ*_*x*_ is the tensile stress in the *x* direction; *σ*_*y*_ is the tensile stress in the y direction of the two-dimensional plane; *τ*_*x*_ is the shear stress in the *x* direction, and the direction of tensile stress is negative.

The stress analysis of point at the center of the disc *o* in Fig 6 shows that

$$\sigma_{x}=-\frac{2P}{\pi Dh}$$ (6)

$$\sigma_{y}=-\frac{6P}{\pi Dh}$$ (7)

Substitute Eq (6) and Eq (7) into the Griffith strength criterion Eq (2), and Eq (8) can be obtained as:

$$-\frac{{\left(\sigma_{y}-\sigma_{x}\right)}^{2}}{8(\sigma_{y}+\sigma_{x})}=\frac{2P}{\pi Dh}=\sigma_{t}$$ (8)

**Fig 7:**
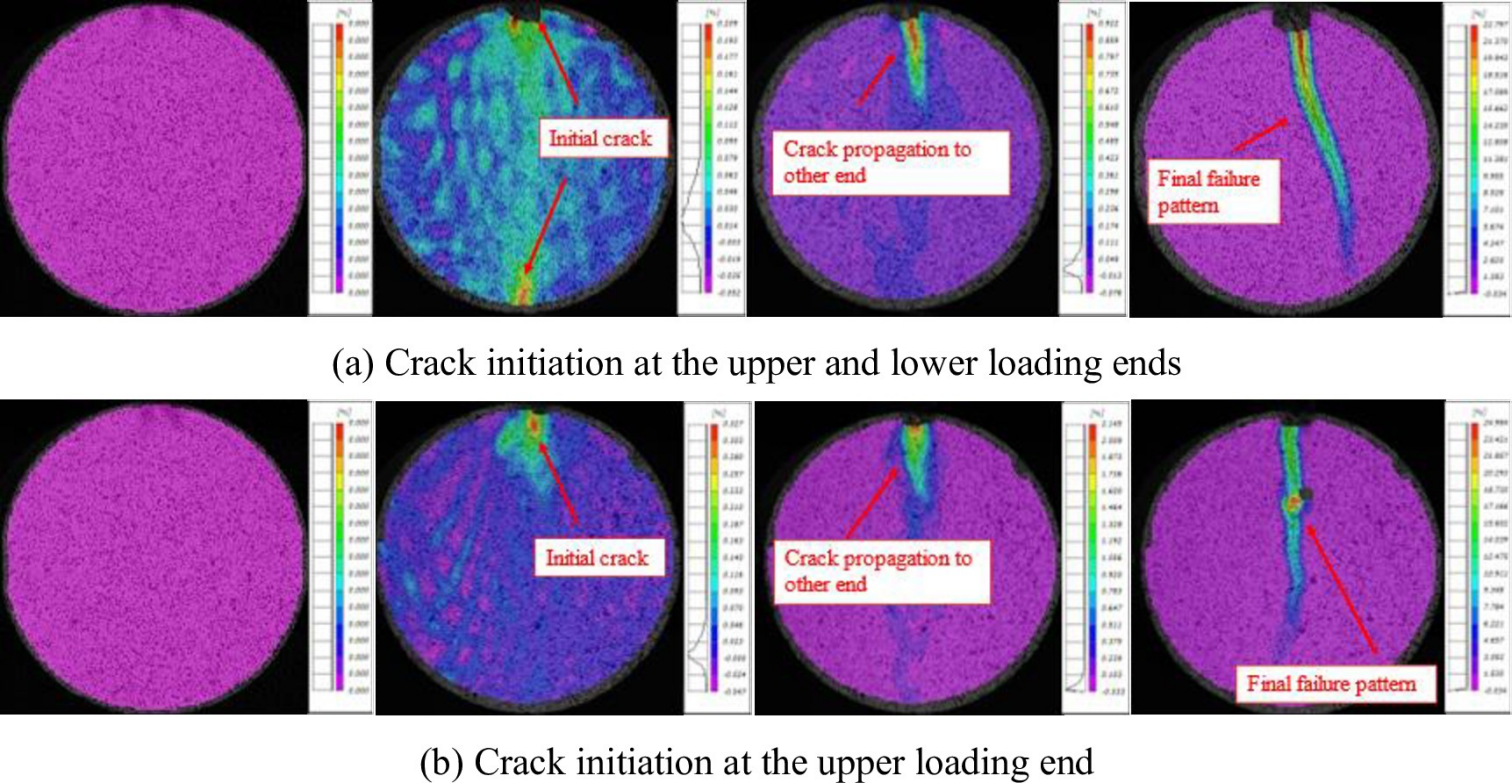
Typical split failure process of high-temperature granite under water-cooling.

Therefore, the horizontal tensile stress value at the center of the disk reaches the tensile strength of the rock firstly and then breaks up. However, when using the conclusion of the center of the disk, it is ignored that the disk stress distribution formula is derived based on the two-dimensional plane stress, while the actual rock is in a three-dimensional solid stress state. More importantly, the plane stress condition requires that the stress in the same direction on the disk should be distributed evenly, which requires a very high homogenization of the disc material [32, 33]. The sample in this paper is a rock sample after thermal treatment, and the internal structure of the rock has changed significantly through the preliminary P-wave speed test. It is obvious that the sample dissatisfy the preconditions required by Eq (8). Therefore, the phenomenon of non-disc center cracking occurs during splitting on dry-hot granite. In order to explore the location of crack initiation caused by tensile stress after dry hot granite encountered thermal shock, DIC monitoring equipment is used in this study to monitor the crack initiation, expansion and failure of dry hot granite samples during the splitting process. The results show that the initial cracking position of the sample is not the center of the disk, but the loading point at both ends of the sample. In general, when the sample temperature is between room temperature and 450°C, the sample is broken by the loading point at both ends of the disk, and then the crack of the upper loading point continues to extend down the loading point, and the crack extension of the loading point is not obvious, as shown in Fig 7(A). When the sample temperature is between 450°C and 1050°C, the failure of the sample breaks from the loading point at the upper end of the disk, and the crack gradually extends down the loading point from the upper loading point until the rock sample is completely destroyed.

It can be seen from Fig 7 that the crack onset and cracking point of the dry hot granite after thermal shock is the loading point in the Brazilian splitting tests, rather than the sample disk center. The homogenization of the disk material has a significant effect on the plane stress distribution, and the change of the sample homogeneity is mainly caused by the thermal stress. With the increase of thermal treatment temperature, the thermal stress inside the sample is increasing, leading to the change of rock micro-structure at the same time, and accompanied by the production of many micro-cracks. This indicates that under the influence of thermal shock (rapid hot and cold treatment), the rock surface damage is caused to form a micro-crack, resulting in the crack initiation more likely to happen on the loading end. When the temperature is between room temperature and 450°C, the sample is forced simultaneously in the radial direction, and the upper and lower loading points crack at the same time. However, when the temperature is between 450°C and 1050°C, the number and density of micro-cracks inside the sample are not enough to transfer stress in the radial direction, resulting in crack-onset condition happening only on the upper loading point. Therefore, changes in the internal structure of the sample can affect the disk stress distribution and thus change the crack initiation position.

### 3.5 Analysis of the micro-structure of the rock

**Fig 8:**
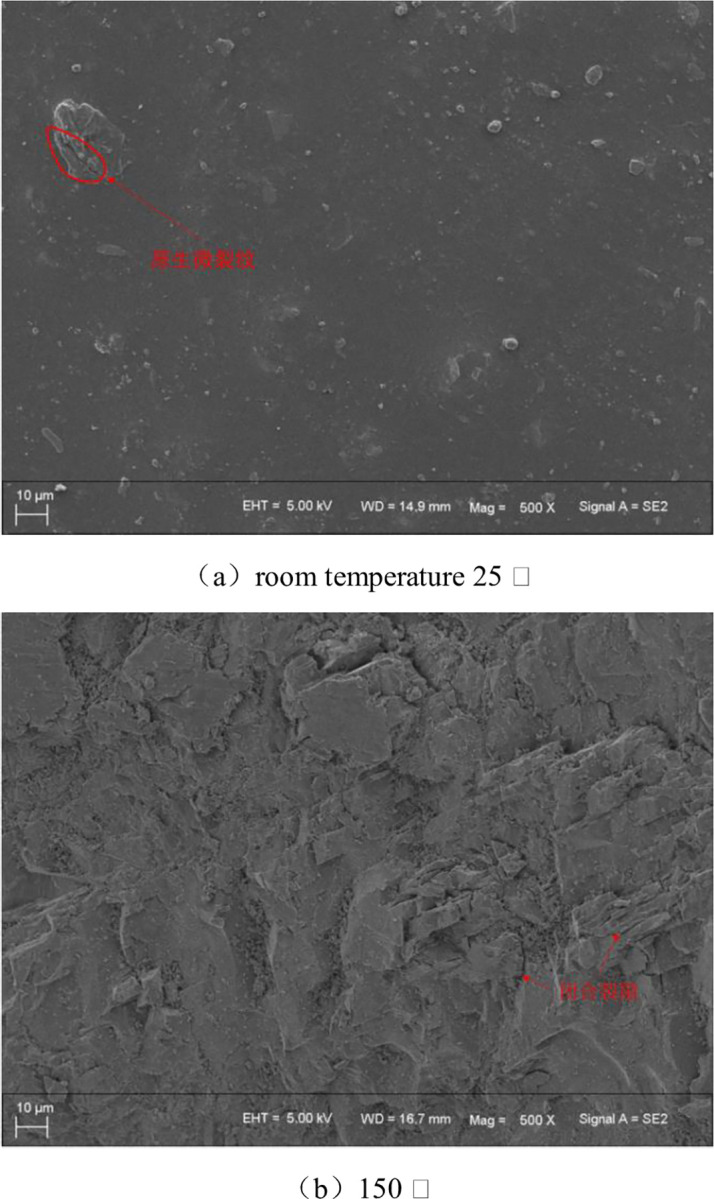
SEM observation diagram of rock samples.

The cracking position of hot-damaged granite is affected by the development of micro-cracks within the rock. Different degrees of thermal damage cause different numbers and types of micro-cracks inside the rock [34, 35]. In order to study the development of internal micro-cracks in different thermal-damaged granite, the granite samples with different temperature thermal treatment were sectioned and carbon sprayed, and the internal structure was observed by scanning electron microscopy (SEM). SEM observations of room temperature granite samples are shown in Fig 8(A), the mineral particles of the rock samples are closely bound with only minimal primary cracks. After 150°C of granite treatment, the primary micro-cracks in the rock sample mineral are locally closed by thermal stress, and some of the pores disappeared. This phenomenon is only reflected in the overall tensile strength of the rock sample, and there is still uneven deformation in the rock, leading to the change of the cracking position of the rock, as shown in Fig 8(B). After the granite treated after the high temperature of 300°C, a small amount of inter-crystalline cracks emerge inside the rock mineral, and the connections between these minerals are relatively close. Some perforating cracks can be observed that pass through the feldspar mineral and eventually through the black mica mineral, connected to the inter-crystalline cracks at the boundary between the feldspar and quartz minerals. However, the penetration cracks failed to penetrate the quartz minerals. At this temperature, the crystalline cracks dominate the three kinds of micro-cracks (internal cracks, inter-crystal cracks and penetrating cracks), but the extension length and opening width of the crystal cracks are small and only act inside the mineral particles. This has a limited effect on the tightness of mineral particle binding and limited changes in the physical and mechanical properties of granite.

## 4. Discussion

**Fig 9:**
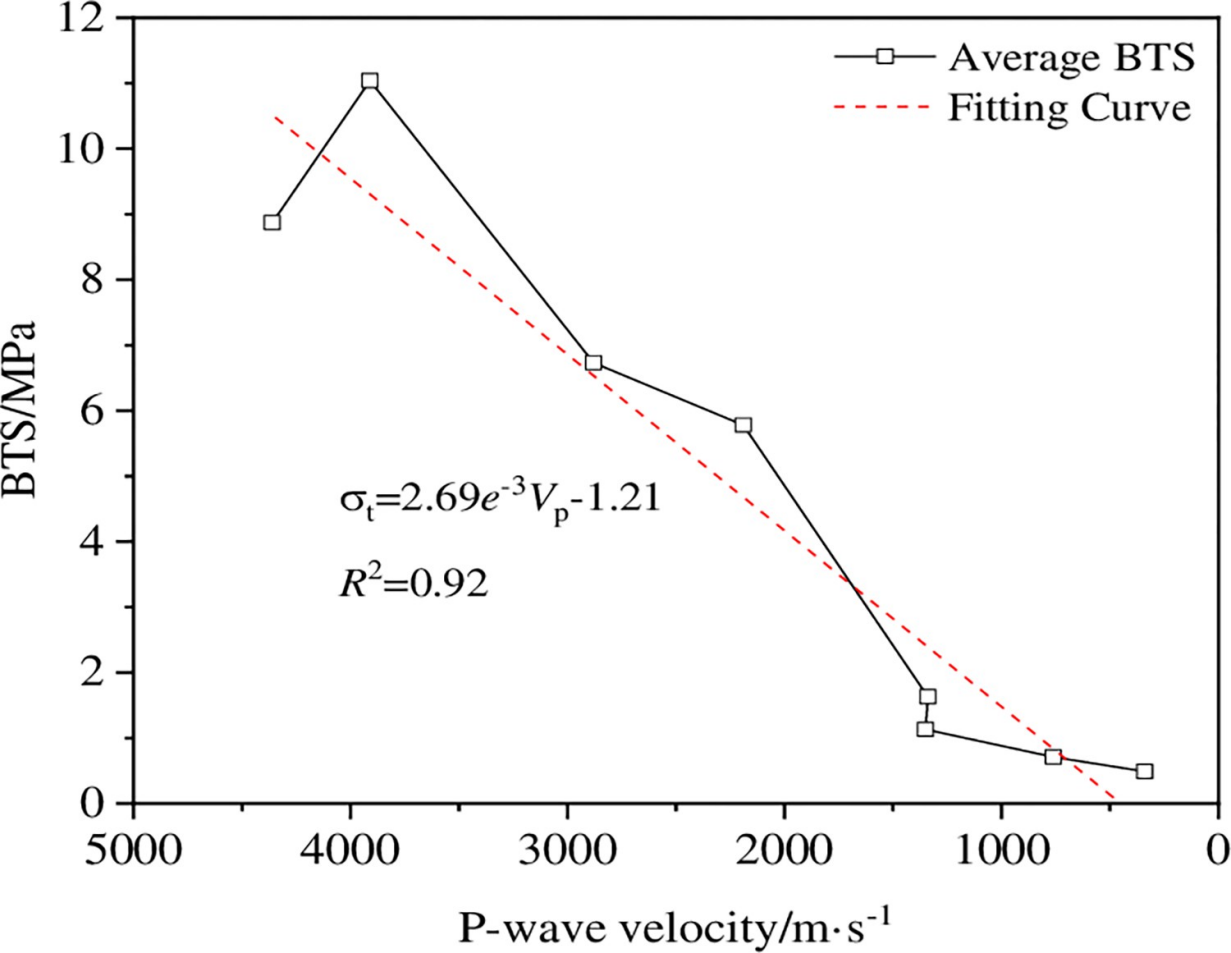
Relation curve between the tensile strength of the sample and the P-wave velocity.

The P-wave velocity and BTS test after thermal shock on room temperature ~1050°C dry-hot granite show that 450~600°C is the threshold temperature for dry-hot granite damage. The changes of the P-wave speed and tensile strength of the sample are controlled by the internal micro-structure of the rock. In the temperature range from 450 to 600°C, the mineral particles produce a phase transition and form a certain quantity of micro-cracks, resulting in a sharp decrease in the P-wave velocity and tensile strength of 38.81% and 71.80%, respectively. When the temperature exceeds 750°C, the cumulative damage of the rock reaches the peak, and the P-wave velocity and tensile strength decrease slowly and stabilize gradually. Therefore, the wave speed and tensile strength of the sample can be expressed by mathematical model, as shown in Fig 9. It can be seen from the figure that the tensile strength of the sample decreases as the P-wave velocity decreases. The correlation coefficient of 0.92 is obtained by fitting the linear model, indicating that there is a linear relationship between them.

In order to describe the damage evolution of high-temperature granite under different temperatures during geothermal exploitation quantitatively, two empirical formulae are proposed to predict the change of P-wave velocity and tensile strength with the increasing temperature. Figs 3 and 5 show that the P-wave speed and tensile strength of the test sample gradually decrease as the thermal treatment temperature increases. Therefore, negative exponential model functions are established in the paper:

$$V_{p}=V_{m}\cdot e^{-T/n}+m$$ (9)

$$\sigma_{{}_{T}}=\sigma_{m}\cdot e^{-T/n}+m$$ (10)

Where, *V*_*p、*_ *σ*_*T*_ are the velocity of P-wave and BTS at different temperatures predicted by the model respectively; *V*_*m*_、 *σ*_*m*_ are the maximum velocity of P-wave and the BTS respectively; *T* is the temperature of thermal treatment of the sample; m、n are the model parameter, which control the degree and height of the curve respectively.

**Fig 10:**
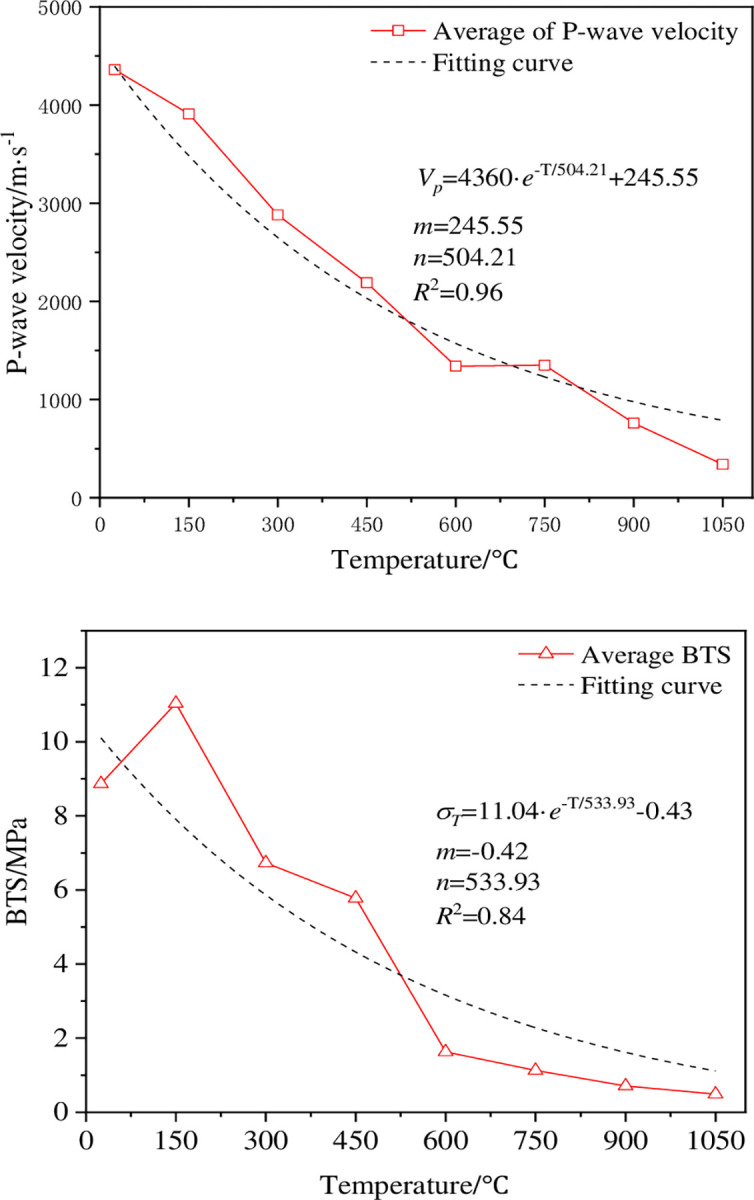
Comparison between test results and model predictions: (a)P-wave (b)BTS.

Fit the results of the sample P-wave velocity and BTS test by Eq (9) and Eq (10), and the fitting results are shown in Fig 10. In the figure, the P-wave velocity of the rock and BTS are not consistent at 150°C. This is because when the temperature is 150°C, the thermal stress promotes the primary fissure closure due to the thermal expansion of the mineral particles in the rock, which makes the rock interior tighter, and the porosity decreases, while the tensile strength increases. However, the change of the P-wave velocity is not only dependent on the porosity of the rock, but also affected by other factors. Specifically, high temperature can reduce and order the crystalline defects in mineral crystals, thus reducing the friction between crystals and the elastic modulus. At the same time, high temperature can also make the gas and liquid in the rock discharge, resulting in a reduction in the density inside the rock, which can also reduce the P-wave speed. Therefore, the decrease of P-wave velocity can be regarded as a reaction of changes in rock structure and physical properties.

Therefore, it can be seen that when the temperature exceeds 150°C, the wave speed of dry hot granite vertical wave and the theoretical curve of BTS fit the test results well, which shows that the negative exponential model established in this paper can accurately describe the changes of P-wave speed and BTS with temperature after thermal shock of high-temperature granite.

## 5. Conclusions

1. The thermal treatment process causes different degrees of thermal damage to the high-temperature granite, which has a significant impact on both the P-wave velocity and the BTS. In the process after thermal shock, the P-wave velocity and BTS decrease gradually with the increase of thermal treatment temperature. The decrease in P-wave velocity and BTS is the most significant, especially in the temperature range from 450 to 600°C.
2. In the Brazilian splitting test, the presence of the micro-crack changes the stress transfer path, so that the stress cannot be distributed evenly on the surface of the disk, so that the starting point of the disk crack starts from the loading point. When the temperature is below 450°C, the crack cracks from the upper and lower loading end, while when the temperature is above 450°C, the crack cracks from the upper loading end.
3. There is an obvious linear relationship between BTS and P-wave velocity. The negative exponential function model proposed in this paper can describe the changing relationship of BTS and P-wave velocity with the increase of temperature well, which provides a theoretical basis for the prediction of splitting characteristics after the thermal shock of high-temperature granites.
4. Under the action of thermal shock, a large number of microcracks are generated inside the high-temperature granite, changing the internal structure of the rock and ultimately causing a change in the initiation point of the rock during the Brazilian splitting process, revealing the modification effect of thermal shock on the high-temperature granite.

## Supporting information

S1 Data (ZIP)

S2 Data (ZIP)

S1 File (OPJU)

S2 File (XLSX)

S3 File (OPJU)

S4 File (OPJU)

S5 File (OPJU)

## References

[1] Feng Z.J., Xi B.P., Zhao J.C, Zhao Y.S, Wan Z.J, Wu Z.W, Study on the principle of geothermal resource exploitation technology in hot dry rock [J]. China Science and Technology Achievements, 2018(11):32–34.

[2] Zhou Z., Jin Y., H Lu Y., Zhou B.C., Present challenge and prospects of drilling and hydraulic fracturing technology for hot dry rock geothermal reservoir [J]. Scientia Sinica Physica, Mechanica & Astronomica, 2018, 48(12):97–102.

[3] Li S.T., Zhang S.Q., Jia X.F., Xu T.F., Ren T., Li F.C., Index system research of project site selection for hot dry rocks exploration [J]. GEOLOGICAL SURVEY OF CHINA,2018,5(02):64–72.

[4] Tang Shibin; Wang Jiaxu; Chen Peizhao. Theoretical and numerical studies of cryogenic fracturing induced by thermal shock for reservoir stimulation. INTERNATIONAL JOURNAL OF ROCK MECHANICS AND MINING SCIENCES, 125: 104160

[5] Li C., Hu Y.Q., Zhang C.W., Zhao Z.R., Jin P.H., Hu Y.F., et al., Brazilian split characteristics and mechanical property evolution of granite after cyclic cooling at different temperatures [J]. Chinese Journal of Rock Mechanics and Engineering,2020,09(39):1797–1807.

[6] Qian Yin, Jiangyu Wu, Chun Zhu, Qi Wang, Jinyong Xie. The role of multiple heating and water cooling cycles on physical and mechanical responses of granite rocks. Geomechanics and Geophysics for Geo-Energy and Geo-Resources, 2021, 7: 69.

[7] Qian Yin, Richeng Liu, Hongwen Jing, Haijian Su, Liyuan Yu, Lixin He. Experimental study of nonlinear flow behaviors through fractured rock samples after high-temperature exposure. Rock Mechanics and Rock Engineering, 2019, 52: 2963–2983.

[8] Yang S.Q., Ranjith P.G., Jing H.W., Tian W.L., Ju Y., An experimental investigation on thermal damage and failure mechanical behavior of granite after exposure to different high temperature treatments[J]. Geothermics, 2017,65:180–197.

[9] Tang S. B.; Tang C. A. Crack propagation and coalescence in quasi-brittle materials at high temperatures. ENGINEERING FRACTURE MECHANICS, 134: 404–432.

[10] Chen S.W., Yang C.H., Liu P.J., Wang G.B., Wei X., Laboratory simulation test of thermal cracking of Beishan granite [J]. Rock and Soil Mechanics,2016,37(S1):547–556. doi: 10.16285/j.rsm.2016.S1.071

[11] Zhang B.H., Liu W.F., Deng J.H., Liu J.F., Damage mechanism and stress wave spectral characteristics of rock under tension [J].Chinese Journal of Geotechnical Engineering,2016,38(S2):336–341.

[12] Wu S.C., Guo P., Zhang S.H., Zhang G., Jiang R.H., Study on thermal damage of granite based on Brazilian splitting test [J]. Chinese Journal of Rock Mechanics and Engineering,2018,37(S2):3805–3816.

[13] Fang X.Y., Xu J.Y., Liu S., Wang P., Research on splitting-tensile tests and thermal damage of granite under post-high temperature [J]. Chinese Journal of Rock Mechanics and Engineering,2016,35(S1):2687–2694.

[14] Wu Y.C., Xi B.P., Wang L., Niu X.M., Wang S., Zhao Y.S., Experimental study on physico-mechanical properties of granite after high temperature [J].Journal of Central South University (Science and Technology),2020,51(01):193–203.

[15] Liu L, Li H, Li X, et al. Underlying mechanisms of crack initiation for granitic rocks containing a single pre-existing flaw: insights from digital image correlation (DIC) analysis[J]. Rock Mechanics and Rock Engineering, 2021, 54: 857–873.

[16] Zhang L, Zhang Z, Chen Y, et al. Crack development and damage patterns under combined dynamic-static loading of parallel double fractured rocks based on DIC technique[J]. Acta Geotechnica, 2023, 18(2): 877–901.

[17] Gao G, Yao W, Xia K, et al. Investigation of the rate dependence of fracture propagation in rocks using digital image correlation (DIC) method[J]. Engineering Fracture Mechanics, 2015, 138: 146–155. 10.1016/j.engfracmech.2015.02.021

[18] Miao S, Pan P Z, Zhao S, et al. A new DIC-based method to identify the crack mechanism and applications in fracture analysis of red sandstone containing a single flaw[J]. Rock Mechanics and Rock Engineering, 2021, 54: 3847–3871. 10.1007/s00603-021-02472-5

[19] Wu X.H., Cai M.F., Ren F.H., Sun J.L., Guo Q.F., Wu X., et al., Evolutions of P-wave velocity and thermal conductivity of granite under different thermal treatments [J].Chinese Journal of Rock Mechanics and Engineering,2022,41(3):457–467.

[20] Li C., Hu Y.Q., Zhang C.W., Zhong Z.R., Jin P.H., Hu Y.F., et al., Brazilian split characteristics and mechanical property evolution of granite after cyclic cooling at different temperatures [J]. Chinese Journal of Rock Mechanics and Engineering,2020:1–11.

[21] Qian Yin, Jiangyu Wu, Chun Zhu, Manchao He, Qingxiang Meng, Hongwen Jing. Shear mechanical responses of sandstone exposed to high temperature under constant normal stiffness boundary conditions. Geomechanics and Geophysics for Geo-Energy and Geo-Resources, 2021, 7(2): 1–17.

[22] Tang S. B.; Huang R. Q.; Wang S. Y.; Bao C. Y.; Tang C. A. Study of the fracture process in heterogeneous materials around boreholes filled with expansion cement. INTERNATIONAL JOURNAL OF SOLIDS AND STRUCTURES, 112: 1–15

[23] Peters W., Ranson W.0. Digital Imaging Techniques in Experiment Stress Analysis[J]. Optical Engineering, 1982,21(3):213427.

[24] Ma S.P., Jin G.C., Zhao Y.H., A hybrid method for subpixel registration of digital speckle correlation method [J]. Optical techique,2005(06):72–75.

[25] Ma S.P., Jin G.C., Pan Y.S., Deformation measurement method for rock materials based on natural speckle pattern [J]. Chinese Journal of Rock Mechanics and Engineering,2002(06):792–796.

[26] Pan B., Kai L. A Fast Digital Image Correlation Method for Deformation Measurement[J]. Optics and Lasers in Engineering, 2011,49(7):841–847.

[27] Sun H., Sun Q., N Deng W., Zhang W.Q., Lv C.,. Temperature effect on microstructure and P-wave propagation in Linyi sandstone[J]. Applied Thermal Engineering, 2017,115:913–922.

[28] Peng J., Rong G., Cai M., Zhou C.B. A model for characterizing crack closure effect of rocks[J]. Engineering Geology, 2015,189:48–57.

[29] Zhang F., Zhao J., Hu D., Skoczylas F., Shao J.F., Laboratory Investigation on Physical and Mechanical Properties of Granite After Heating and Water-Cooling Treatment[J]. Rock Mechanics and Rock Engineering, 2018,51(3):677–694.

[30] Yu Y., Questioning the validity of the Brazilian Test for Determining tensile Strength of rocks[J]. Chinese Journal of Rock Mechanics and Engineering,2005(07):1150–1157.

[31] Li D.Y., Wong L.N.Y. The Brazilian Disc Test for Rock Mechanics Applications: Review and New Insights[J]. Rock Mechanics and Rock Engineering, 2013,46(2):269–287.

[32] Markides C.F., Kourkoulis S.K., The Stress Field in a Standardized Brazilian Disc: The Influence of the Loading Type Acting on the Actual Contact Length[J]. Rock Mechanics and Rock Engineering, 2012,45(2):145–158.

[33] Erarslan N., Williams D. J., Experimental, numerical and analytical studies on tensile strength of rocks[J]. International Journal of Rock Mechanics and Mining Sciences, 2012,49:21–30. doi: 10.1016/j.ijrmms.2011.11.007

[34] Chunan Tang, Wancheng Zhu, Shuhong Wang, Qinglei Yu. Numerical investigation on rock failure process induced by thermal stress. Chinese Journal of Rock Mechanics and Engineering, 2006, 25(10): 2071–2078. (in Chinese).

[35] Мусхелишьили Н.И.,Several, Basic Problems of Mathematical Elasticity [M]. Beijing: Science Press, 1958.

