# Supplementary figures and images for: Macro/Microfracture evolution and instability behaviors of high-temperature granite under water-cooling subjected to Brazilian splitting test using the DIC technique (part 2 of 2)
Source: PLoS One. 2023 Nov 29;18(11):e0294258. doi: 10.1371/journal.pone.0294258 (PMC10686506; doi:10.1371/journal.pone.0294258)

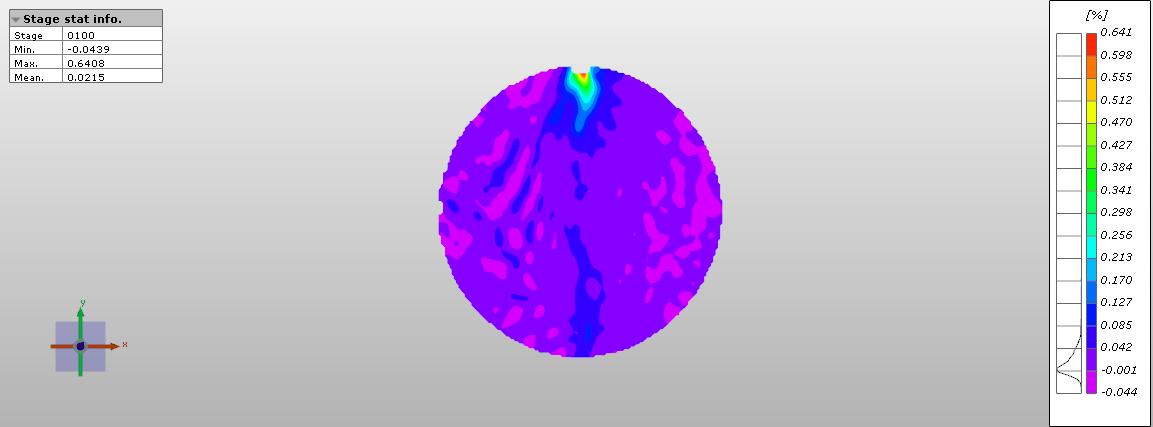

Supplement: S2 Data — (ZIP) [file pone.0294258.s002.zip › SNAPSERIES003/p0100.bmp]

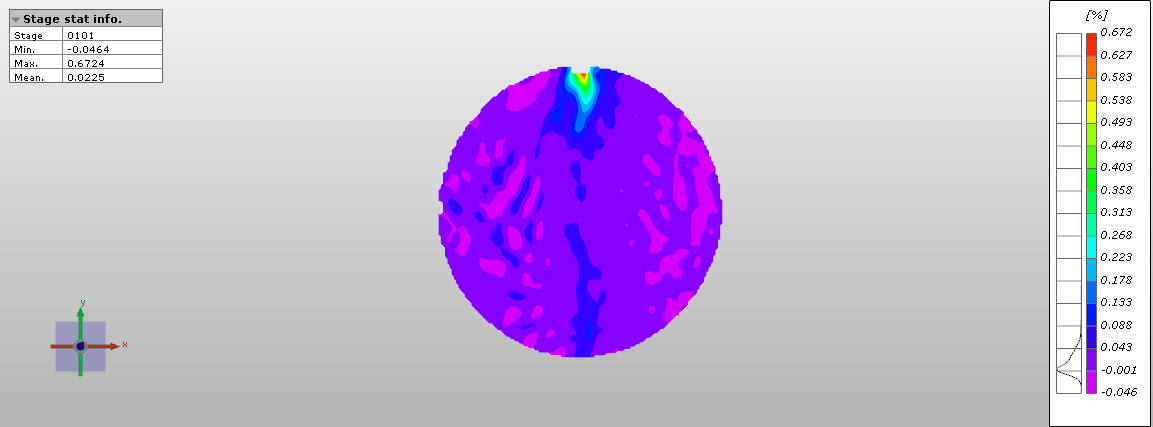

Supplement: S2 Data — (ZIP) [file pone.0294258.s002.zip › SNAPSERIES003/p0101.bmp]

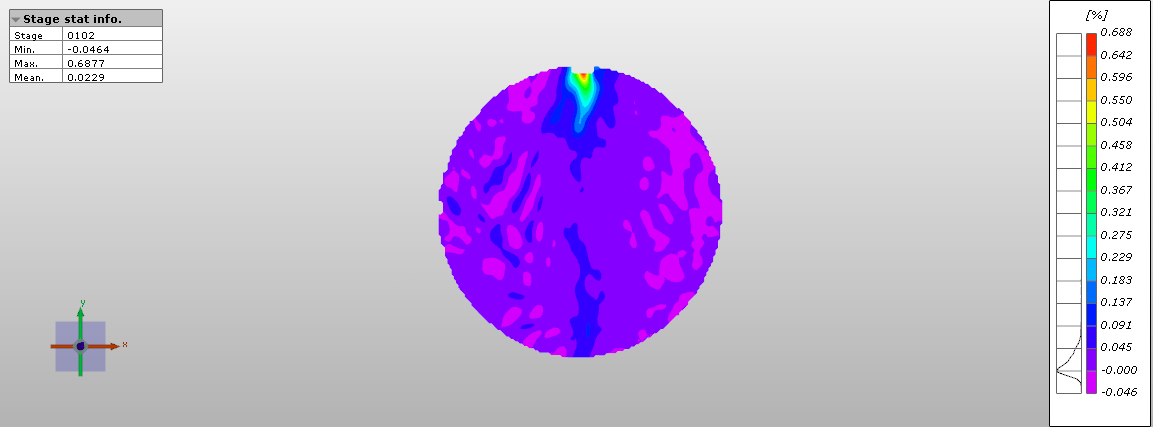

Supplement: S2 Data — (ZIP) [file pone.0294258.s002.zip › SNAPSERIES003/p0102.bmp]

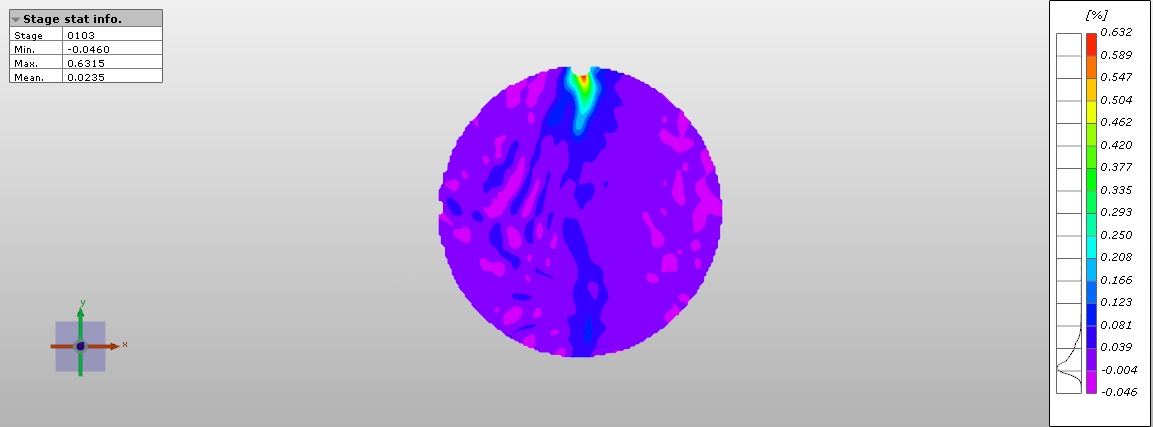

Supplement: S2 Data — (ZIP) [file pone.0294258.s002.zip › SNAPSERIES003/p0103.bmp]

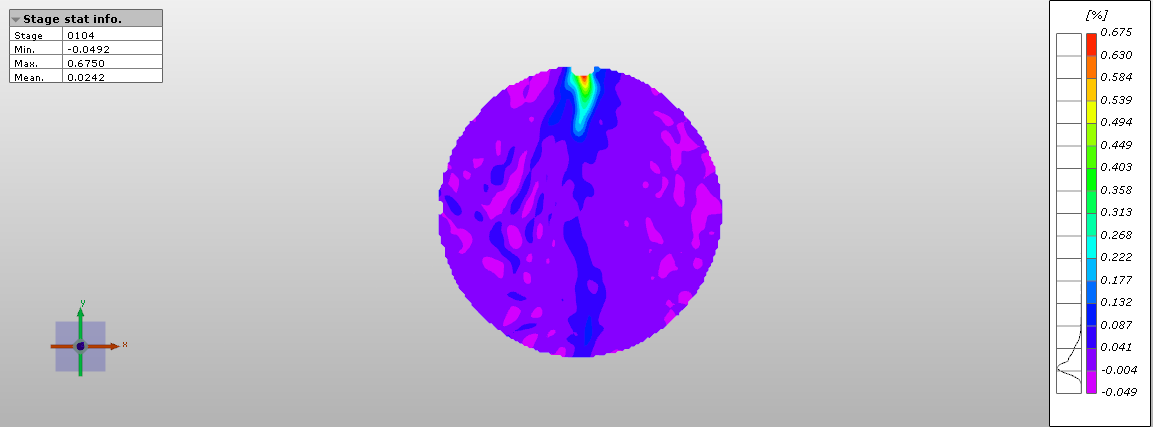

Supplement: S2 Data — (ZIP) [file pone.0294258.s002.zip › SNAPSERIES003/p0104.bmp]

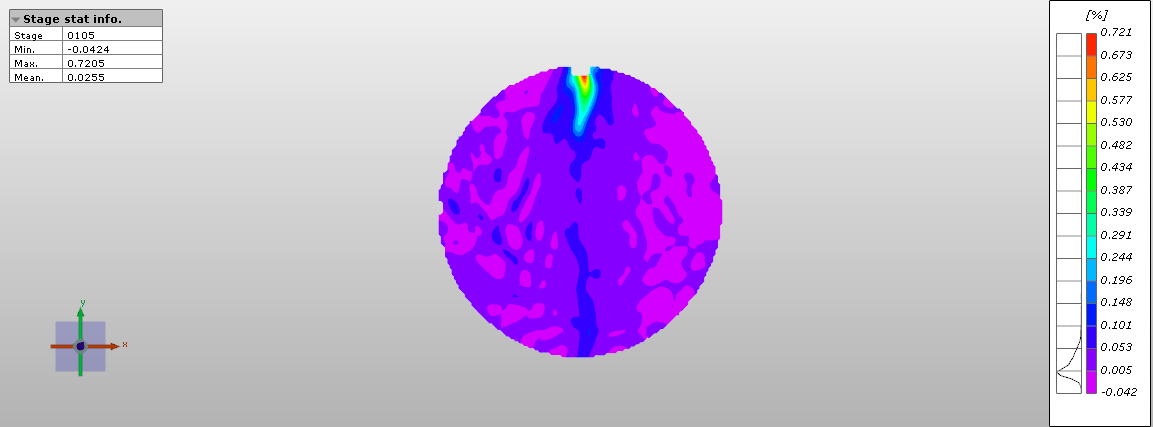

Supplement: S2 Data — (ZIP) [file pone.0294258.s002.zip › SNAPSERIES003/p0105.bmp]

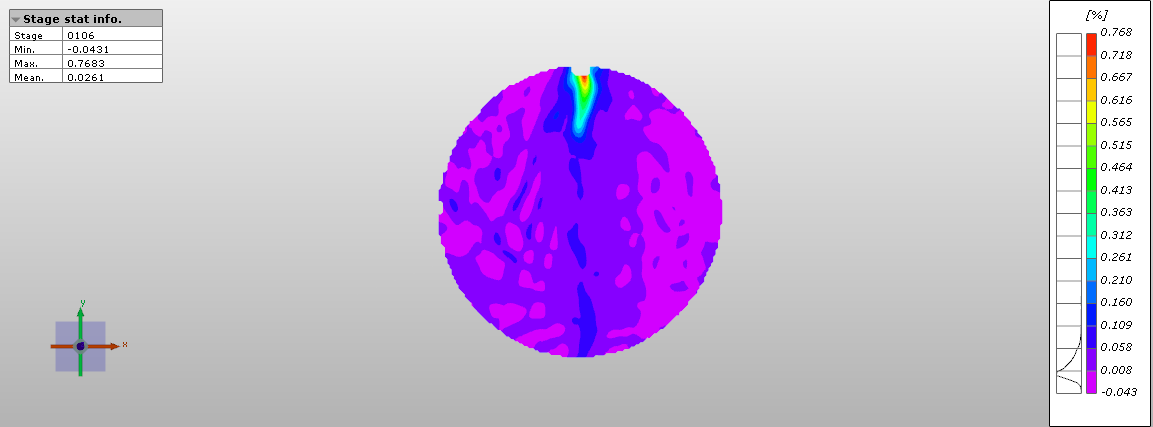

Supplement: S2 Data — (ZIP) [file pone.0294258.s002.zip › SNAPSERIES003/p0106.bmp]

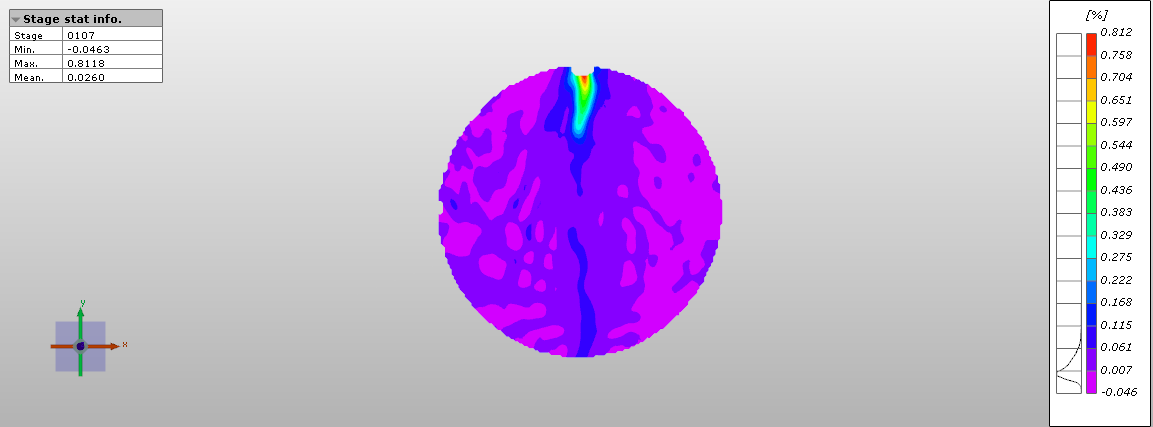

Supplement: S2 Data — (ZIP) [file pone.0294258.s002.zip › SNAPSERIES003/p0107.bmp]

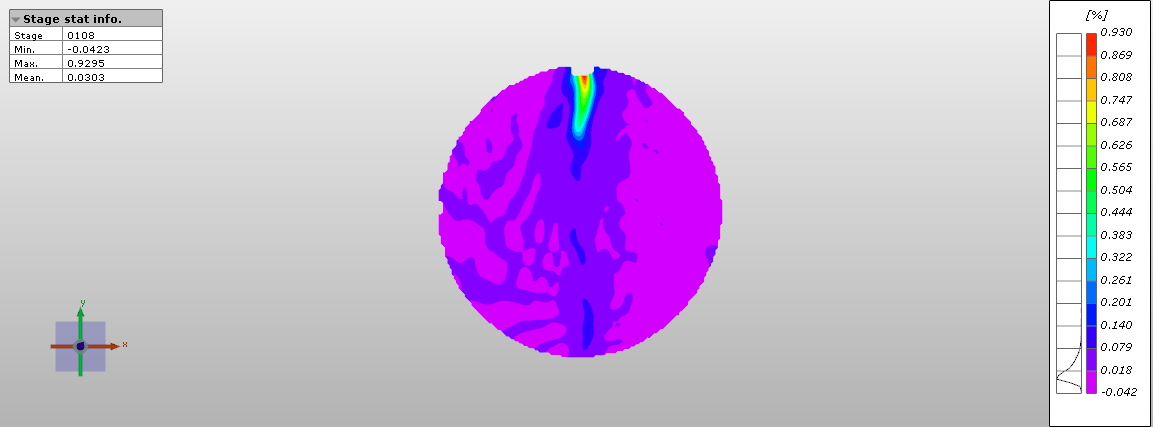

Supplement: S2 Data — (ZIP) [file pone.0294258.s002.zip › SNAPSERIES003/p0108.bmp]

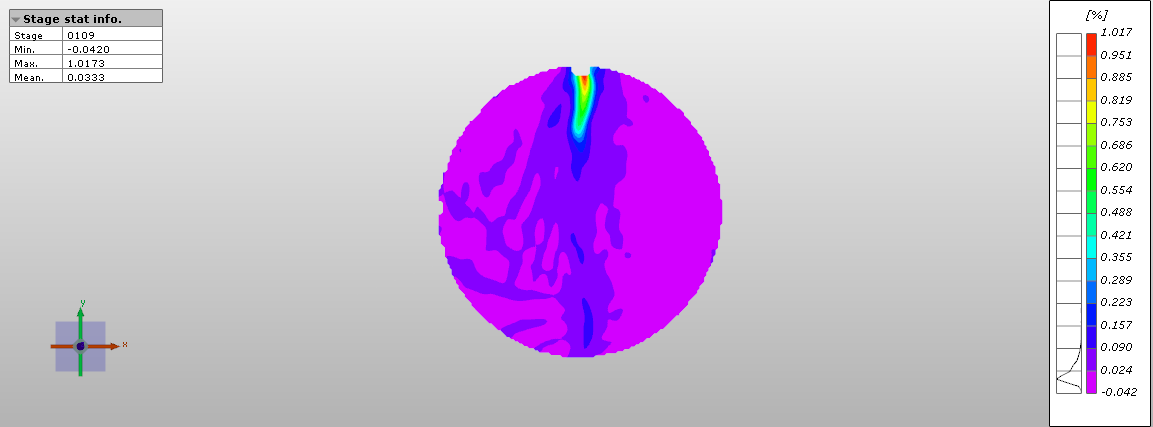

Supplement: S2 Data — (ZIP) [file pone.0294258.s002.zip › SNAPSERIES003/p0109.bmp]

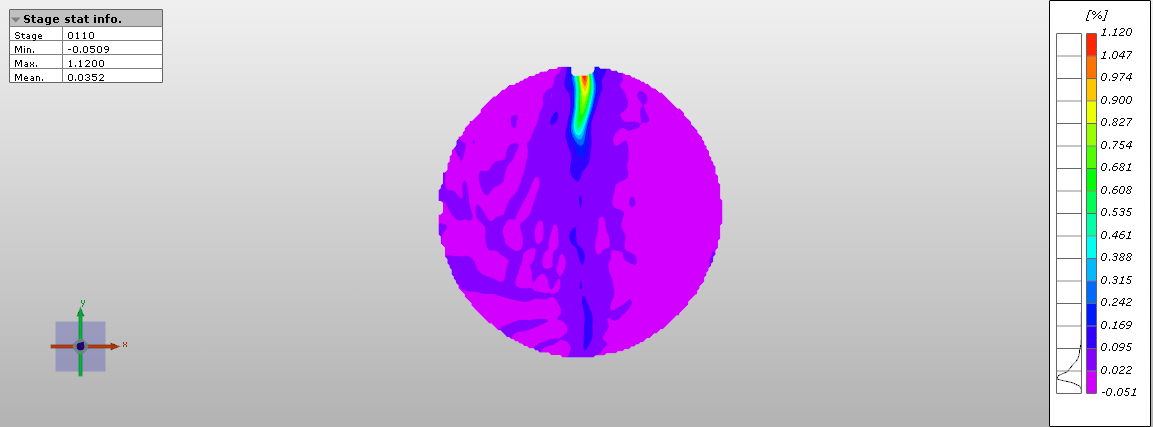

Supplement: S2 Data — (ZIP) [file pone.0294258.s002.zip › SNAPSERIES003/p0110.bmp]

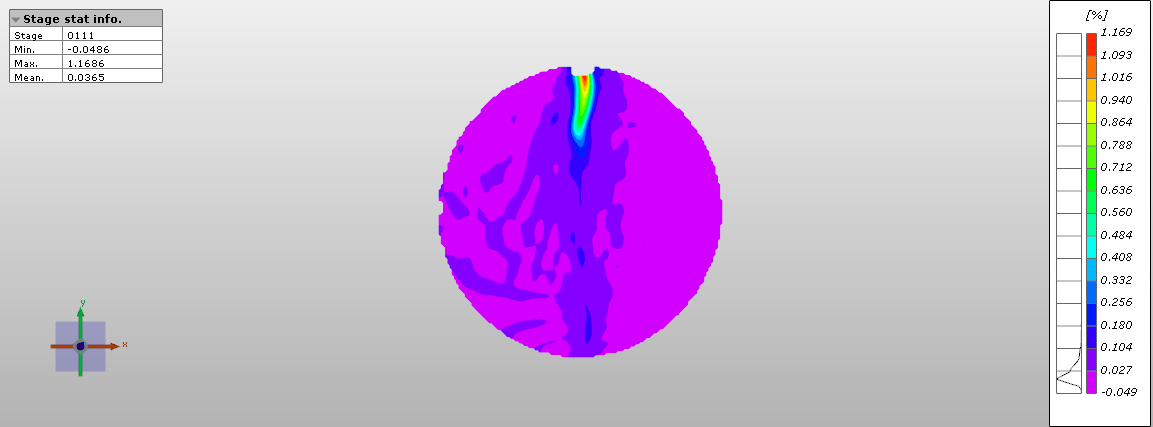

Supplement: S2 Data — (ZIP) [file pone.0294258.s002.zip › SNAPSERIES003/p0111.bmp]

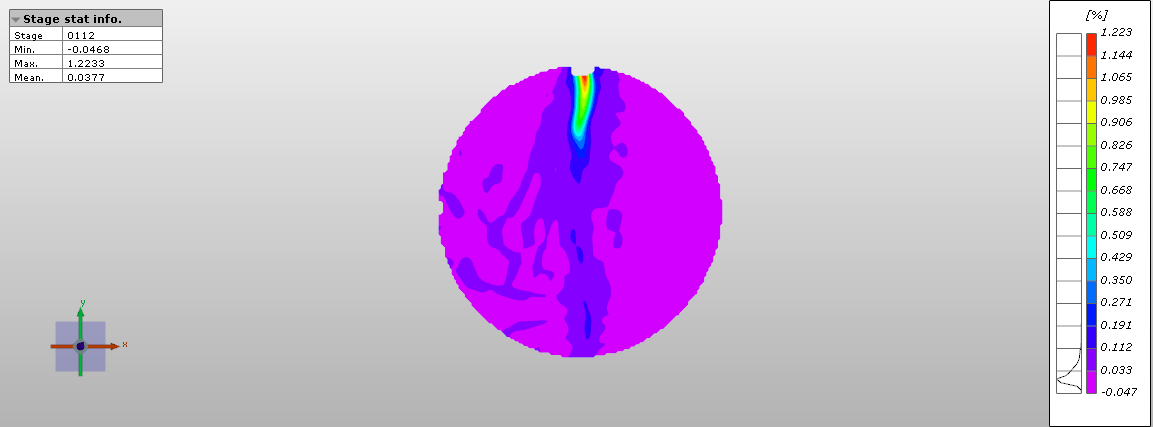

Supplement: S2 Data — (ZIP) [file pone.0294258.s002.zip › SNAPSERIES003/p0112.bmp]

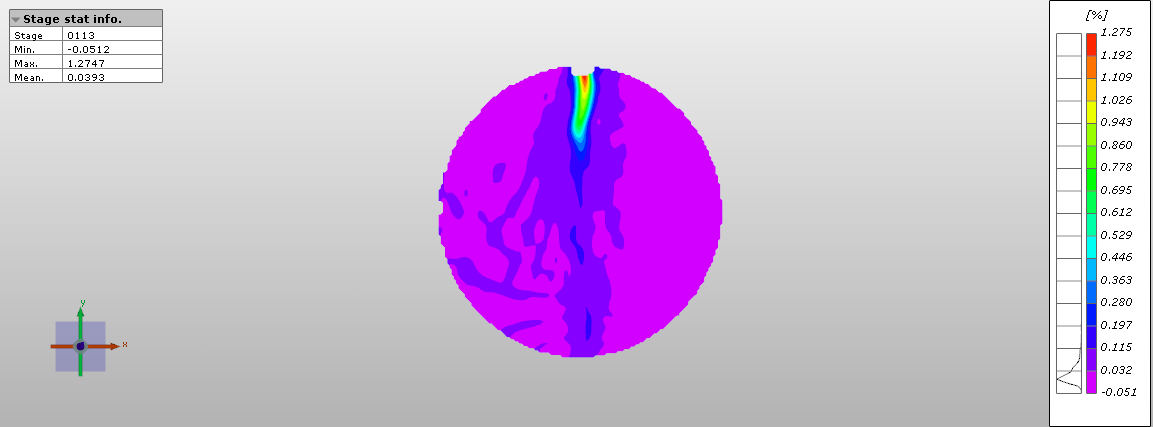

Supplement: S2 Data — (ZIP) [file pone.0294258.s002.zip › SNAPSERIES003/p0113.bmp]

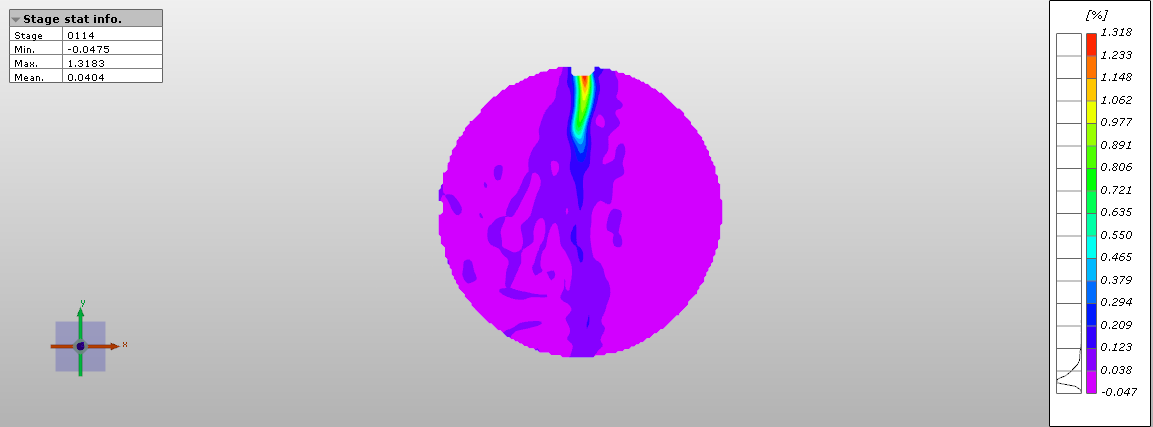

Supplement: S2 Data — (ZIP) [file pone.0294258.s002.zip › SNAPSERIES003/p0114.bmp]

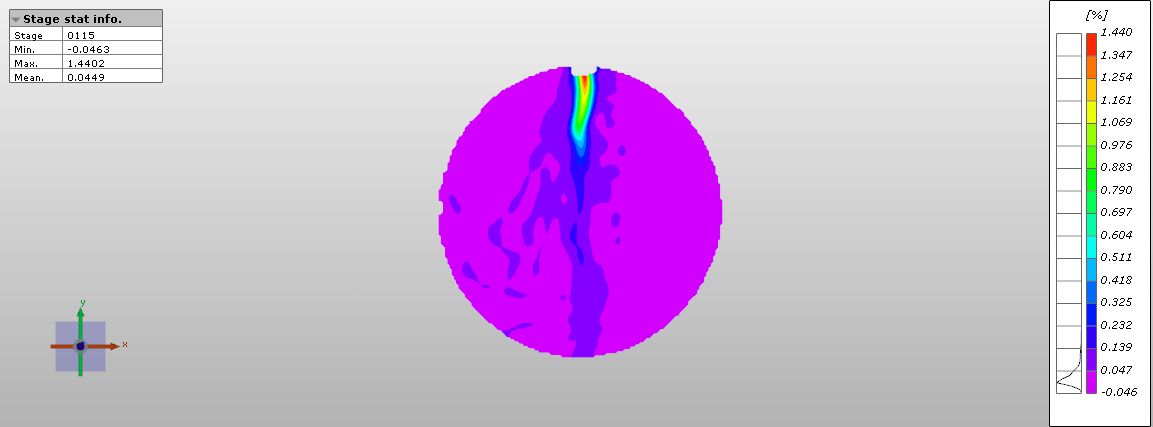

Supplement: S2 Data — (ZIP) [file pone.0294258.s002.zip › SNAPSERIES003/p0115.bmp]

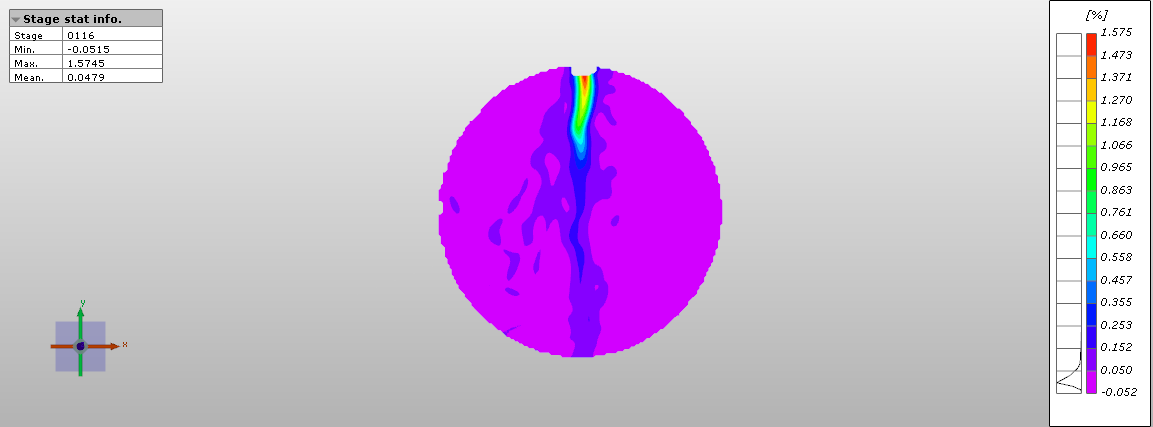

Supplement: S2 Data — (ZIP) [file pone.0294258.s002.zip › SNAPSERIES003/p0116.bmp]

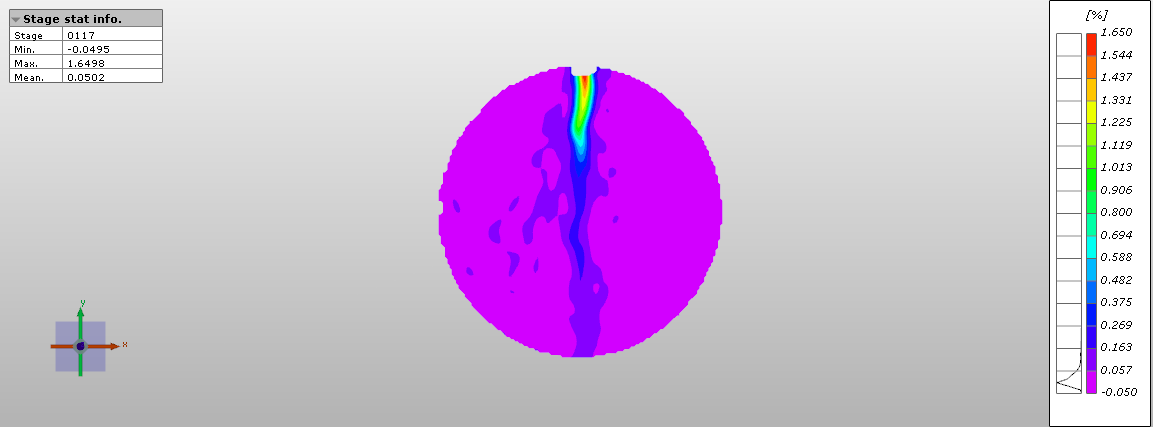

Supplement: S2 Data — (ZIP) [file pone.0294258.s002.zip › SNAPSERIES003/p0117.bmp]

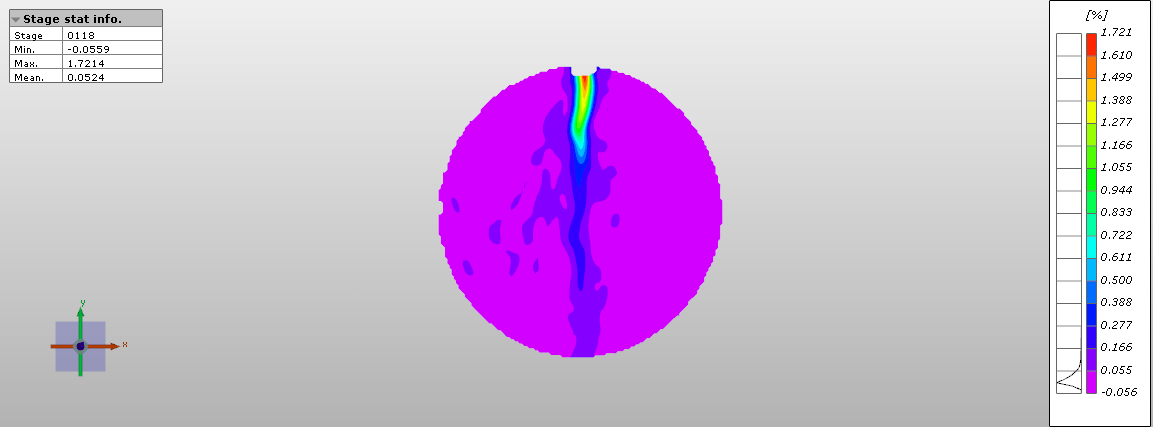

Supplement: S2 Data — (ZIP) [file pone.0294258.s002.zip › SNAPSERIES003/p0118.bmp]

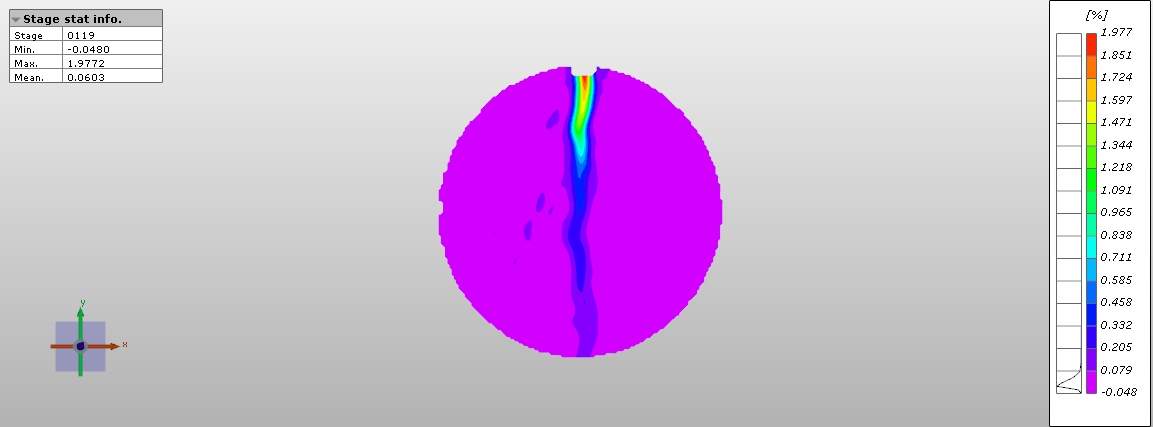

Supplement: S2 Data — (ZIP) [file pone.0294258.s002.zip › SNAPSERIES003/p0119.bmp]

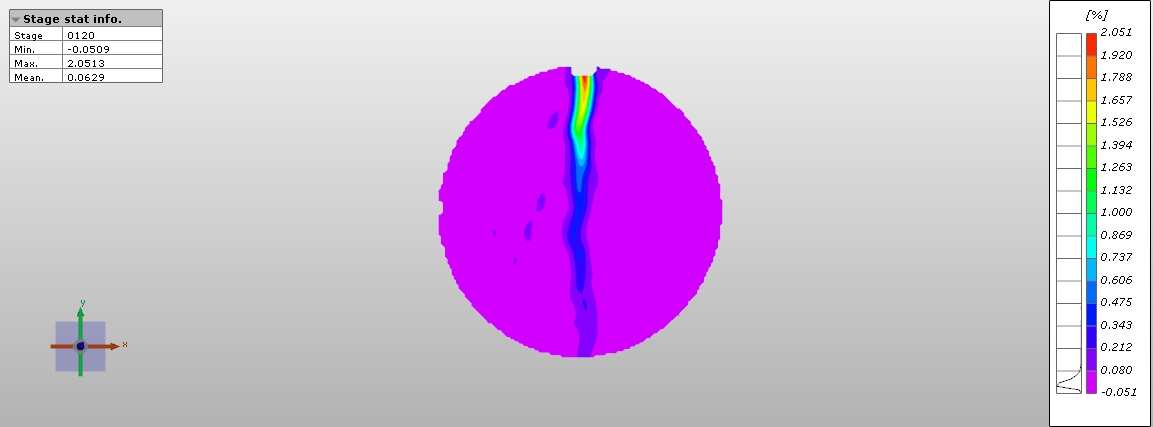

Supplement: S2 Data — (ZIP) [file pone.0294258.s002.zip › SNAPSERIES003/p0120.bmp]

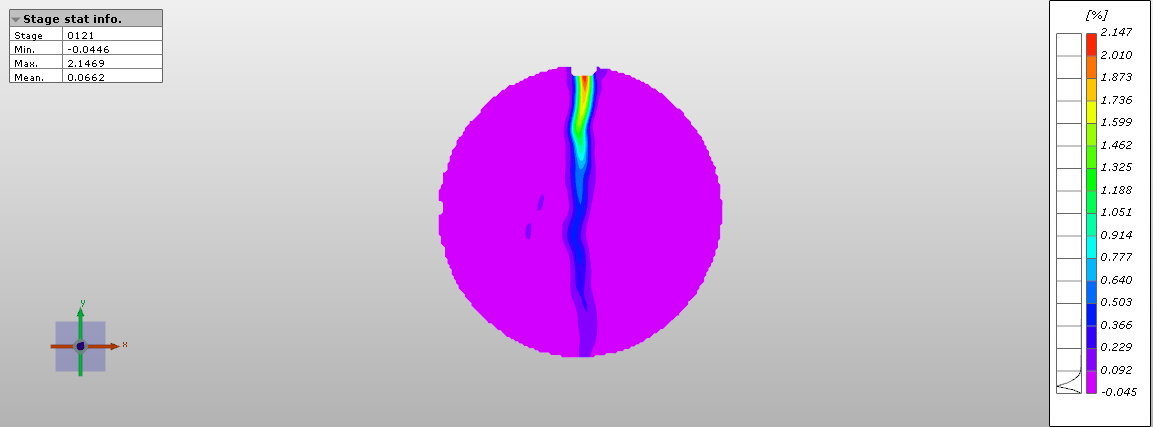

Supplement: S2 Data — (ZIP) [file pone.0294258.s002.zip › SNAPSERIES003/p0121.bmp]

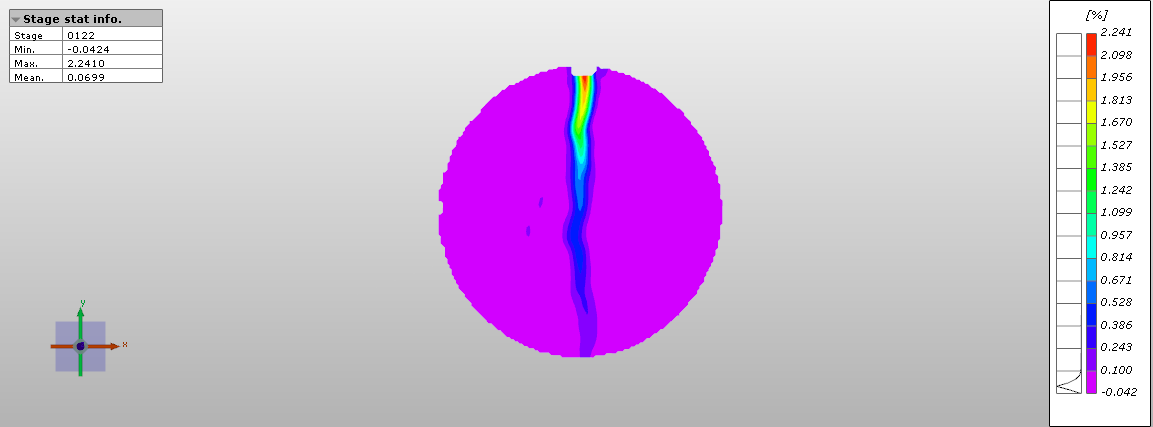

Supplement: S2 Data — (ZIP) [file pone.0294258.s002.zip › SNAPSERIES003/p0122.bmp]

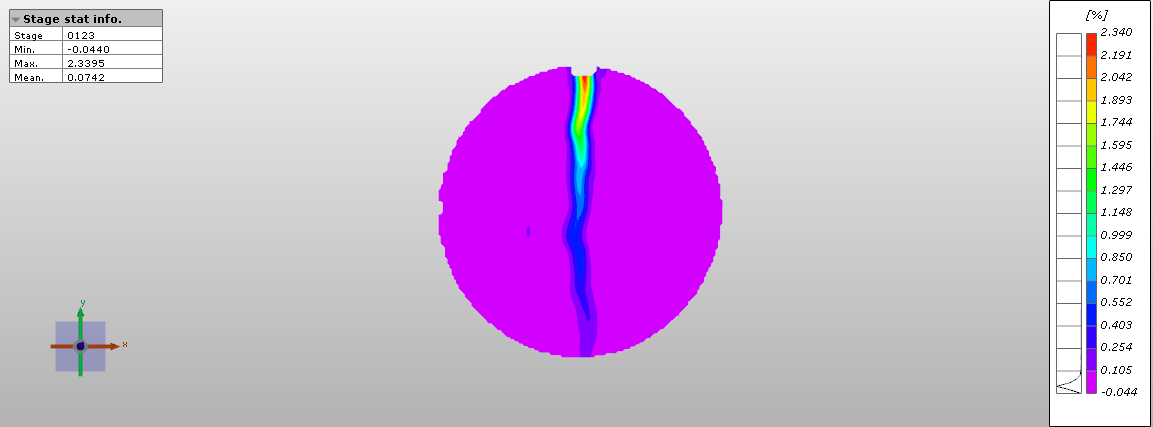

Supplement: S2 Data — (ZIP) [file pone.0294258.s002.zip › SNAPSERIES003/p0123.bmp]

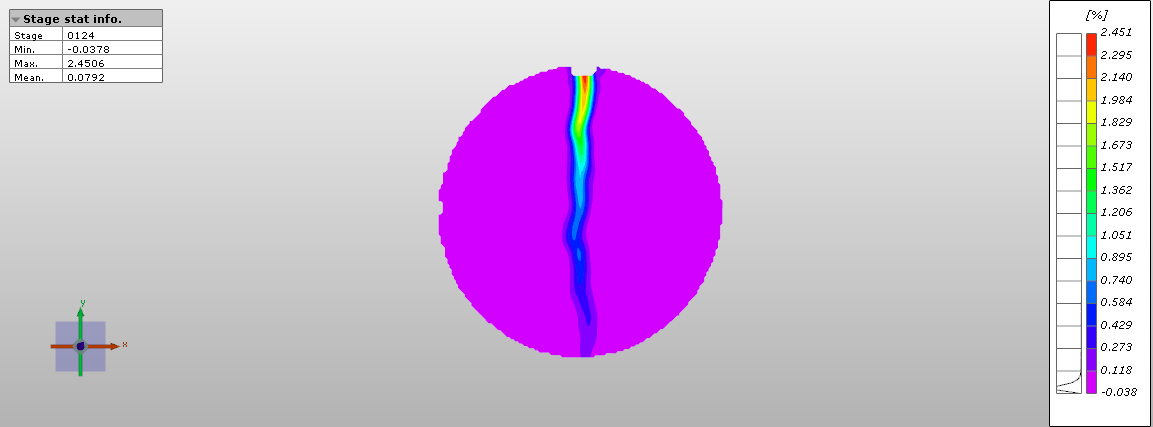

Supplement: S2 Data — (ZIP) [file pone.0294258.s002.zip › SNAPSERIES003/p0124.bmp]

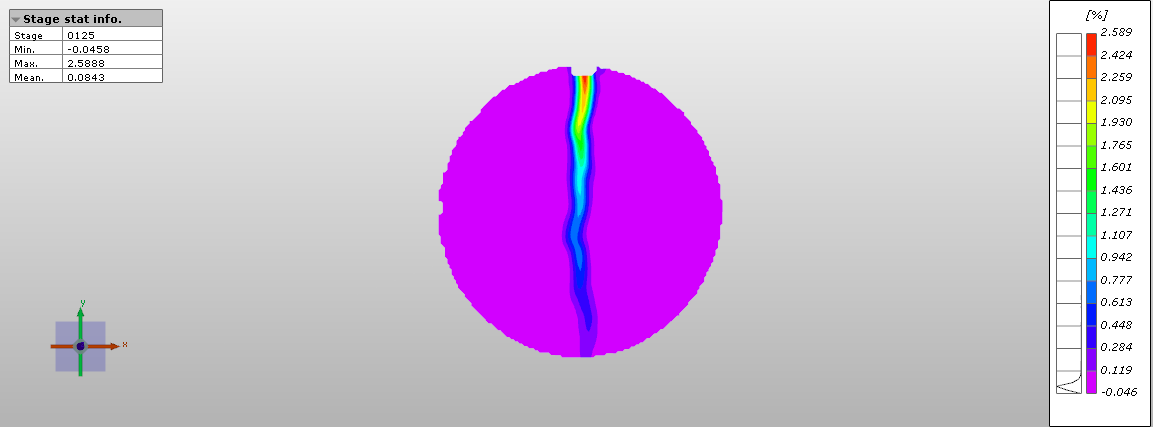

Supplement: S2 Data — (ZIP) [file pone.0294258.s002.zip › SNAPSERIES003/p0125.bmp]

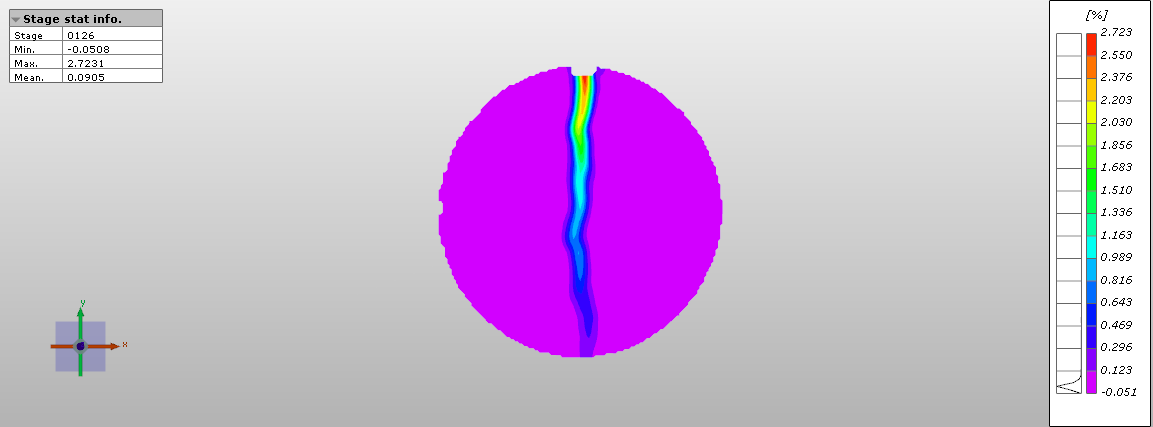

Supplement: S2 Data — (ZIP) [file pone.0294258.s002.zip › SNAPSERIES003/p0126.bmp]

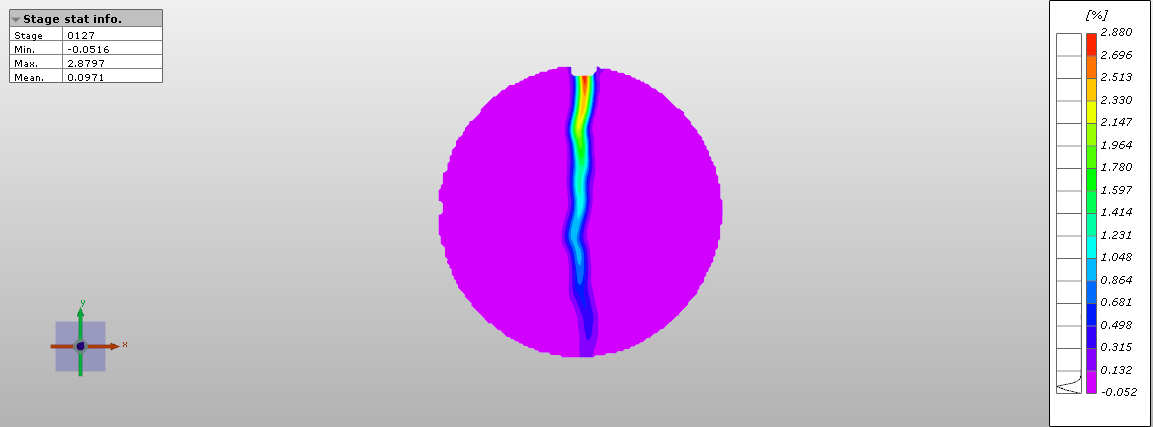

Supplement: S2 Data — (ZIP) [file pone.0294258.s002.zip › SNAPSERIES003/p0127.bmp]

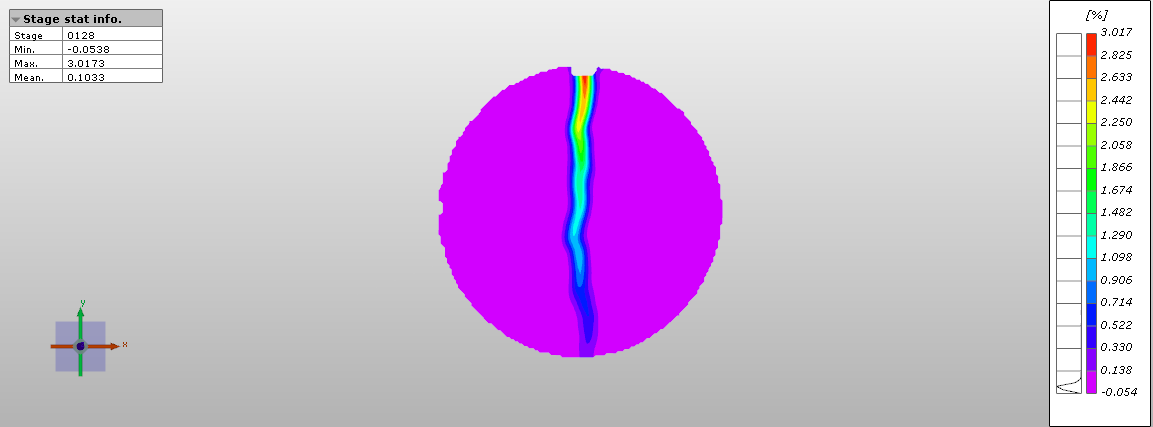

Supplement: S2 Data — (ZIP) [file pone.0294258.s002.zip › SNAPSERIES003/p0128.bmp]

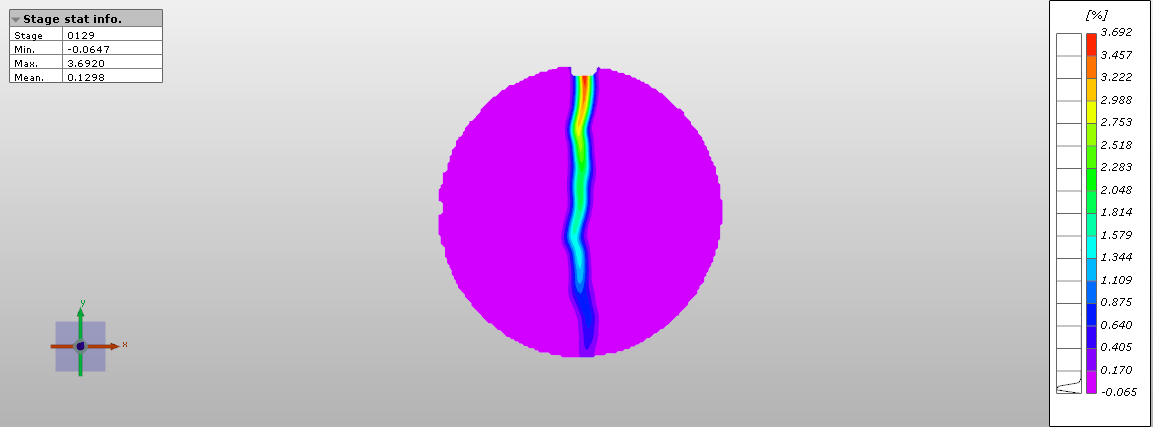

Supplement: S2 Data — (ZIP) [file pone.0294258.s002.zip › SNAPSERIES003/p0129.bmp]

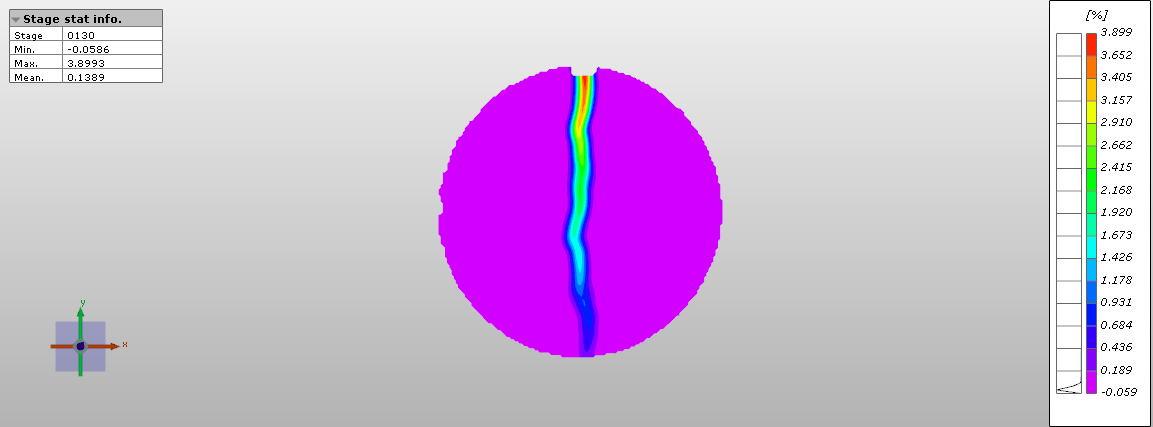

Supplement: S2 Data — (ZIP) [file pone.0294258.s002.zip › SNAPSERIES003/p0130.bmp]

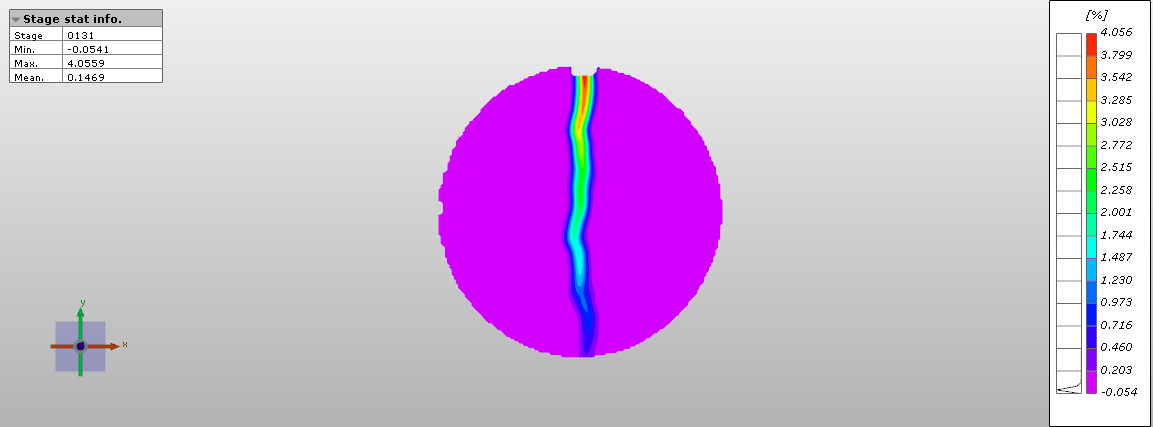

Supplement: S2 Data — (ZIP) [file pone.0294258.s002.zip › SNAPSERIES003/p0131.bmp]

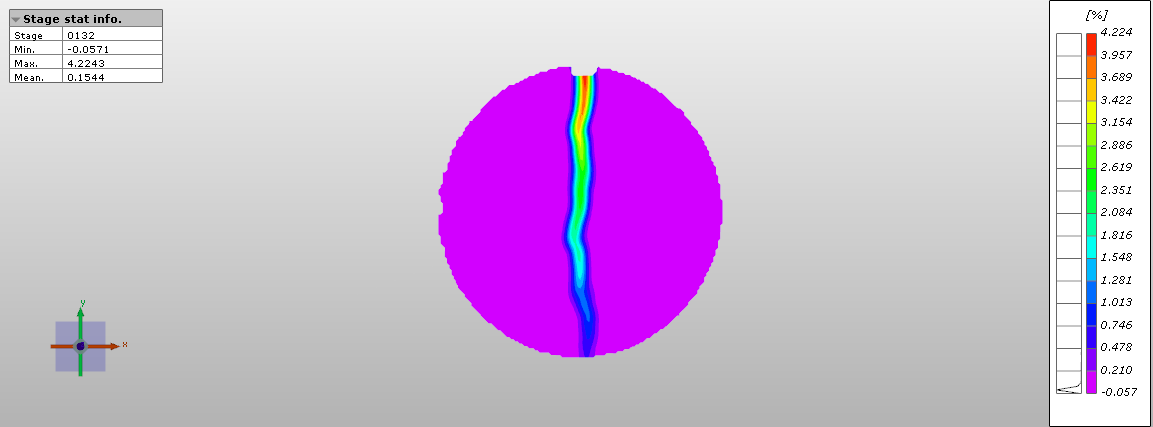

Supplement: S2 Data — (ZIP) [file pone.0294258.s002.zip › SNAPSERIES003/p0132.bmp]

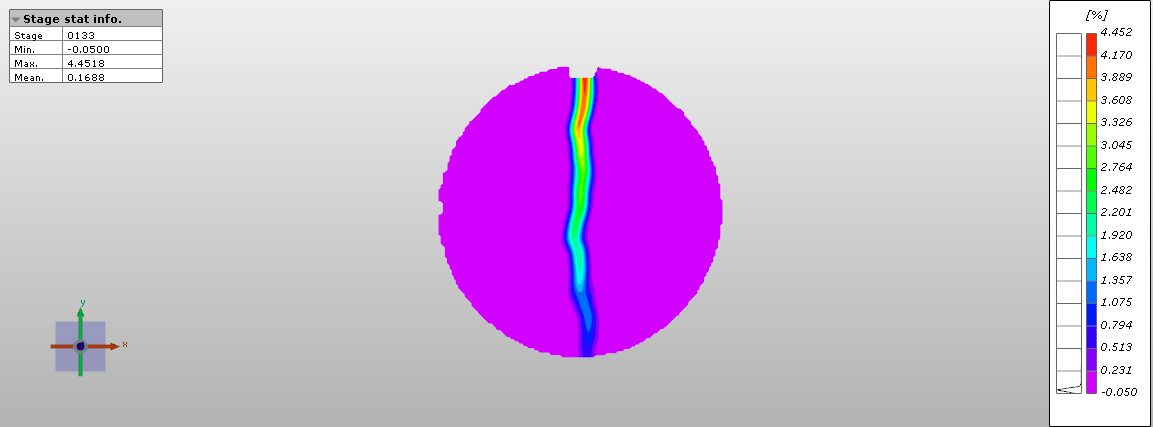

Supplement: S2 Data — (ZIP) [file pone.0294258.s002.zip › SNAPSERIES003/p0133.bmp]

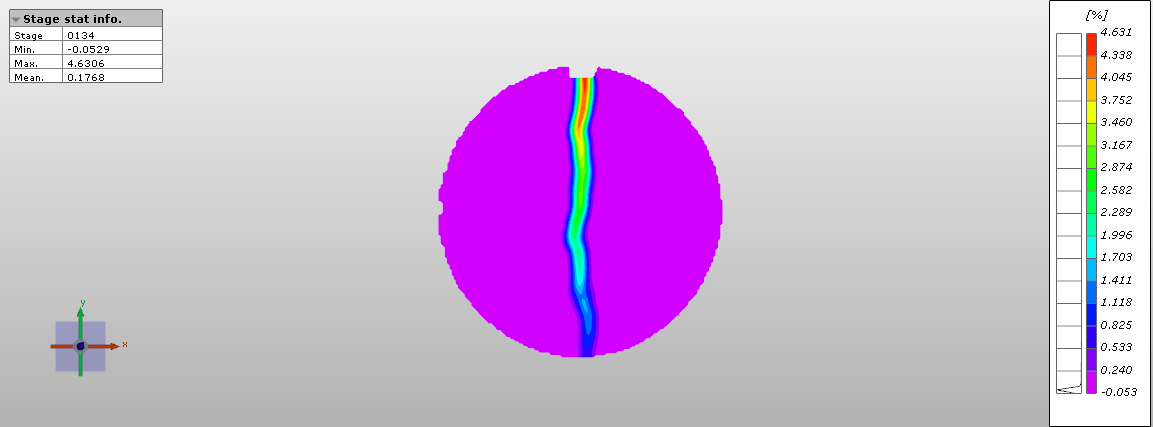

Supplement: S2 Data — (ZIP) [file pone.0294258.s002.zip › SNAPSERIES003/p0134.bmp]

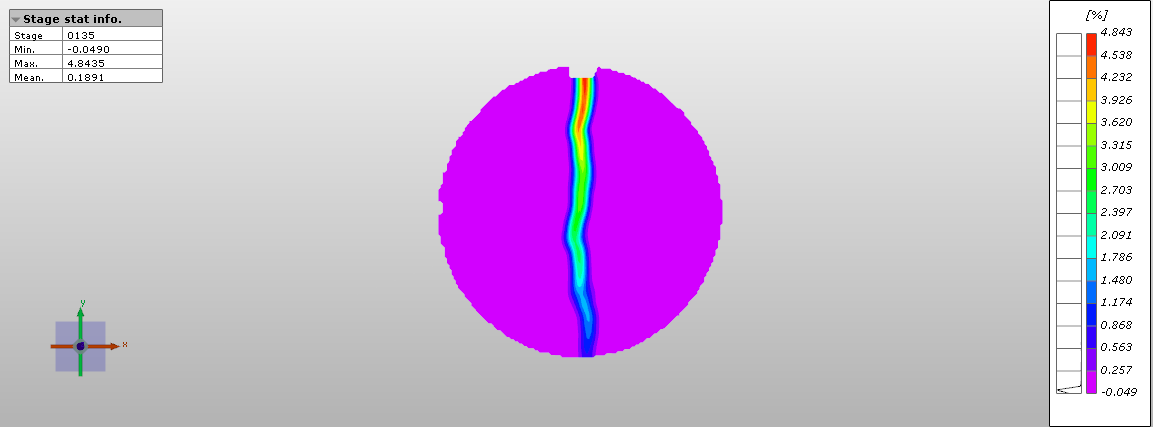

Supplement: S2 Data — (ZIP) [file pone.0294258.s002.zip › SNAPSERIES003/p0135.bmp]

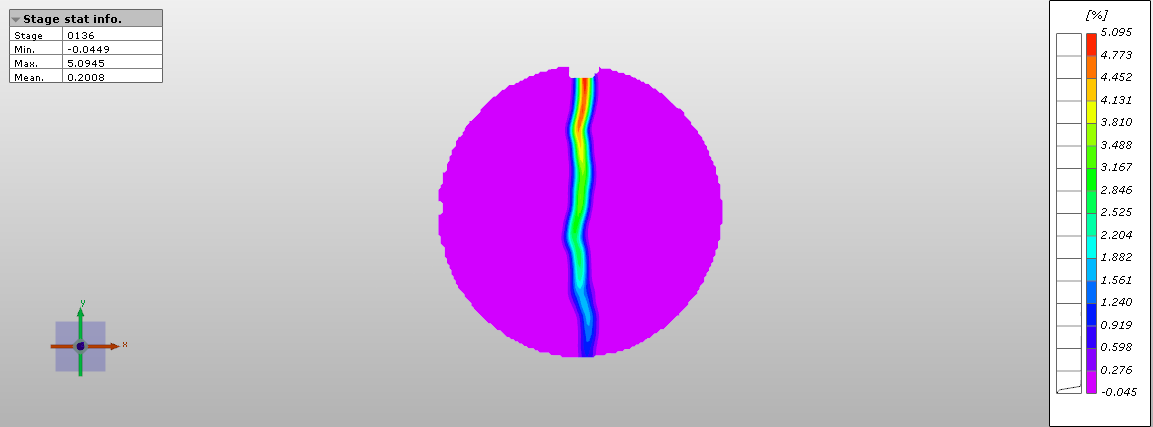

Supplement: S2 Data — (ZIP) [file pone.0294258.s002.zip › SNAPSERIES003/p0136.bmp]

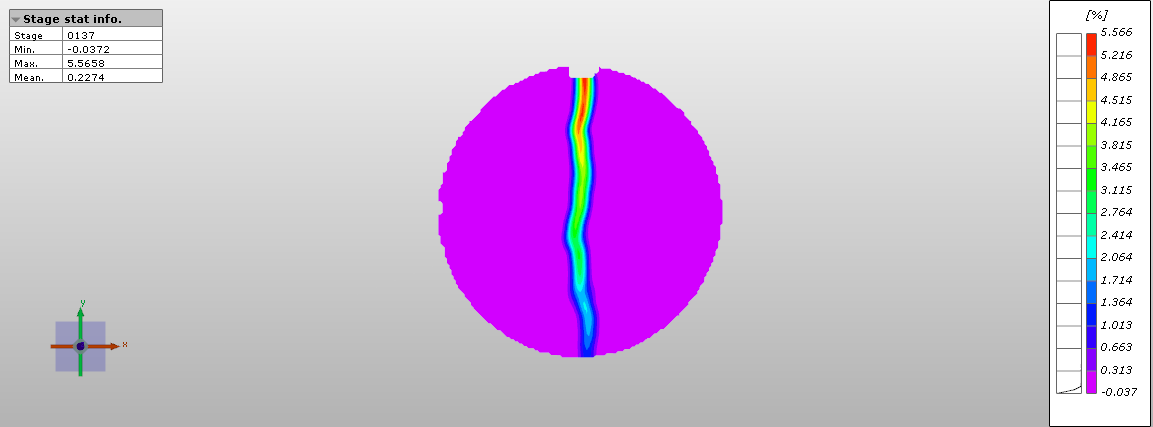

Supplement: S2 Data — (ZIP) [file pone.0294258.s002.zip › SNAPSERIES003/p0137.bmp]

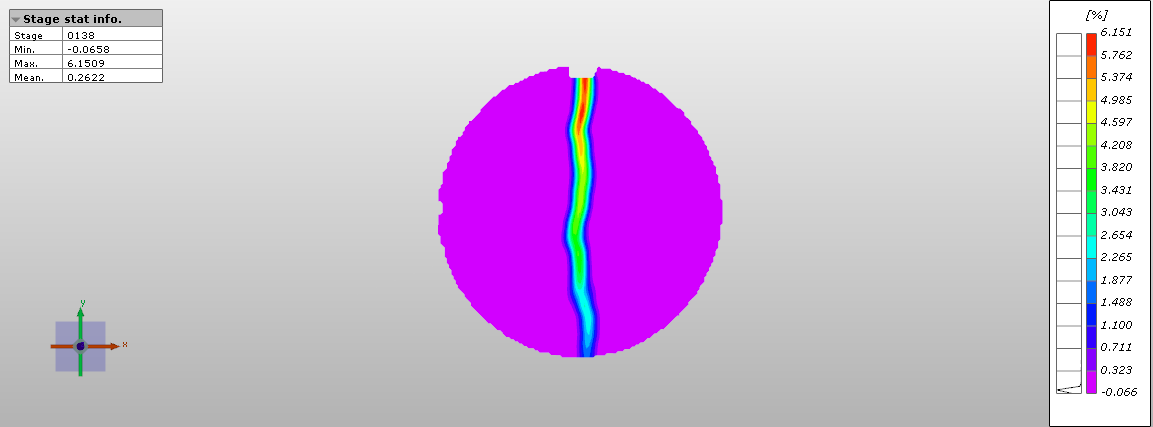

Supplement: S2 Data — (ZIP) [file pone.0294258.s002.zip › SNAPSERIES003/p0138.bmp]

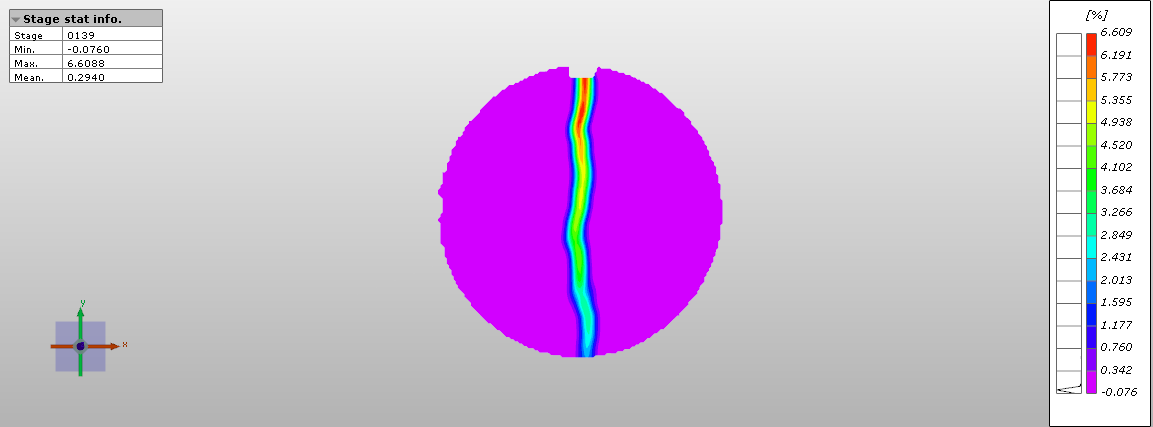

Supplement: S2 Data — (ZIP) [file pone.0294258.s002.zip › SNAPSERIES003/p0139.bmp]

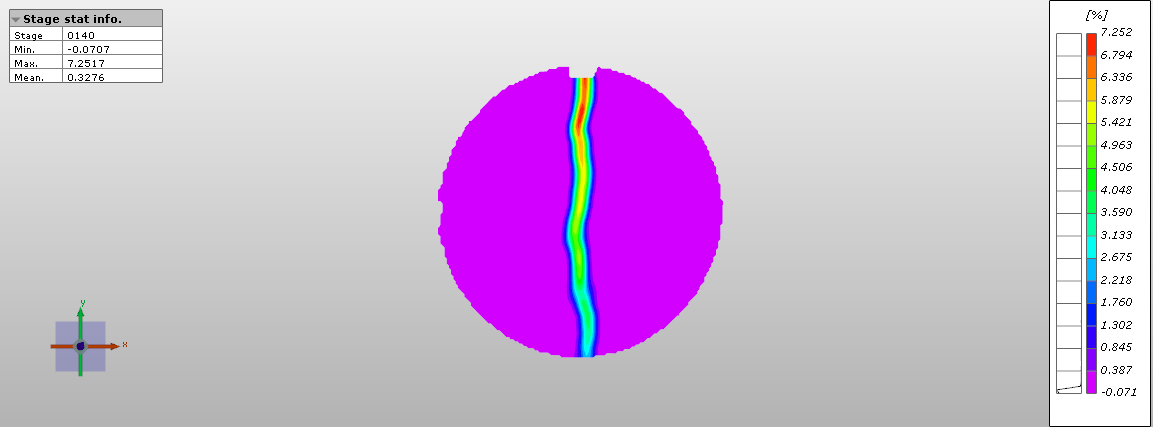

Supplement: S2 Data — (ZIP) [file pone.0294258.s002.zip › SNAPSERIES003/p0140.bmp]

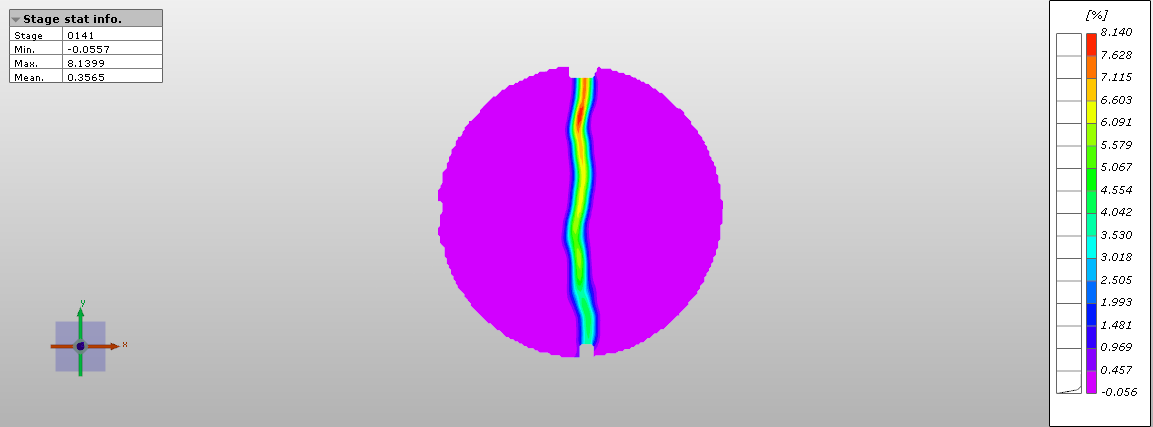

Supplement: S2 Data — (ZIP) [file pone.0294258.s002.zip › SNAPSERIES003/p0141.bmp]

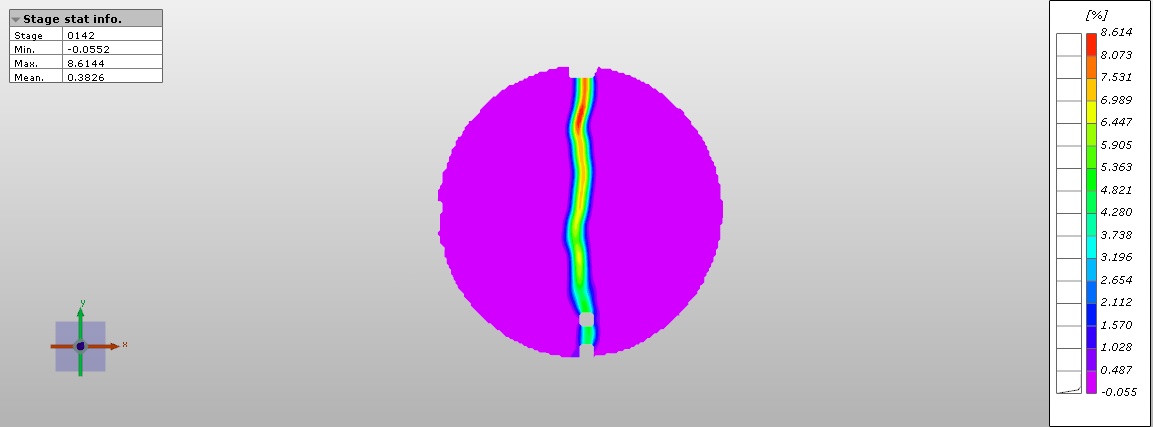

Supplement: S2 Data — (ZIP) [file pone.0294258.s002.zip › SNAPSERIES003/p0142.bmp]

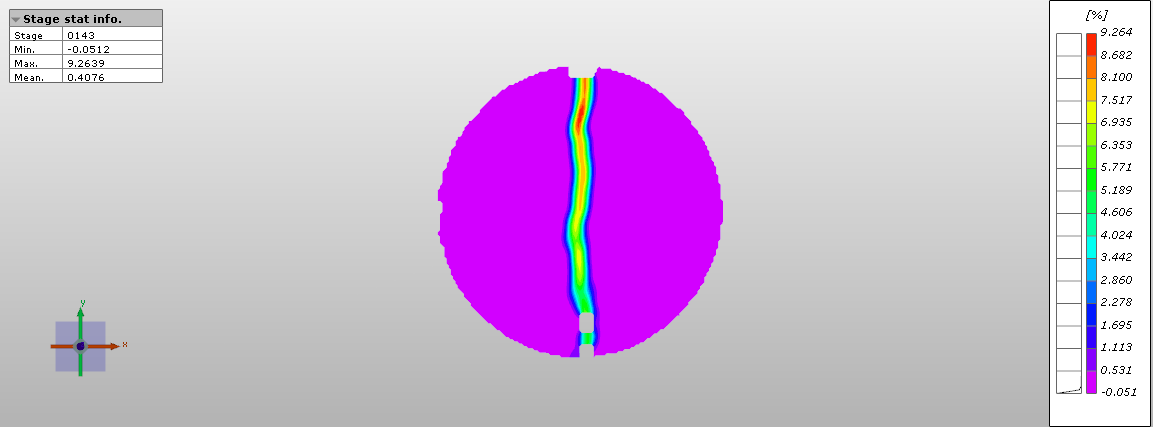

Supplement: S2 Data — (ZIP) [file pone.0294258.s002.zip › SNAPSERIES003/p0143.bmp]

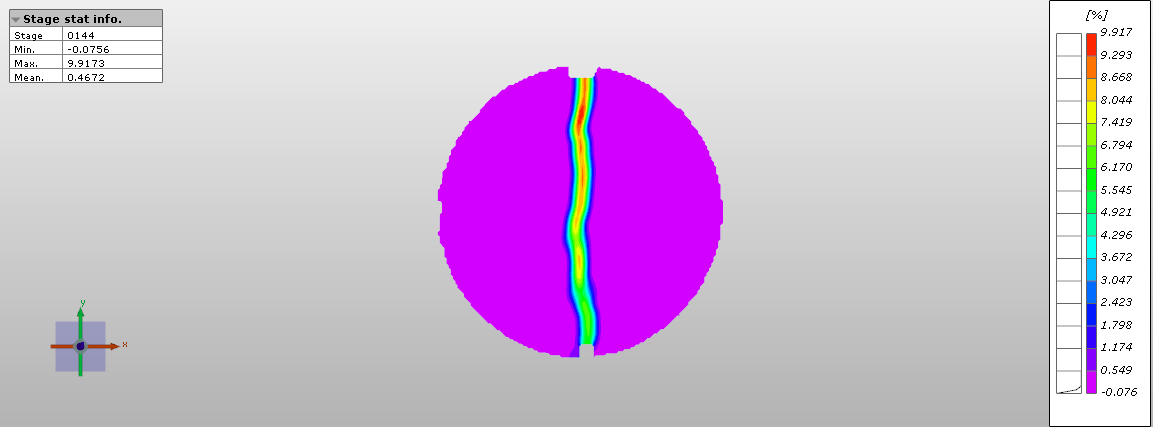

Supplement: S2 Data — (ZIP) [file pone.0294258.s002.zip › SNAPSERIES003/p0144.bmp]

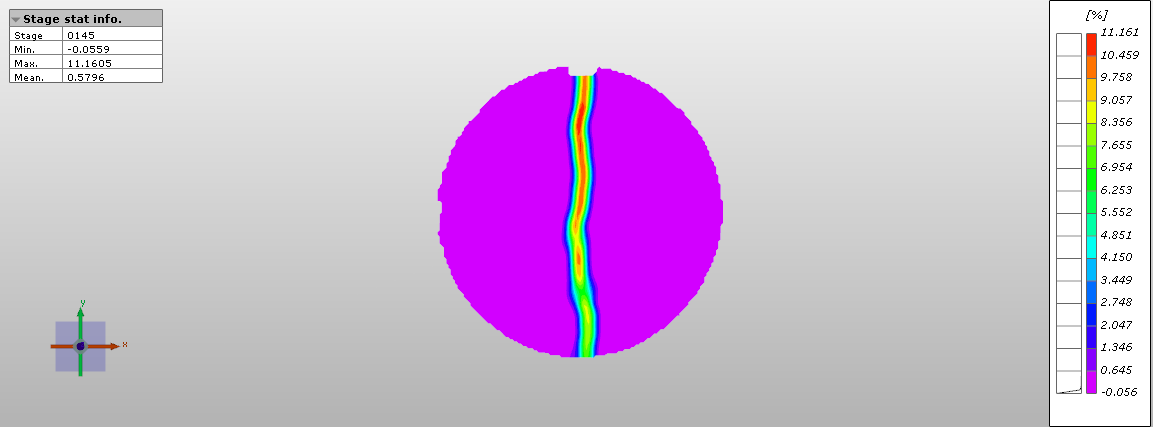

Supplement: S2 Data — (ZIP) [file pone.0294258.s002.zip › SNAPSERIES003/p0145.bmp]

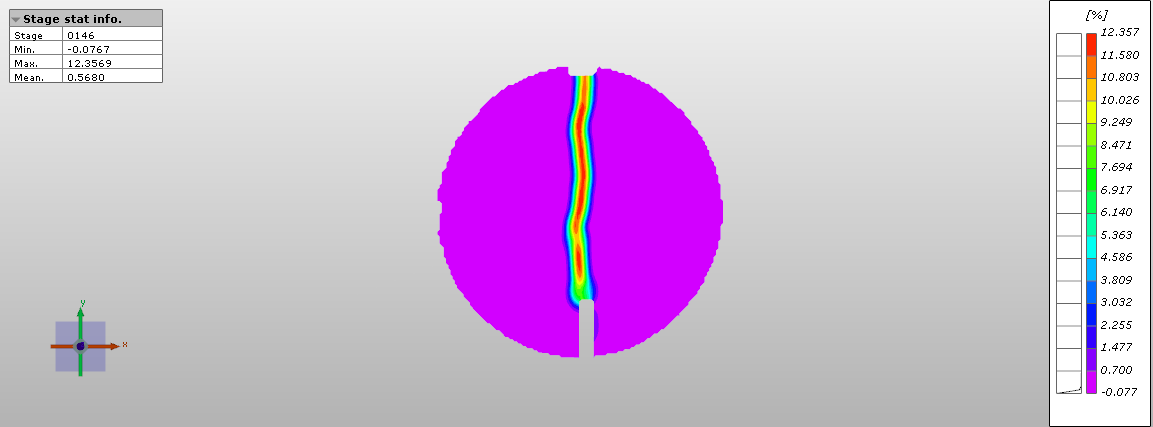

Supplement: S2 Data — (ZIP) [file pone.0294258.s002.zip › SNAPSERIES003/p0146.bmp]

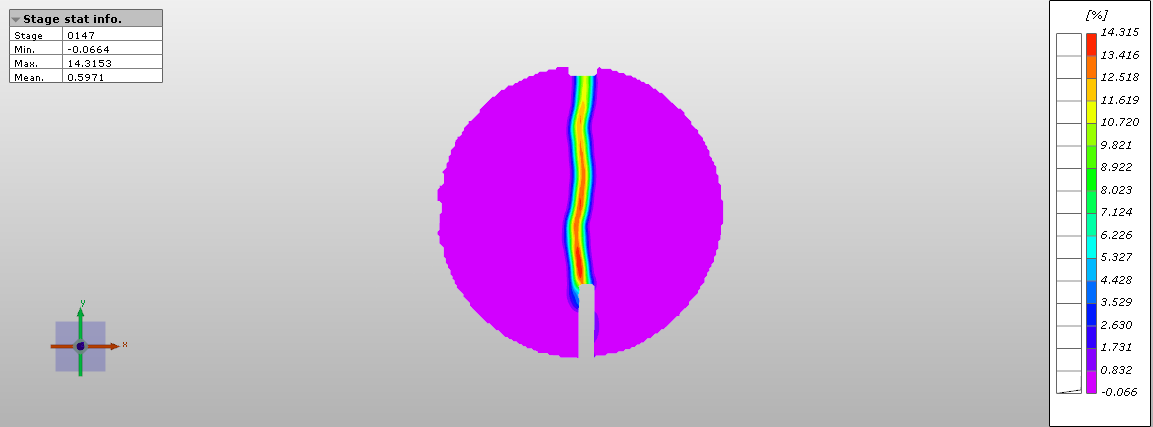

Supplement: S2 Data — (ZIP) [file pone.0294258.s002.zip › SNAPSERIES003/p0147.bmp]

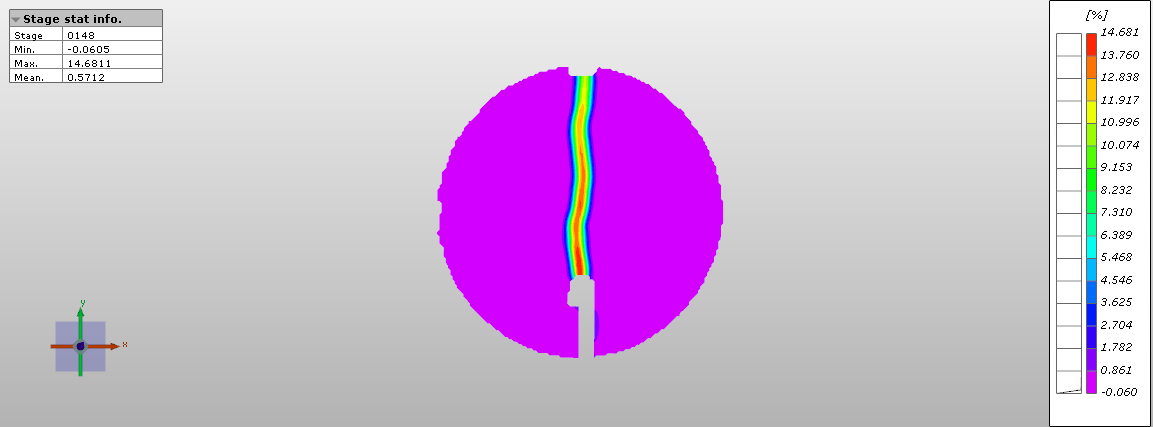

Supplement: S2 Data — (ZIP) [file pone.0294258.s002.zip › SNAPSERIES003/p0148.bmp]

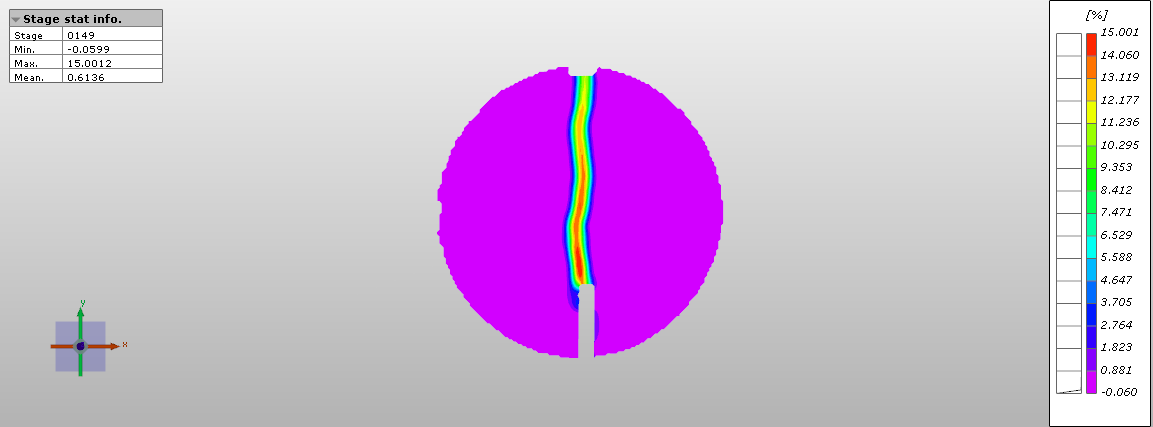

Supplement: S2 Data — (ZIP) [file pone.0294258.s002.zip › SNAPSERIES003/p0149.bmp]

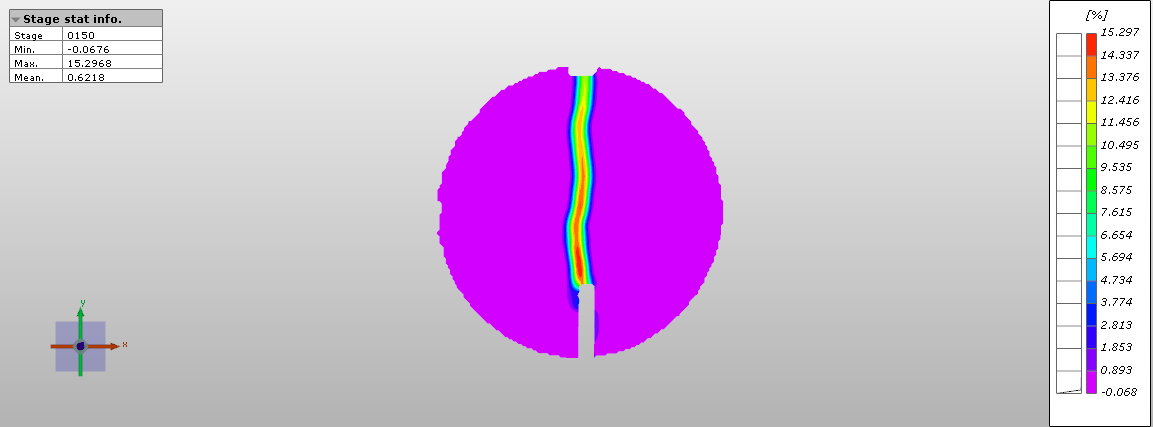

Supplement: S2 Data — (ZIP) [file pone.0294258.s002.zip › SNAPSERIES003/p0150.bmp]

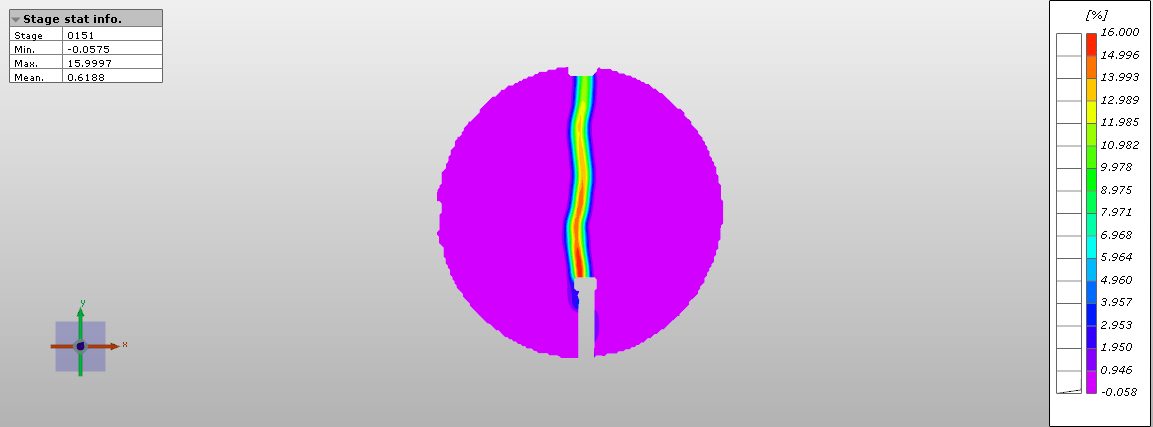

Supplement: S2 Data — (ZIP) [file pone.0294258.s002.zip › SNAPSERIES003/p0151.bmp]

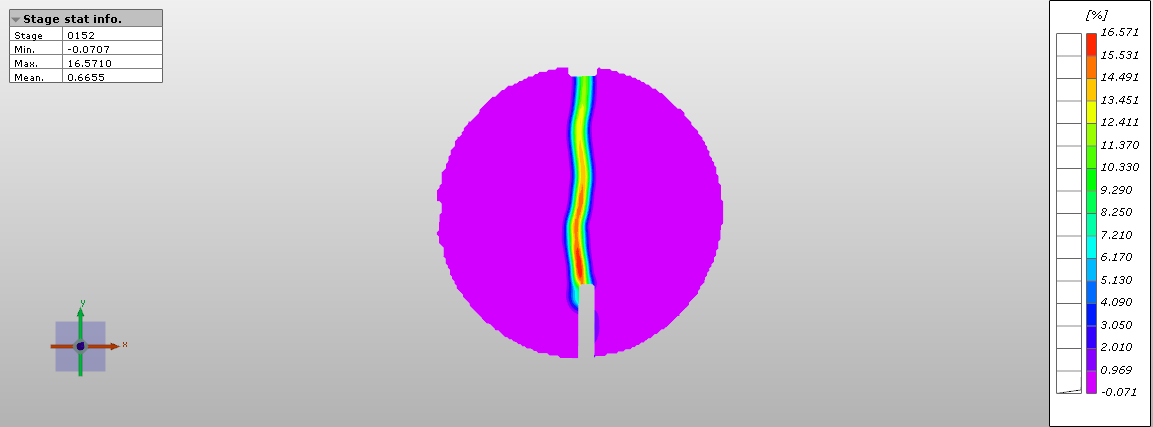

Supplement: S2 Data — (ZIP) [file pone.0294258.s002.zip › SNAPSERIES003/p0152.bmp]

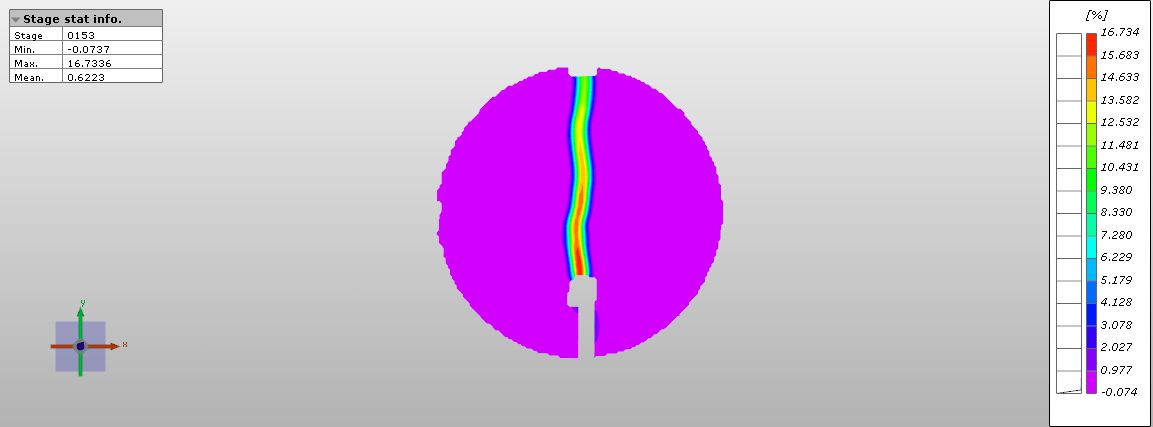

Supplement: S2 Data — (ZIP) [file pone.0294258.s002.zip › SNAPSERIES003/p0153.bmp]

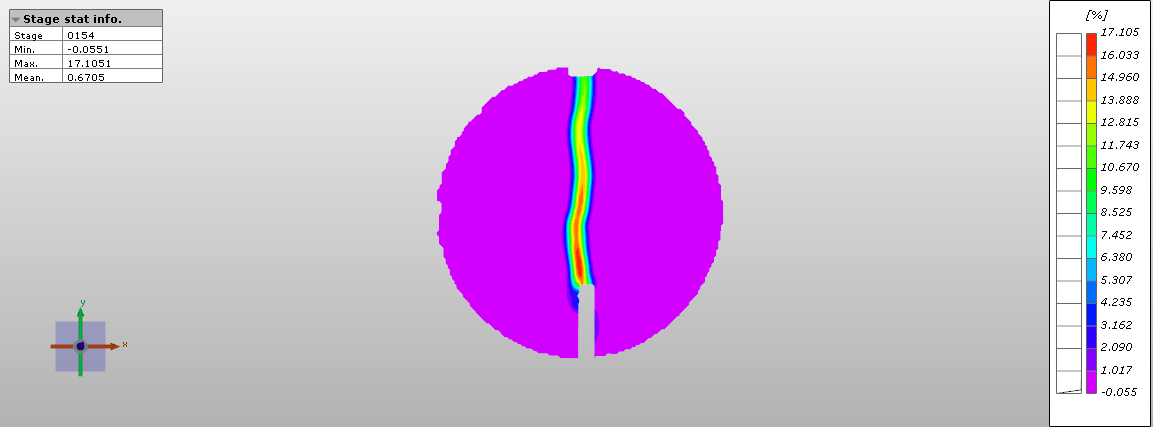

Supplement: S2 Data — (ZIP) [file pone.0294258.s002.zip › SNAPSERIES003/p0154.bmp]

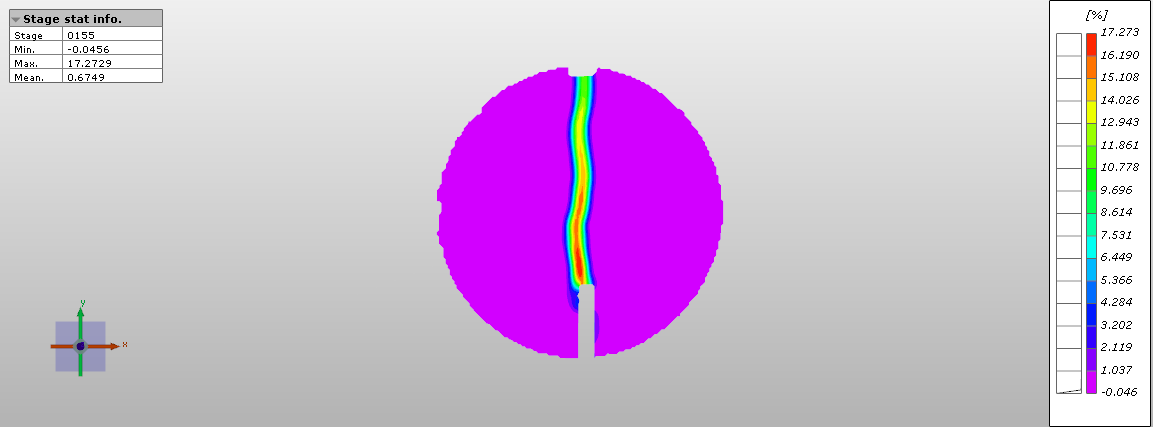

Supplement: S2 Data — (ZIP) [file pone.0294258.s002.zip › SNAPSERIES003/p0155.bmp]

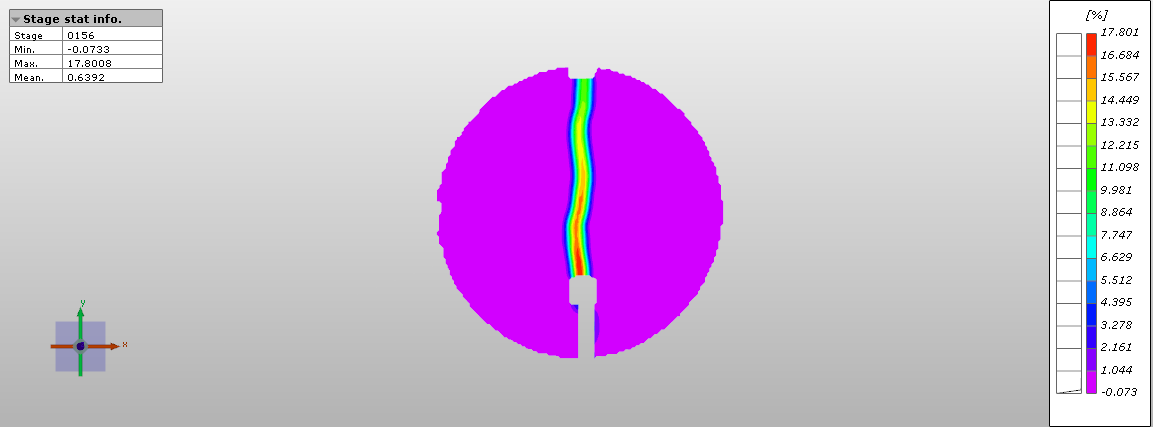

Supplement: S2 Data — (ZIP) [file pone.0294258.s002.zip › SNAPSERIES003/p0156.bmp]

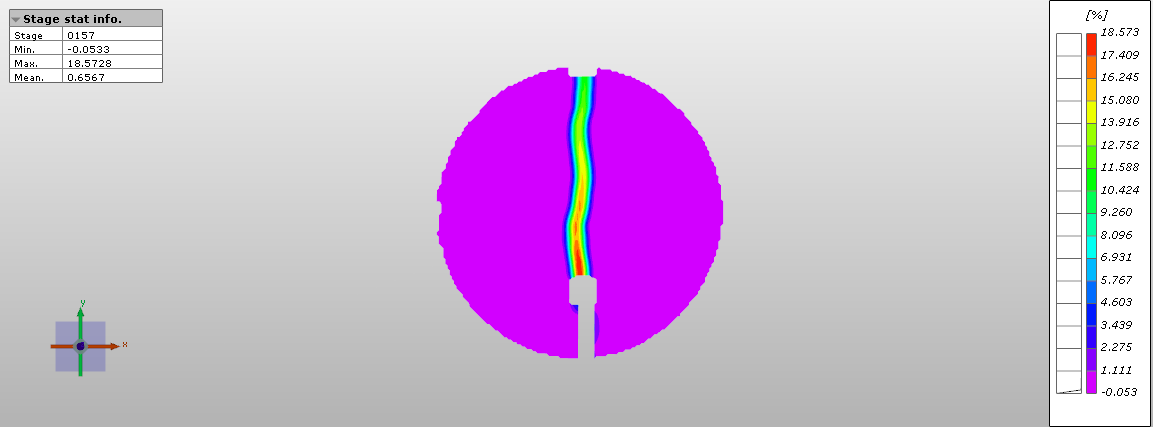

Supplement: S2 Data — (ZIP) [file pone.0294258.s002.zip › SNAPSERIES003/p0157.bmp]

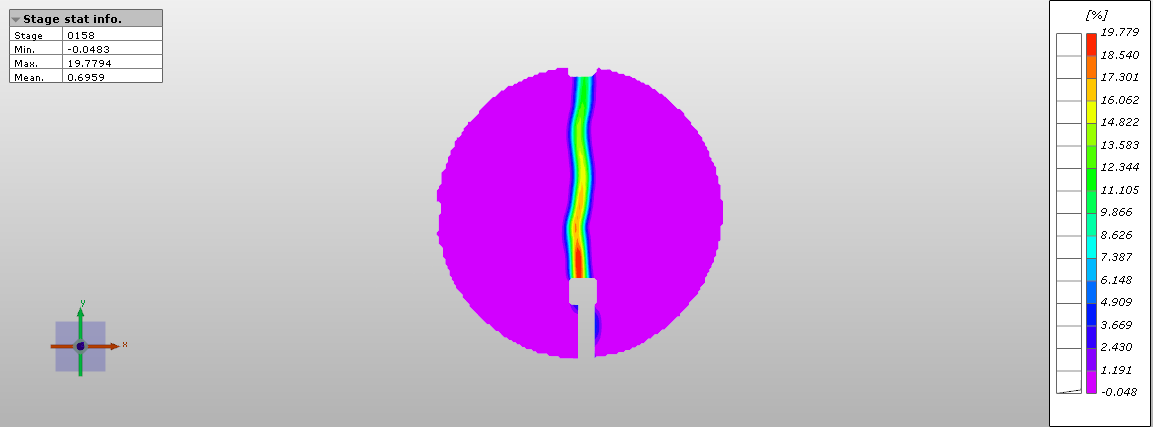

Supplement: S2 Data — (ZIP) [file pone.0294258.s002.zip › SNAPSERIES003/p0158.bmp]

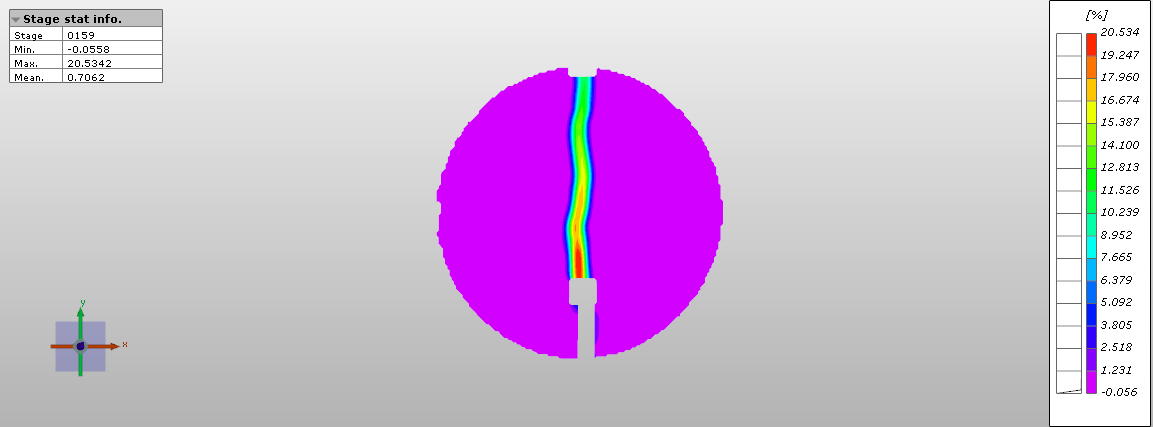

Supplement: S2 Data — (ZIP) [file pone.0294258.s002.zip › SNAPSERIES003/p0159.bmp]

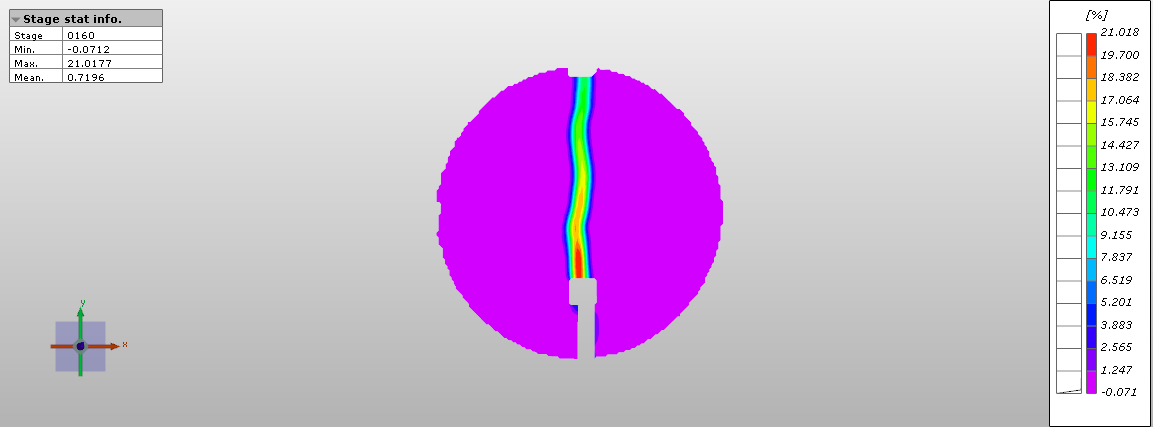

Supplement: S2 Data — (ZIP) [file pone.0294258.s002.zip › SNAPSERIES003/p0160.bmp]

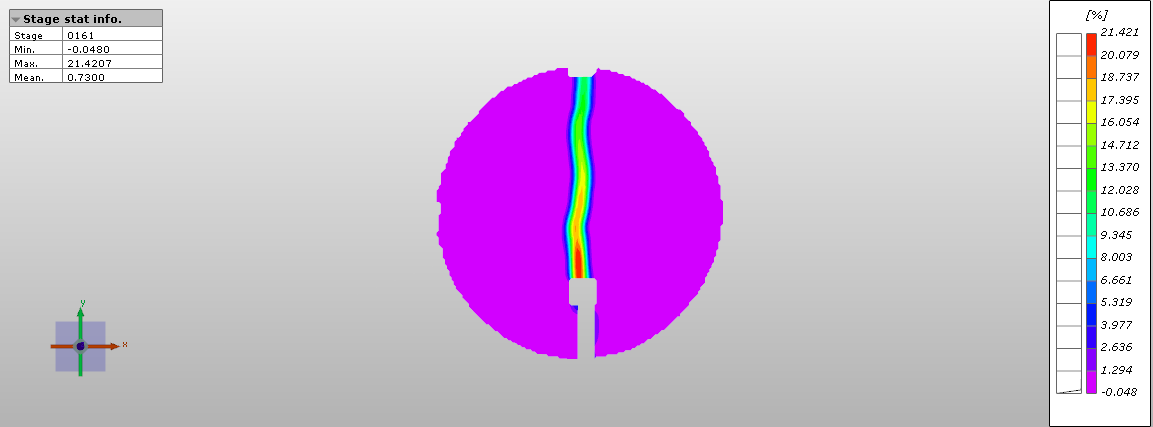

Supplement: S2 Data — (ZIP) [file pone.0294258.s002.zip › SNAPSERIES003/p0161.bmp]

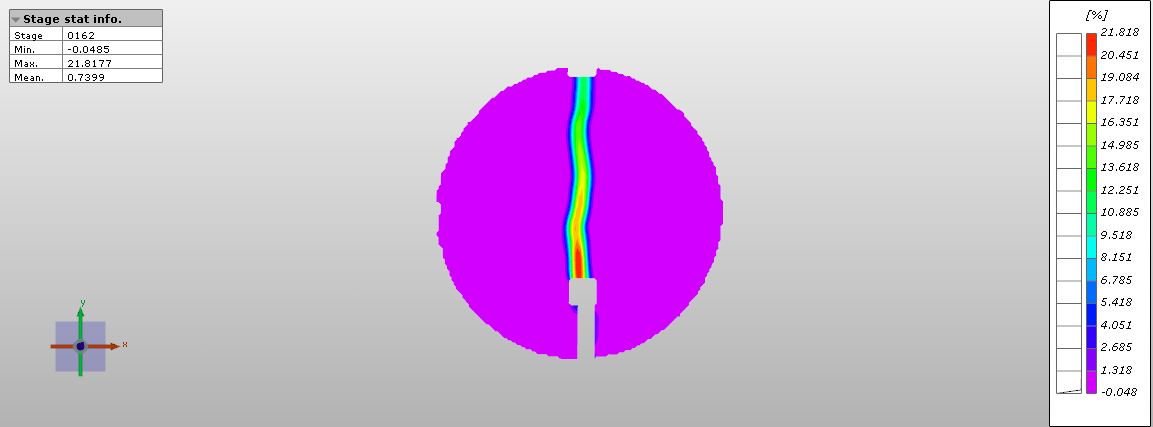

Supplement: S2 Data — (ZIP) [file pone.0294258.s002.zip › SNAPSERIES003/p0162.bmp]

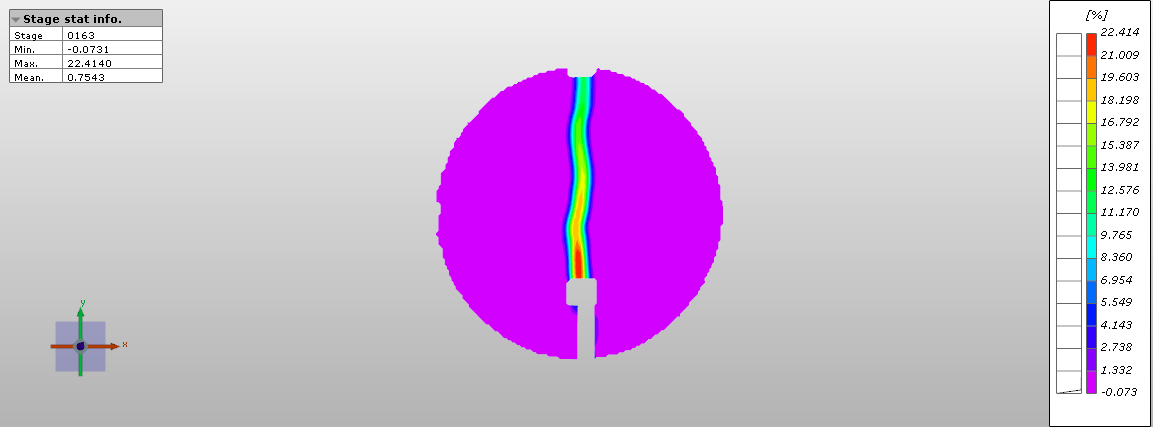

Supplement: S2 Data — (ZIP) [file pone.0294258.s002.zip › SNAPSERIES003/p0163.bmp]

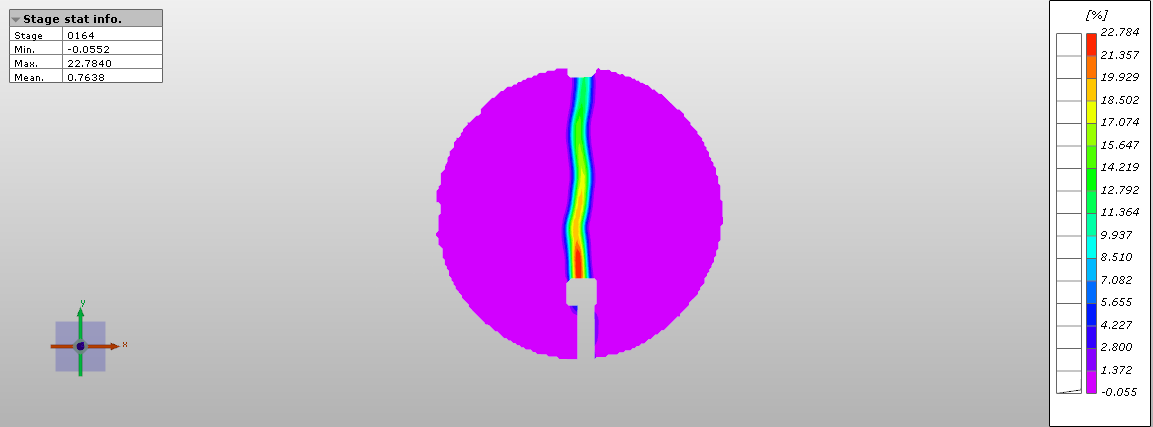

Supplement: S2 Data — (ZIP) [file pone.0294258.s002.zip › SNAPSERIES003/p0164.bmp]

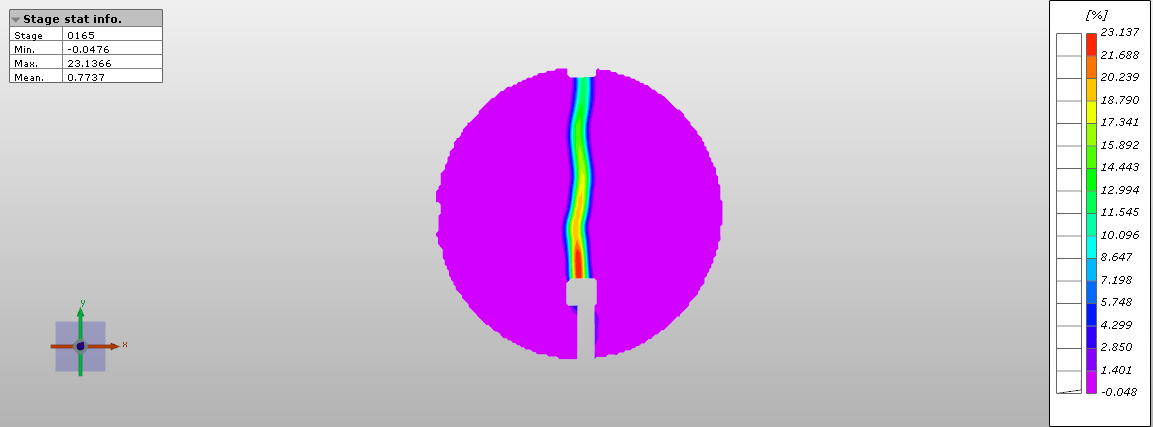

Supplement: S2 Data — (ZIP) [file pone.0294258.s002.zip › SNAPSERIES003/p0165.bmp]

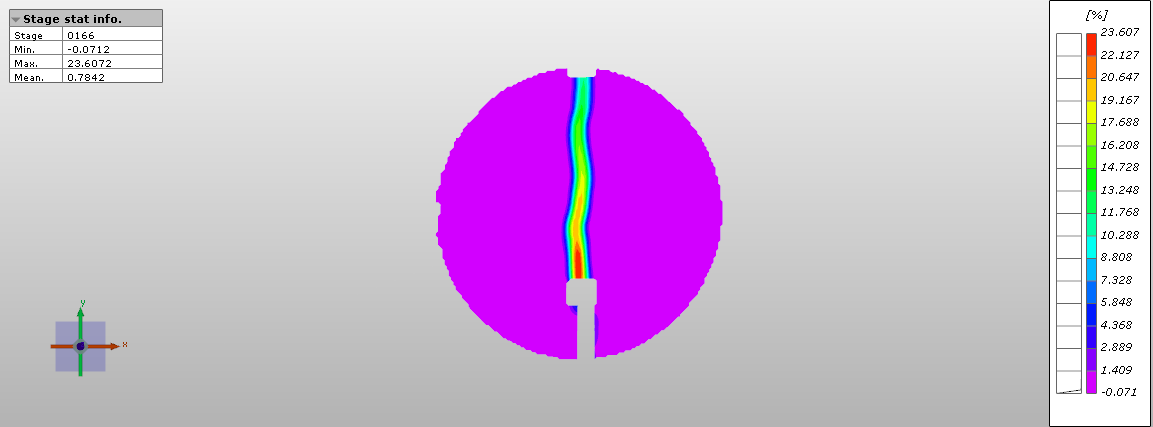

Supplement: S2 Data — (ZIP) [file pone.0294258.s002.zip › SNAPSERIES003/p0166.bmp]

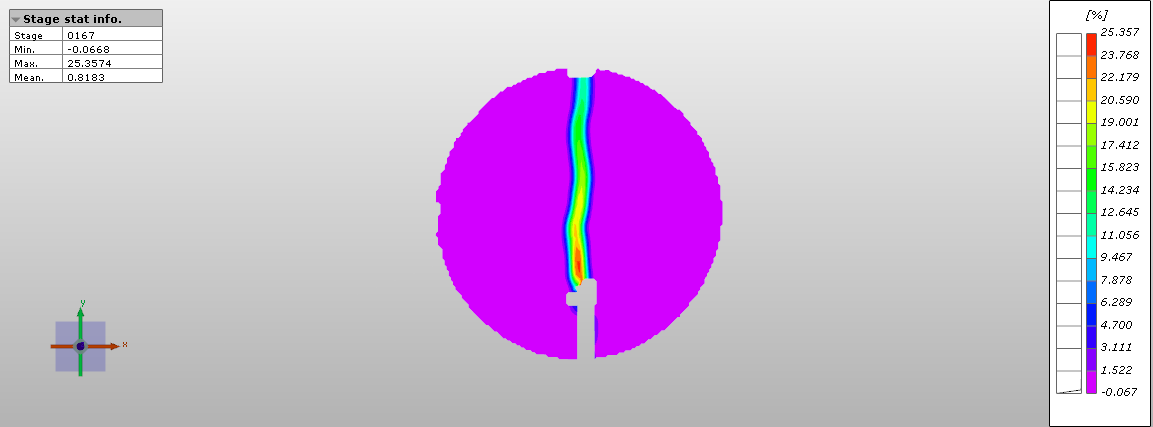

Supplement: S2 Data — (ZIP) [file pone.0294258.s002.zip › SNAPSERIES003/p0167.bmp]

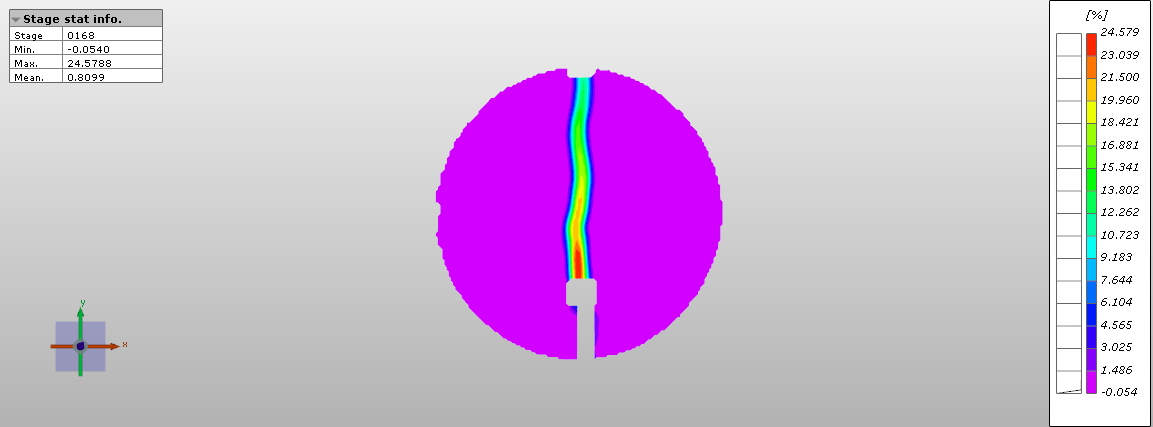

Supplement: S2 Data — (ZIP) [file pone.0294258.s002.zip › SNAPSERIES003/p0168.bmp]

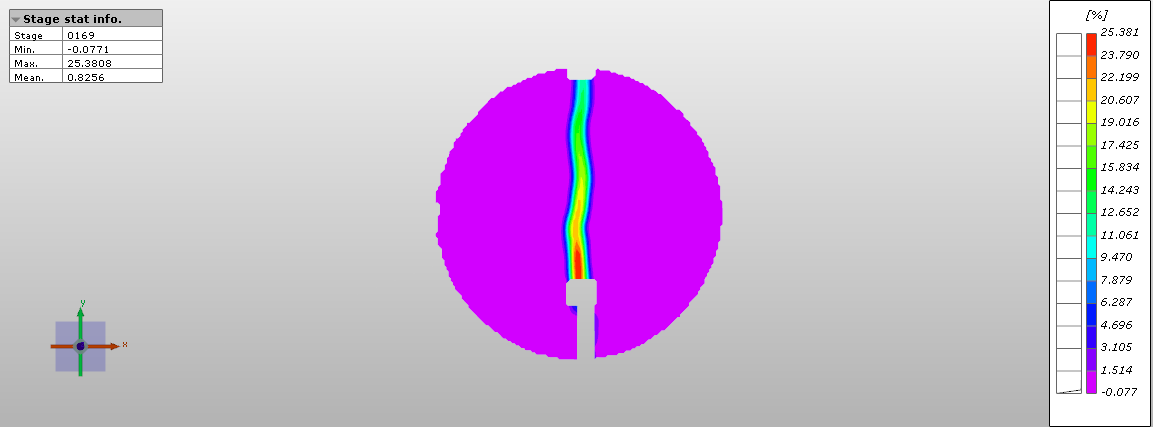

Supplement: S2 Data — (ZIP) [file pone.0294258.s002.zip › SNAPSERIES003/p0169.bmp]

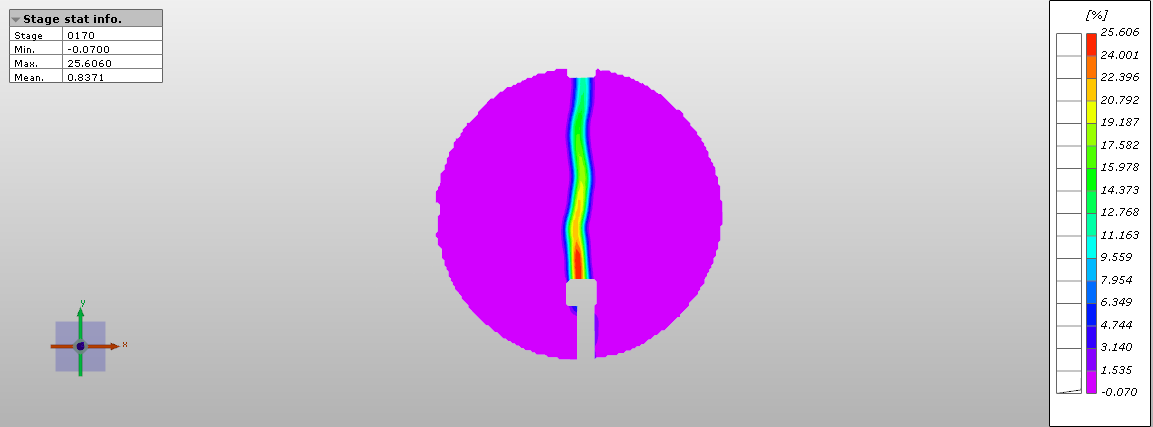

Supplement: S2 Data — (ZIP) [file pone.0294258.s002.zip › SNAPSERIES003/p0170.bmp]

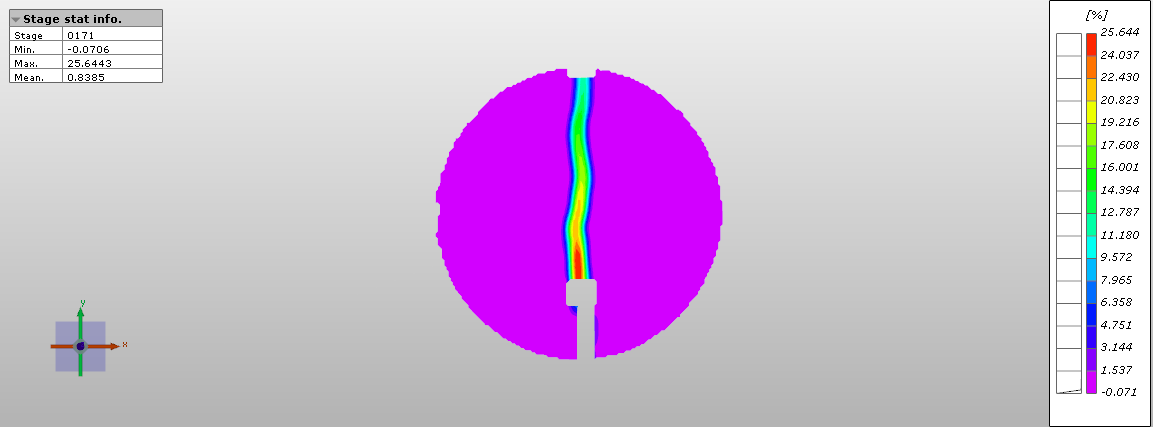

Supplement: S2 Data — (ZIP) [file pone.0294258.s002.zip › SNAPSERIES003/p0171.bmp]

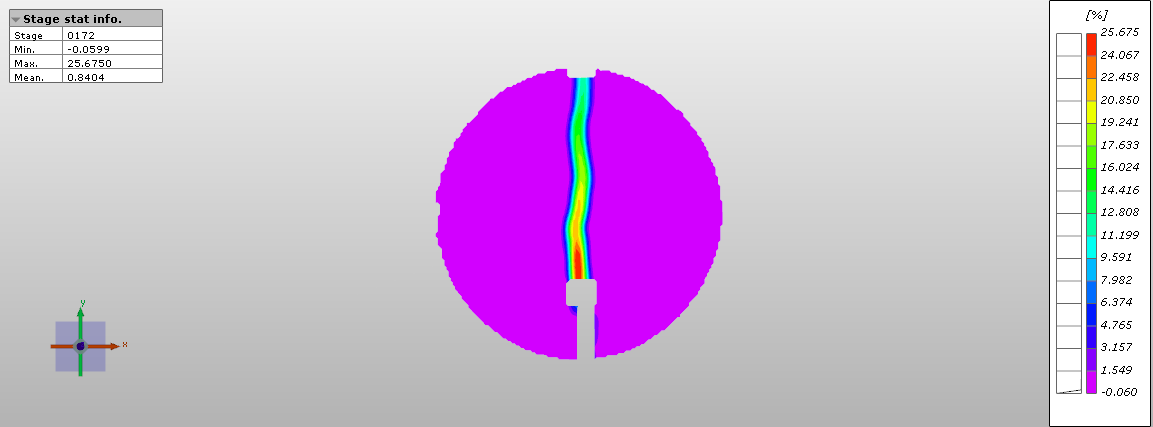

Supplement: S2 Data — (ZIP) [file pone.0294258.s002.zip › SNAPSERIES003/p0172.bmp]

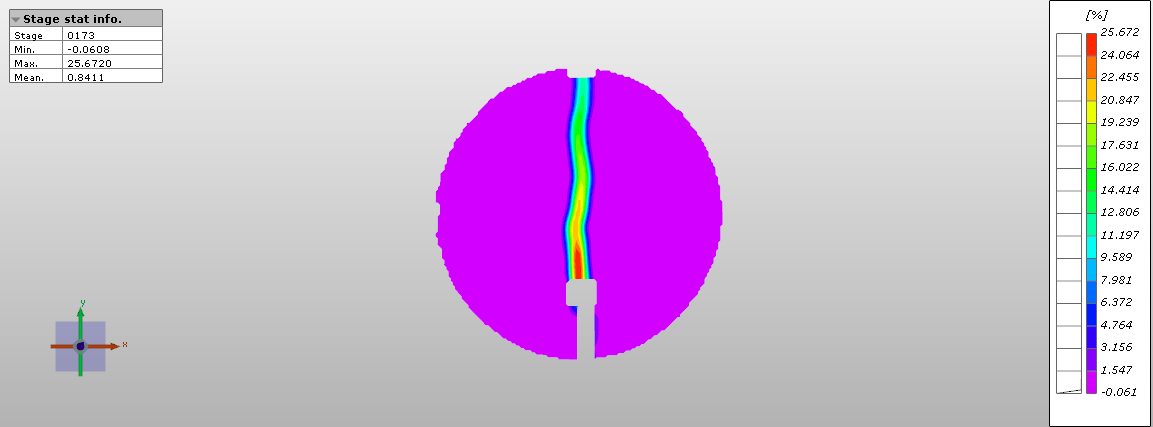

Supplement: S2 Data — (ZIP) [file pone.0294258.s002.zip › SNAPSERIES003/p0173.bmp]

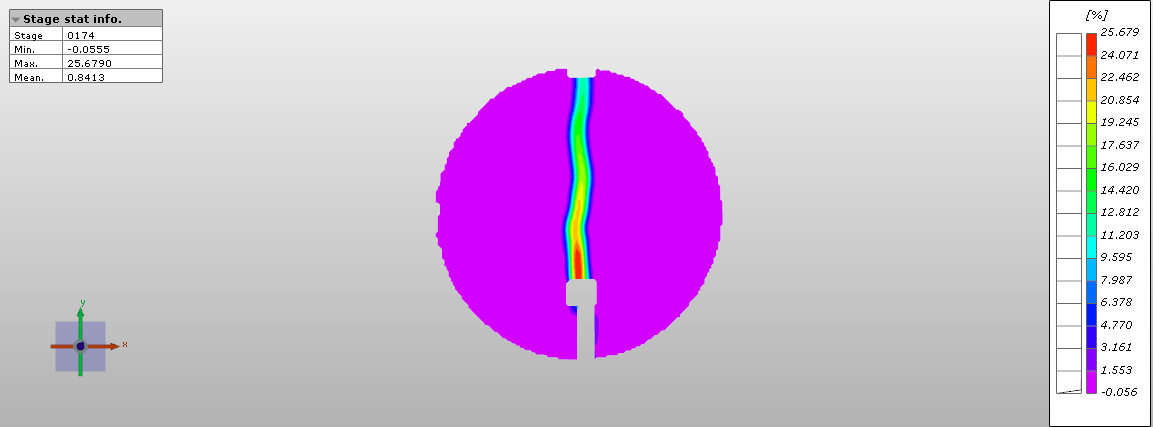

Supplement: S2 Data — (ZIP) [file pone.0294258.s002.zip › SNAPSERIES003/p0174.bmp]

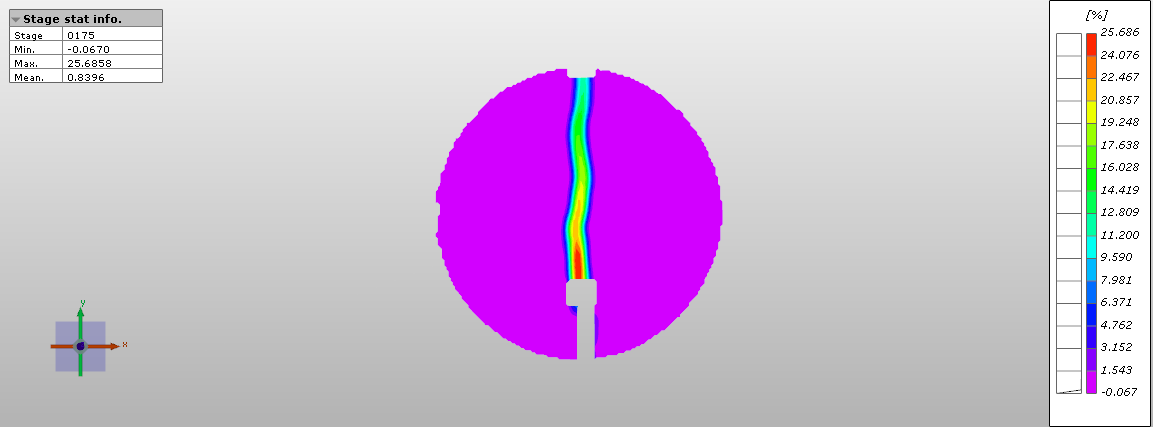

Supplement: S2 Data — (ZIP) [file pone.0294258.s002.zip › SNAPSERIES003/p0175.bmp]

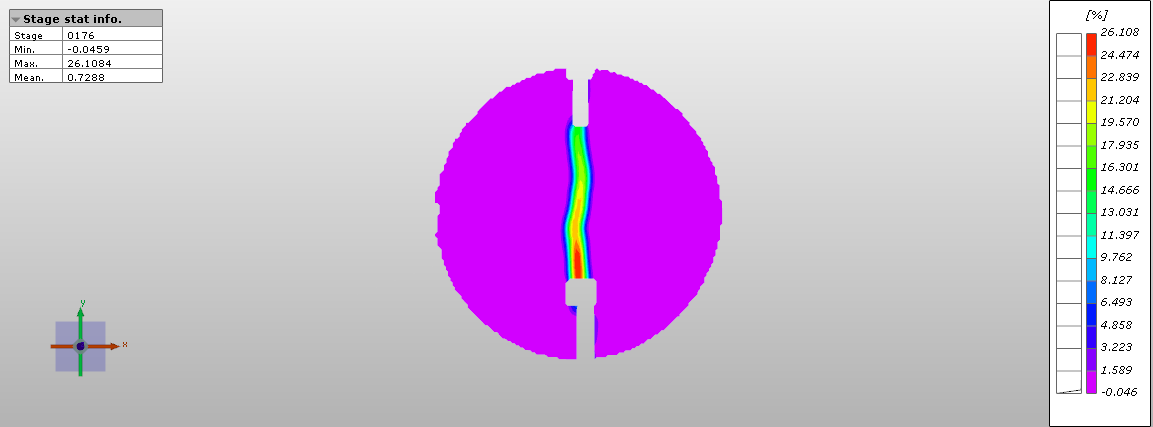

Supplement: S2 Data — (ZIP) [file pone.0294258.s002.zip › SNAPSERIES003/p0176.bmp]

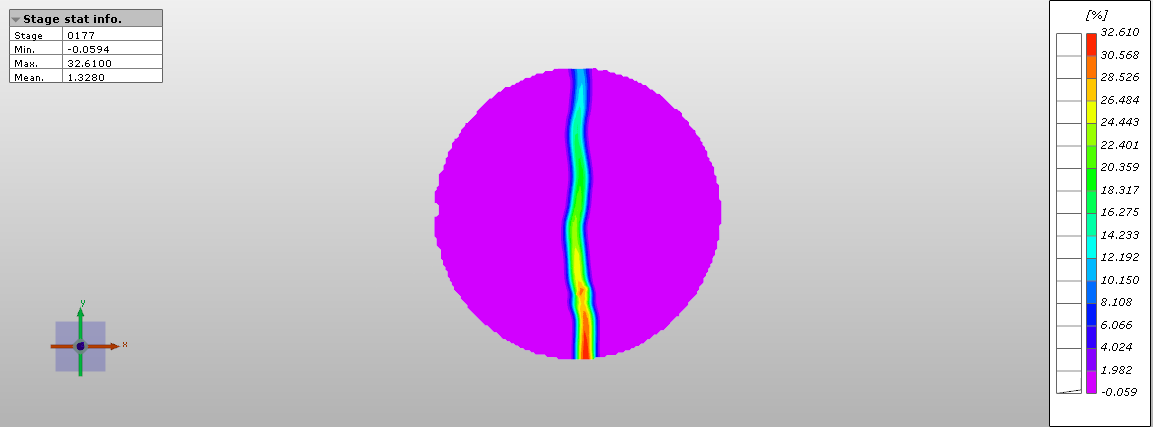

Supplement: S2 Data — (ZIP) [file pone.0294258.s002.zip › SNAPSERIES003/p0177.bmp]
