# Supplementary figures and images for: Macro/Microfracture evolution and instability behaviors of high-temperature granite under water-cooling subjected to Brazilian splitting test using the DIC technique (part 1 of 2)
Source: PLoS One. 2023 Nov 29;18(11):e0294258. doi: 10.1371/journal.pone.0294258 (PMC10686506; doi:10.1371/journal.pone.0294258)

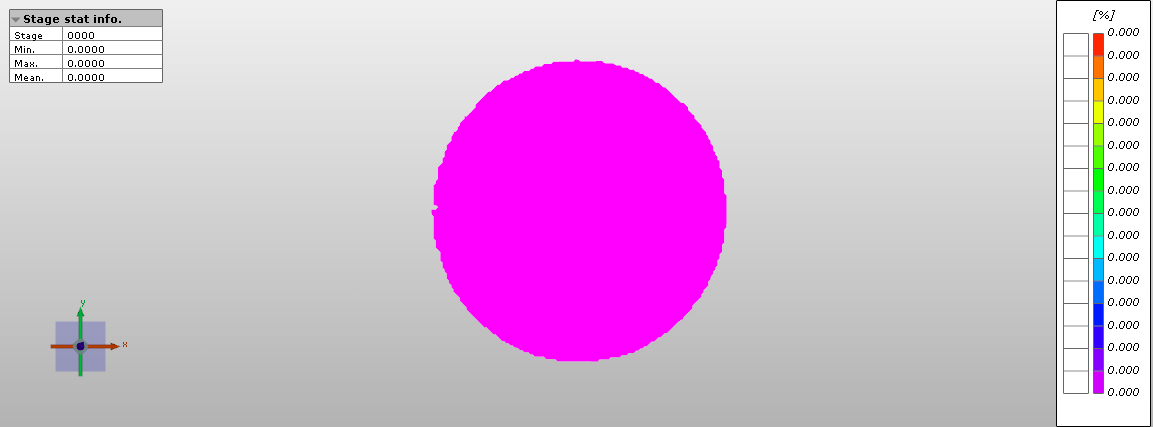

Supplement: S2 Data — (ZIP) [file pone.0294258.s002.zip › SNAPSERIES003/p0000.bmp]

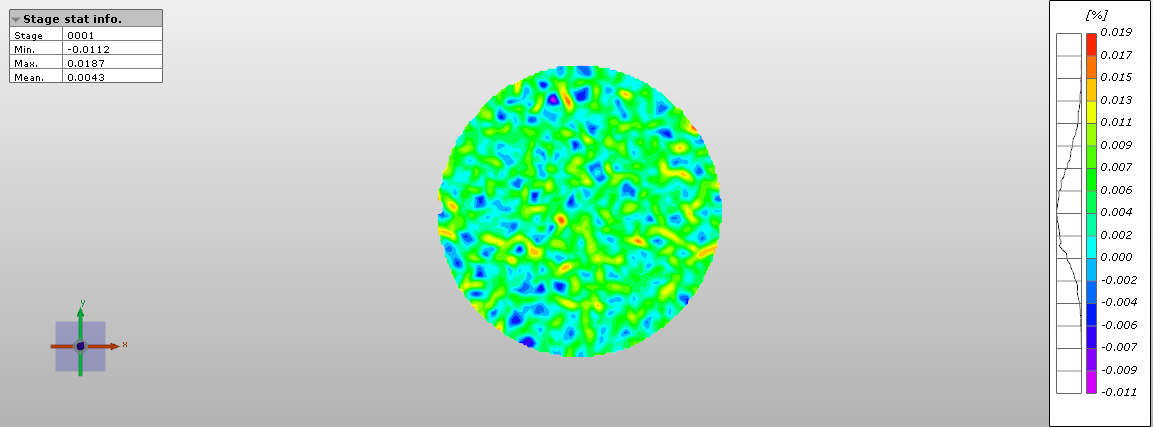

Supplement: S2 Data — (ZIP) [file pone.0294258.s002.zip › SNAPSERIES003/p0001.bmp]

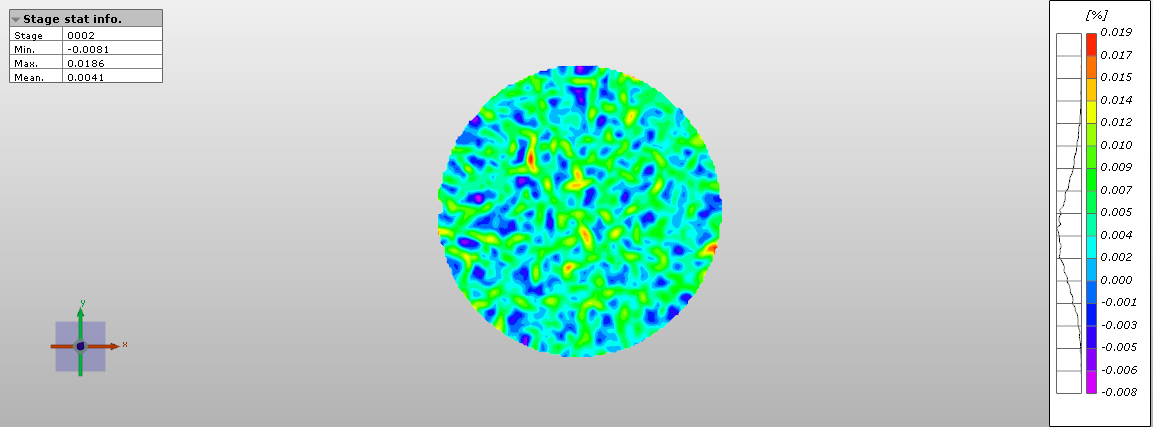

Supplement: S2 Data — (ZIP) [file pone.0294258.s002.zip › SNAPSERIES003/p0002.bmp]

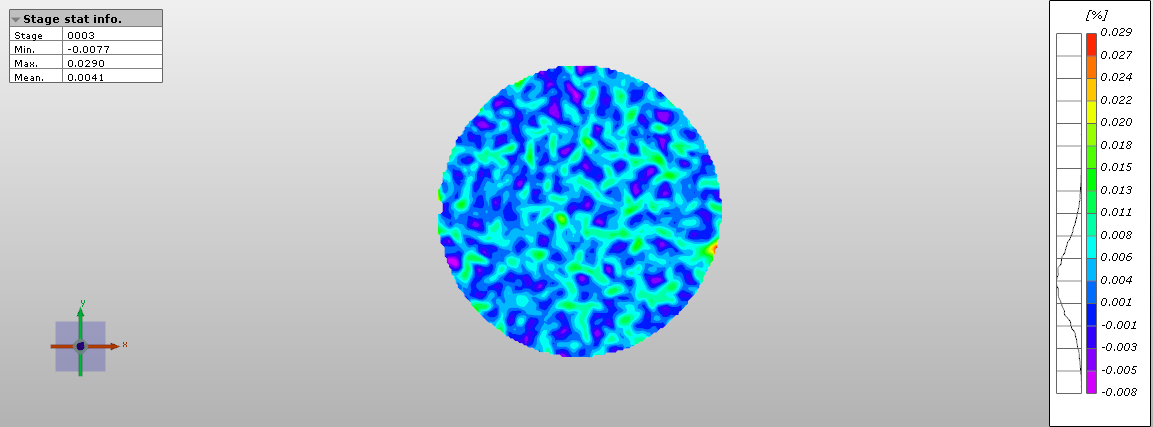

Supplement: S2 Data — (ZIP) [file pone.0294258.s002.zip › SNAPSERIES003/p0003.bmp]

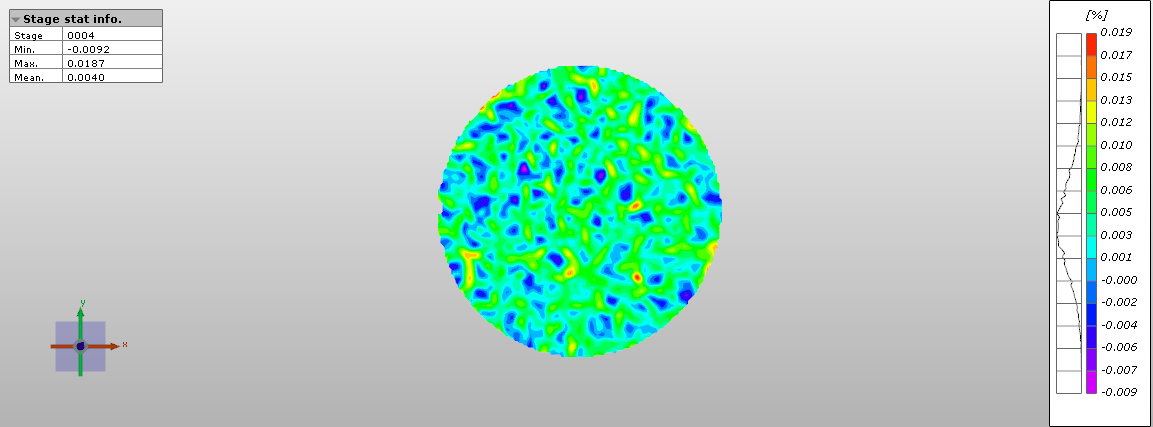

Supplement: S2 Data — (ZIP) [file pone.0294258.s002.zip › SNAPSERIES003/p0004.bmp]

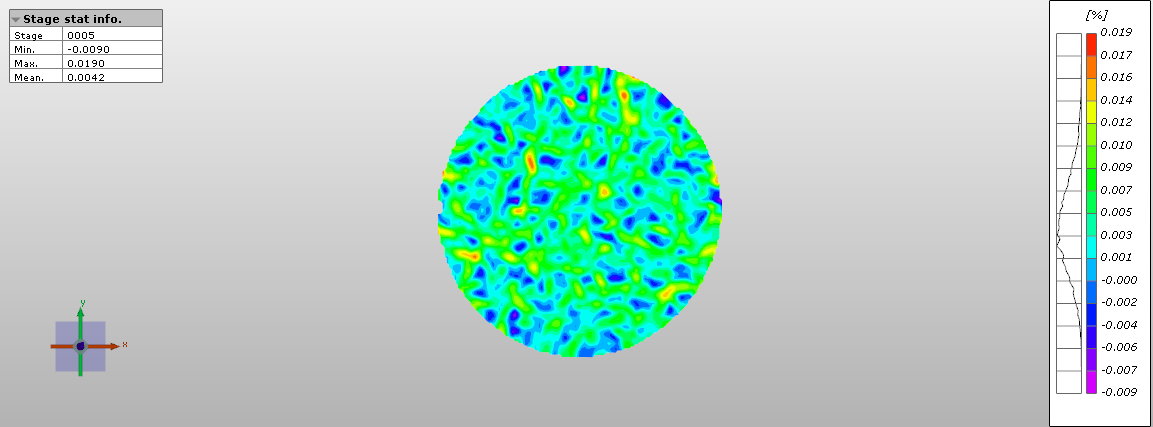

Supplement: S2 Data — (ZIP) [file pone.0294258.s002.zip › SNAPSERIES003/p0005.bmp]

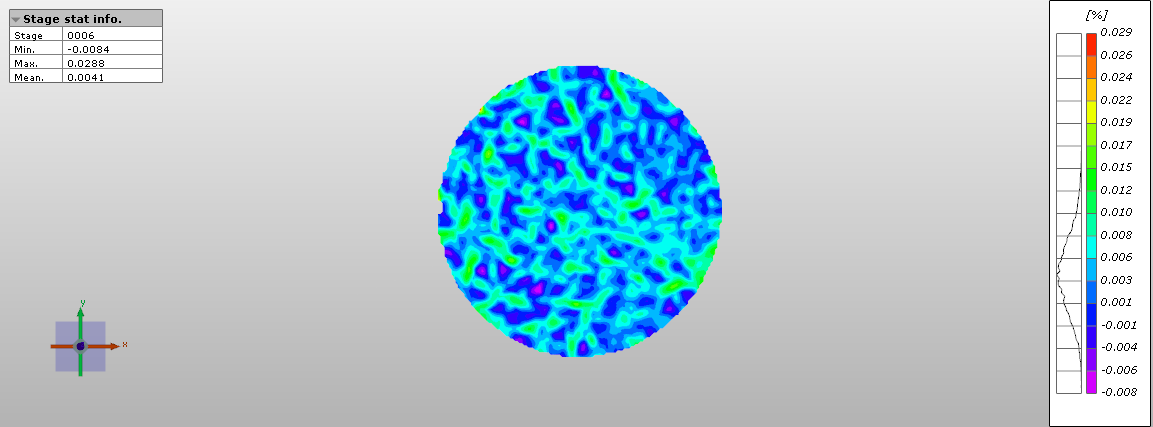

Supplement: S2 Data — (ZIP) [file pone.0294258.s002.zip › SNAPSERIES003/p0006.bmp]

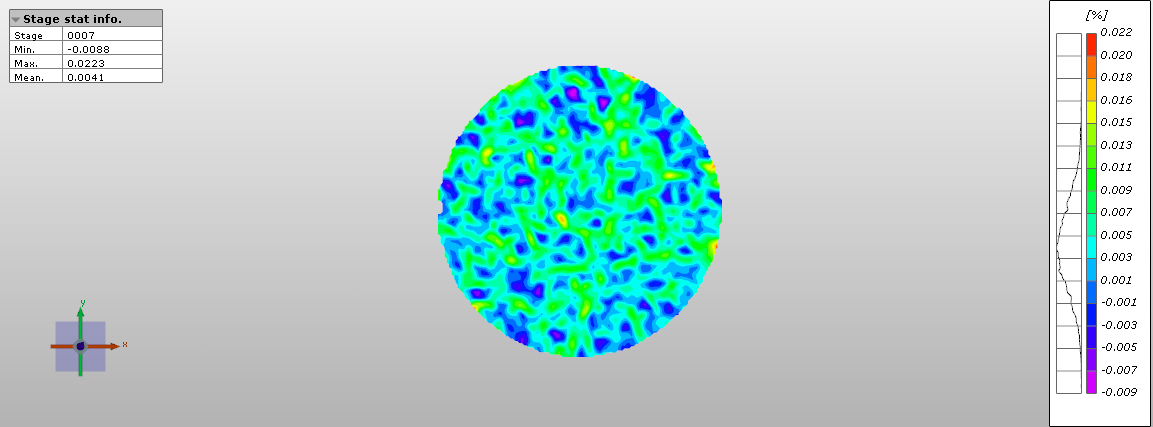

Supplement: S2 Data — (ZIP) [file pone.0294258.s002.zip › SNAPSERIES003/p0007.bmp]

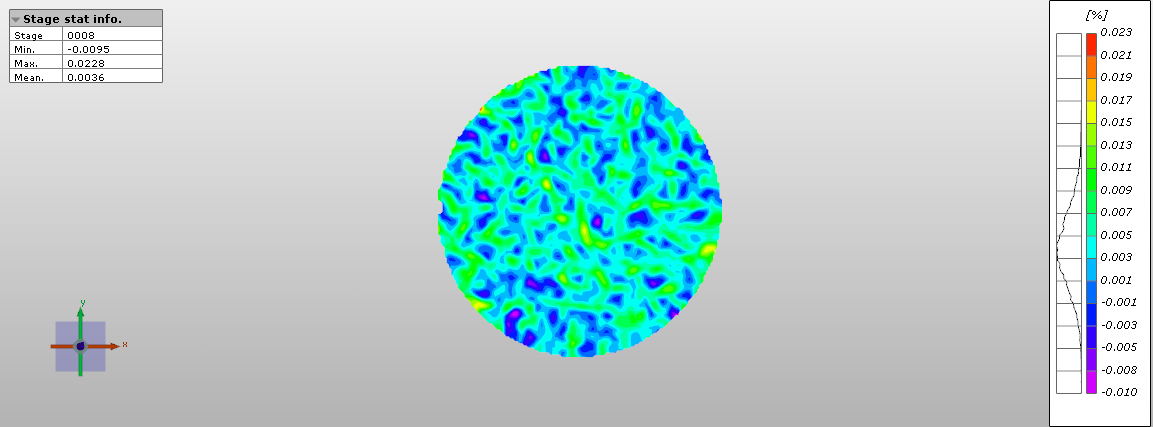

Supplement: S2 Data — (ZIP) [file pone.0294258.s002.zip › SNAPSERIES003/p0008.bmp]

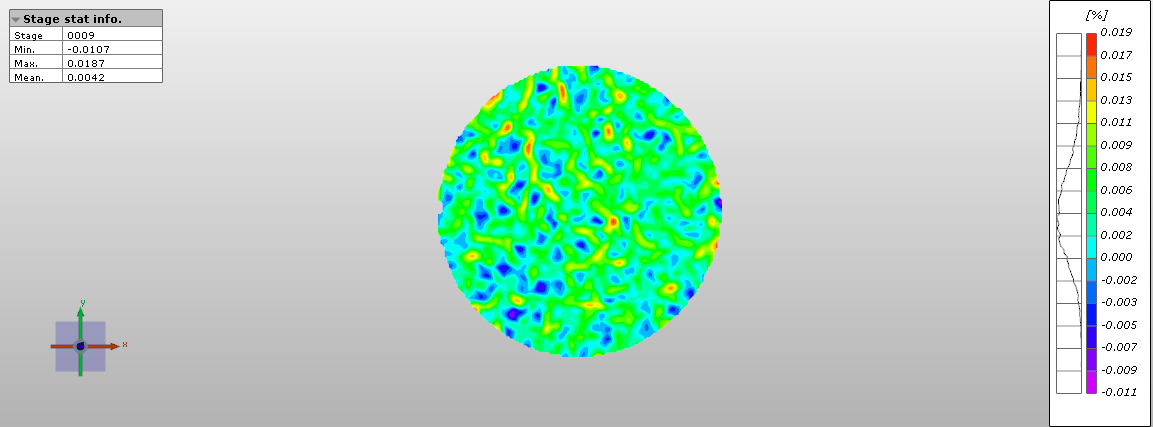

Supplement: S2 Data — (ZIP) [file pone.0294258.s002.zip › SNAPSERIES003/p0009.bmp]

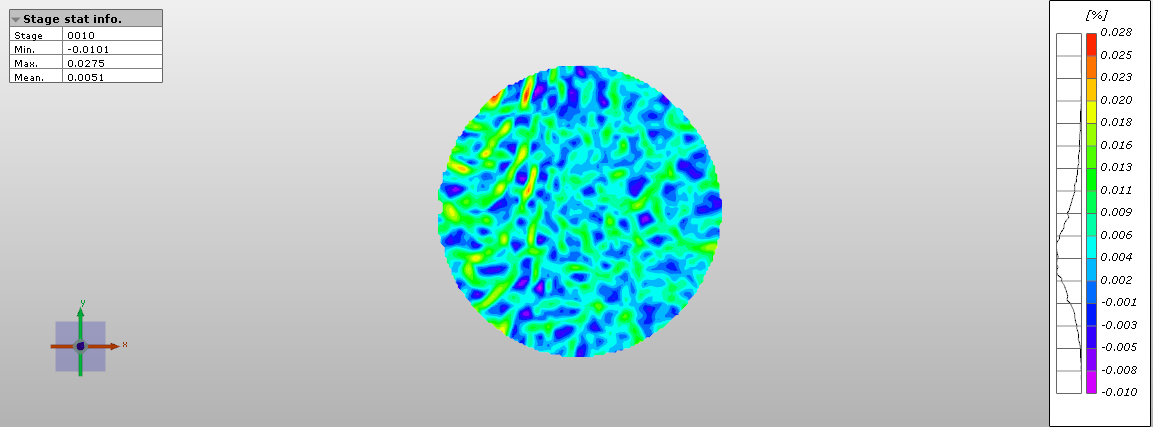

Supplement: S2 Data — (ZIP) [file pone.0294258.s002.zip › SNAPSERIES003/p0010.bmp]

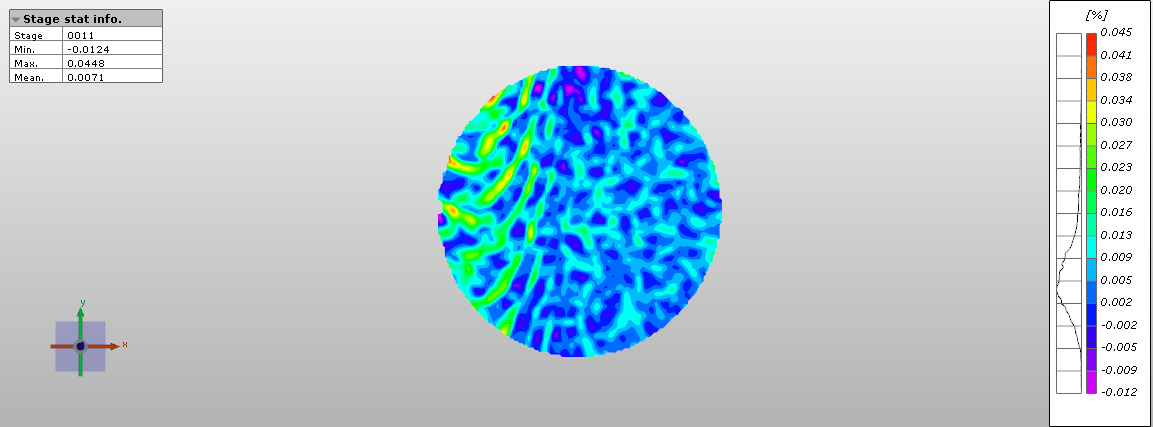

Supplement: S2 Data — (ZIP) [file pone.0294258.s002.zip › SNAPSERIES003/p0011.bmp]

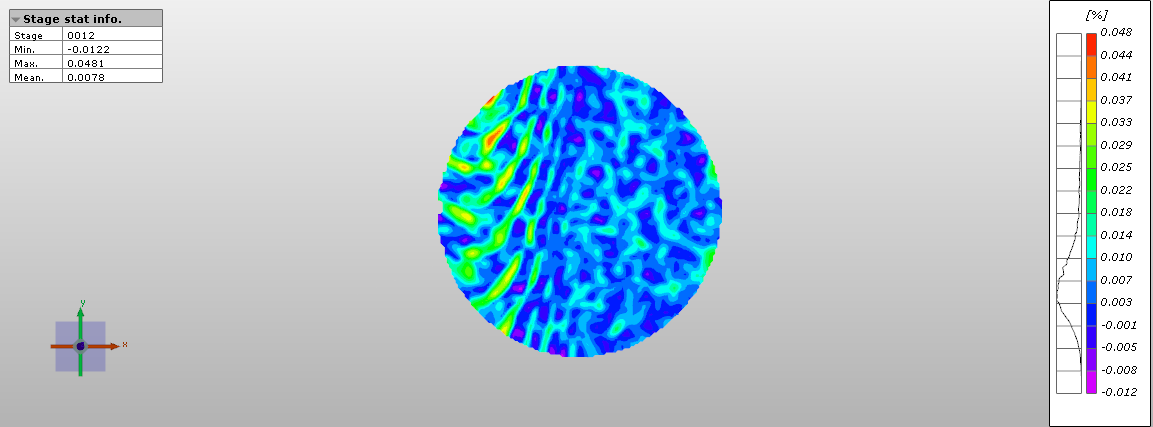

Supplement: S2 Data — (ZIP) [file pone.0294258.s002.zip › SNAPSERIES003/p0012.bmp]

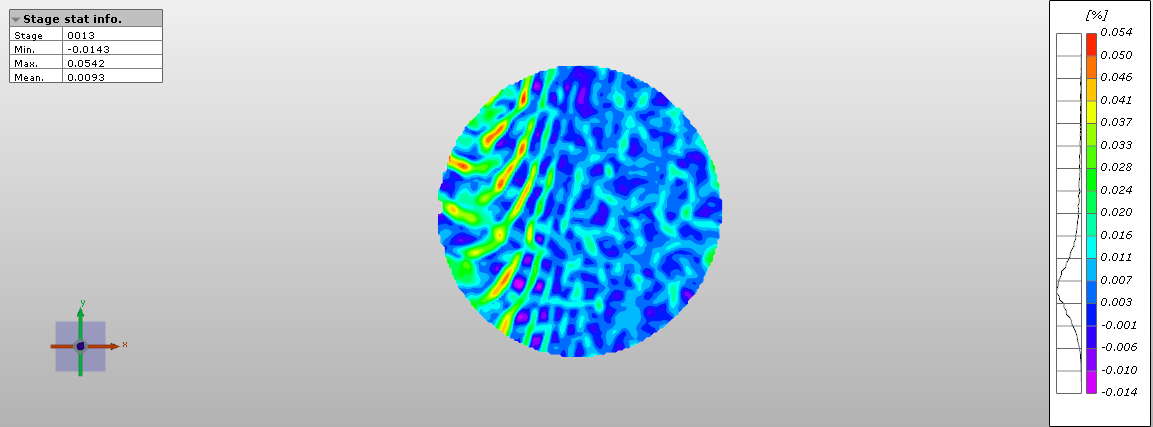

Supplement: S2 Data — (ZIP) [file pone.0294258.s002.zip › SNAPSERIES003/p0013.bmp]

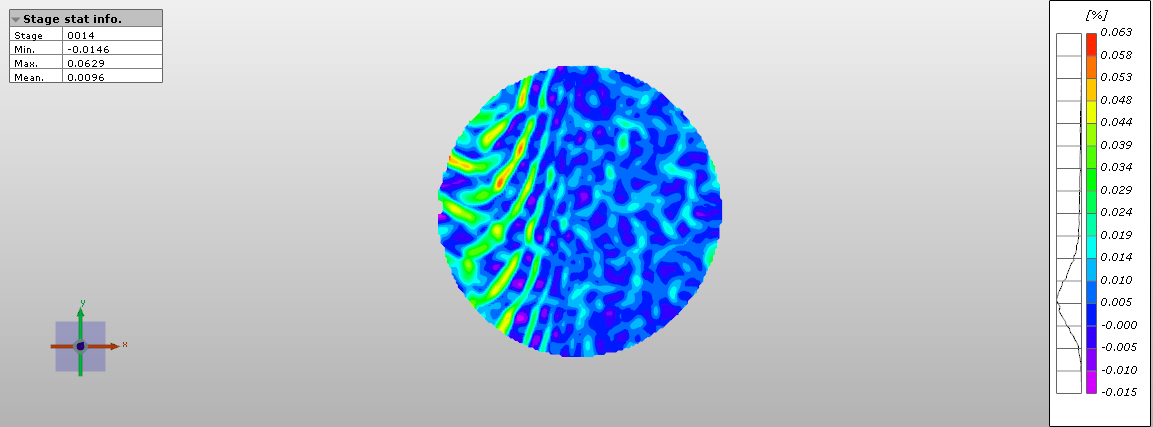

Supplement: S2 Data — (ZIP) [file pone.0294258.s002.zip › SNAPSERIES003/p0014.bmp]

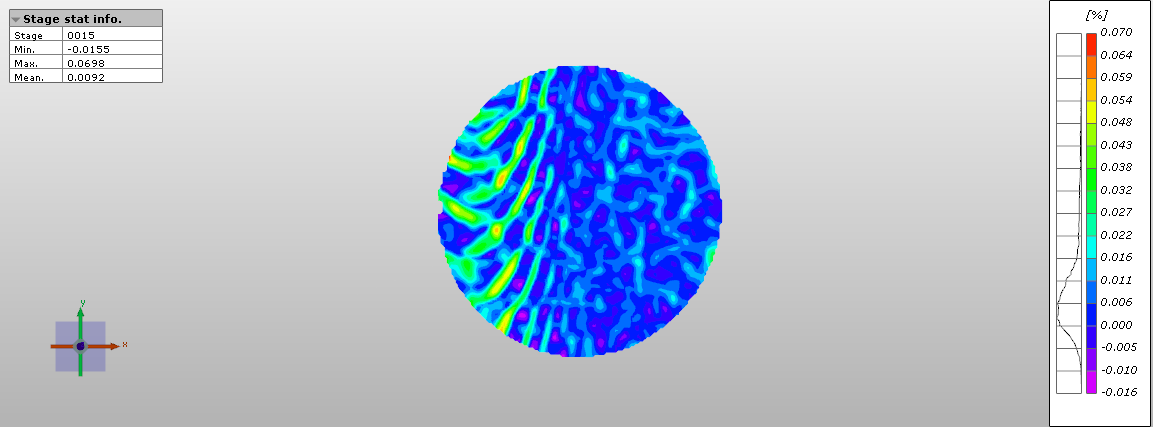

Supplement: S2 Data — (ZIP) [file pone.0294258.s002.zip › SNAPSERIES003/p0015.bmp]

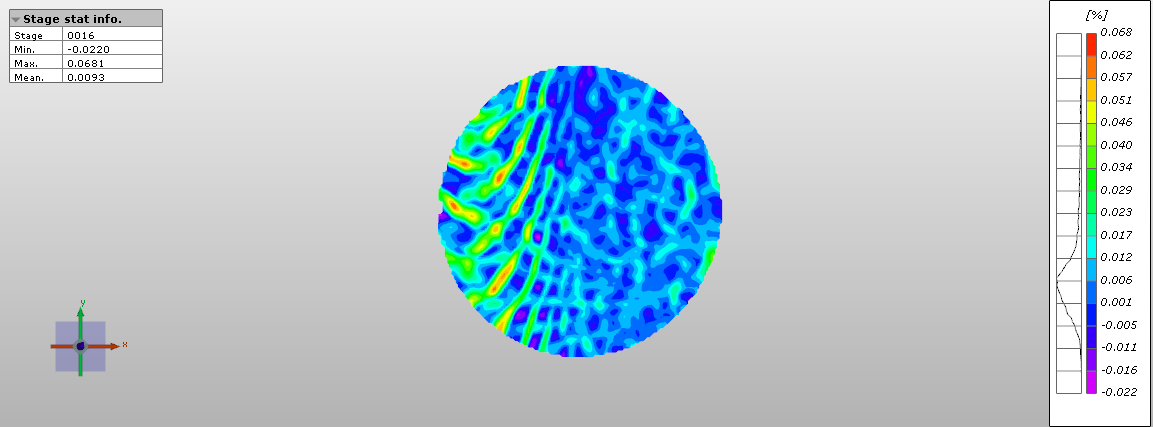

Supplement: S2 Data — (ZIP) [file pone.0294258.s002.zip › SNAPSERIES003/p0016.bmp]

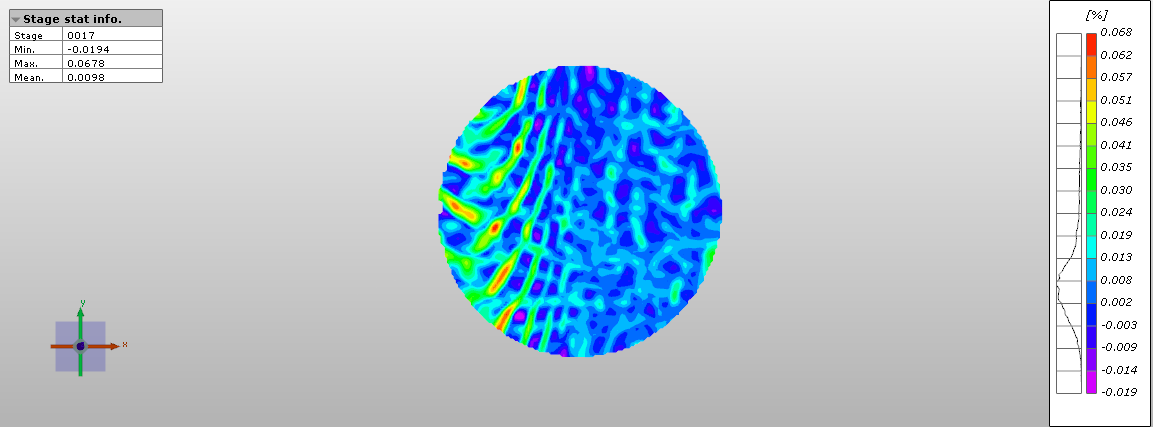

Supplement: S2 Data — (ZIP) [file pone.0294258.s002.zip › SNAPSERIES003/p0017.bmp]

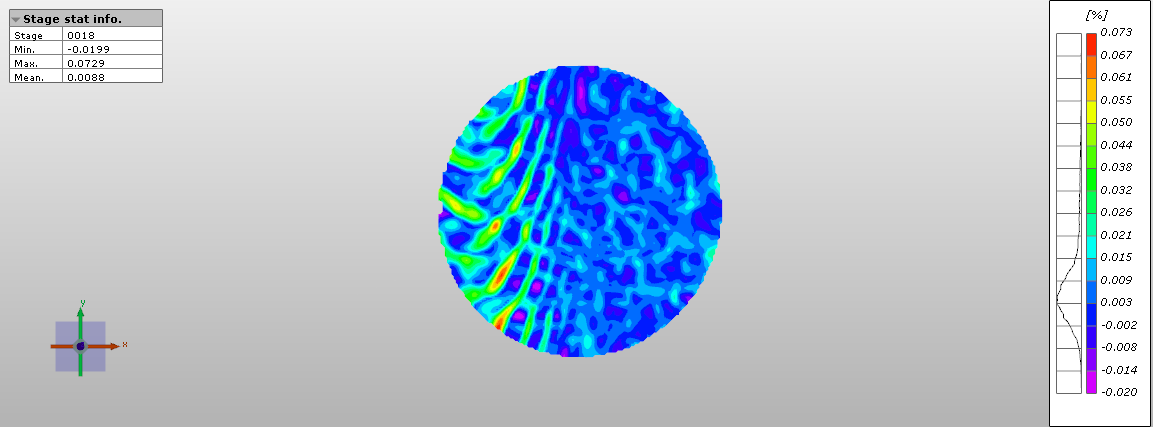

Supplement: S2 Data — (ZIP) [file pone.0294258.s002.zip › SNAPSERIES003/p0018.bmp]

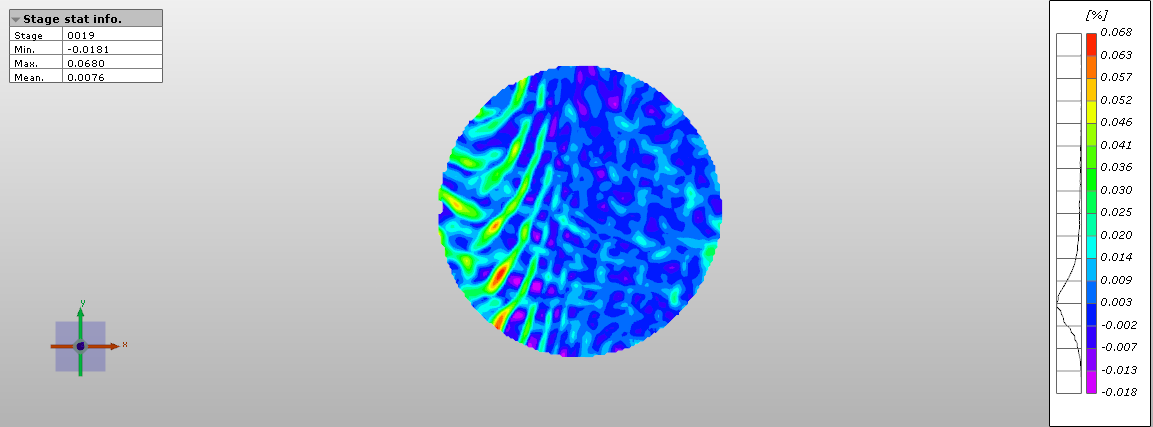

Supplement: S2 Data — (ZIP) [file pone.0294258.s002.zip › SNAPSERIES003/p0019.bmp]

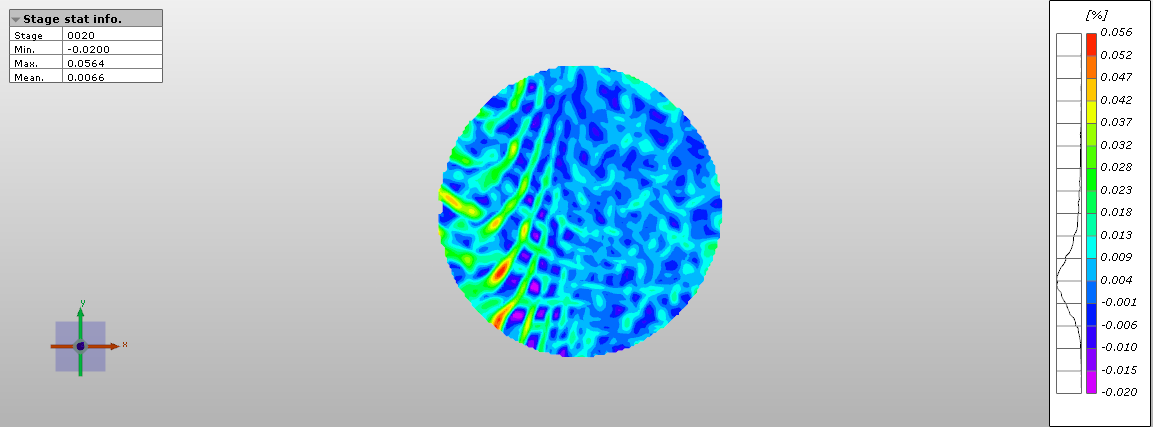

Supplement: S2 Data — (ZIP) [file pone.0294258.s002.zip › SNAPSERIES003/p0020.bmp]

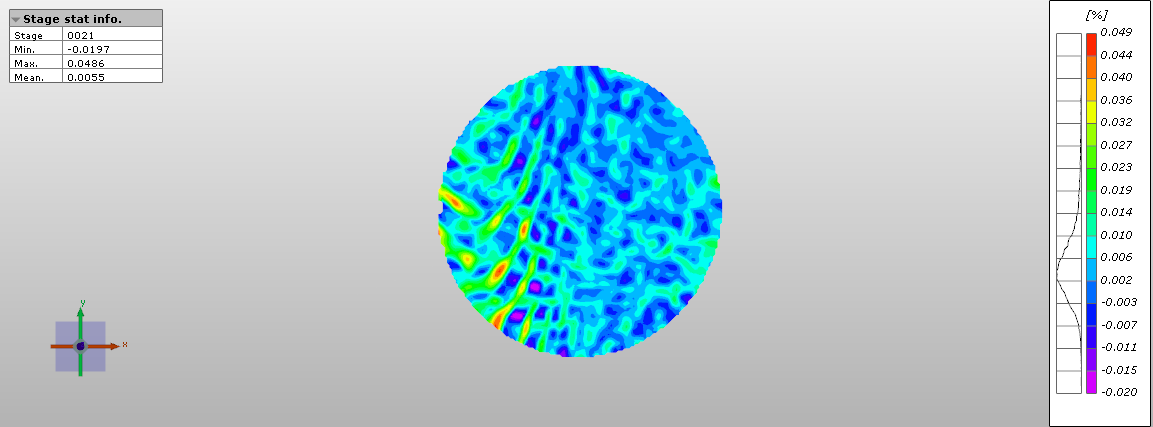

Supplement: S2 Data — (ZIP) [file pone.0294258.s002.zip › SNAPSERIES003/p0021.bmp]

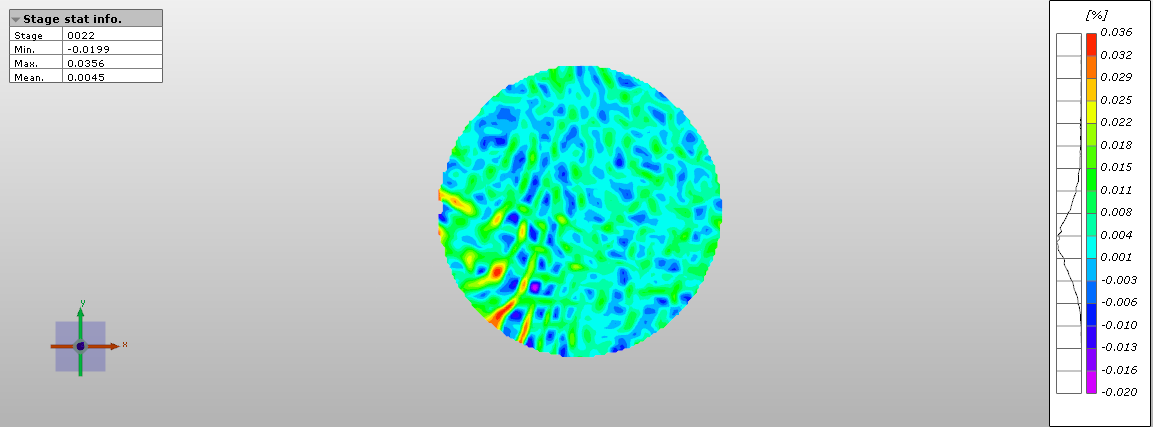

Supplement: S2 Data — (ZIP) [file pone.0294258.s002.zip › SNAPSERIES003/p0022.bmp]

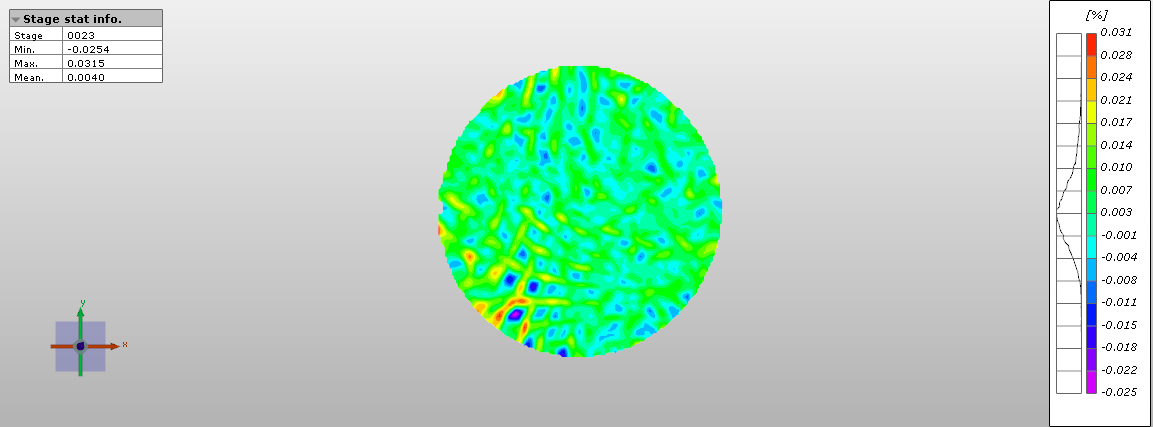

Supplement: S2 Data — (ZIP) [file pone.0294258.s002.zip › SNAPSERIES003/p0023.bmp]

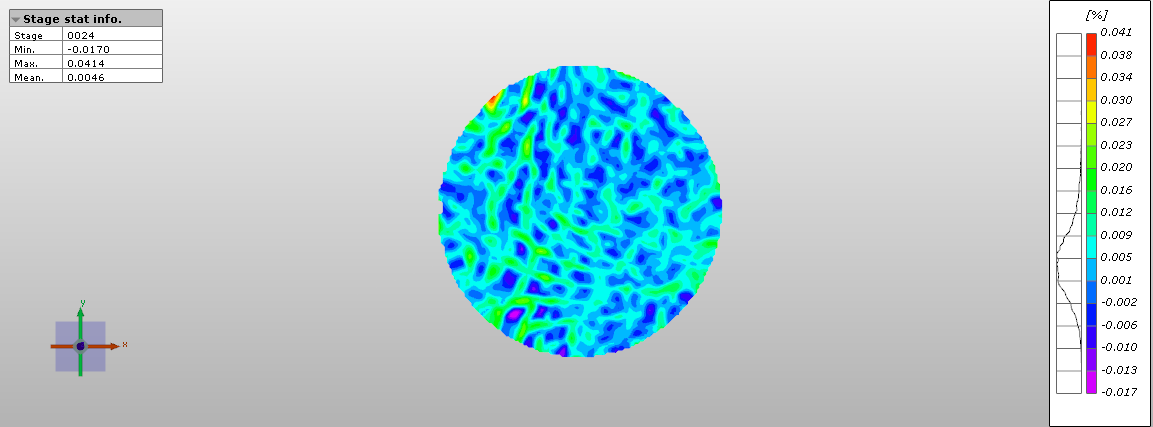

Supplement: S2 Data — (ZIP) [file pone.0294258.s002.zip › SNAPSERIES003/p0024.bmp]

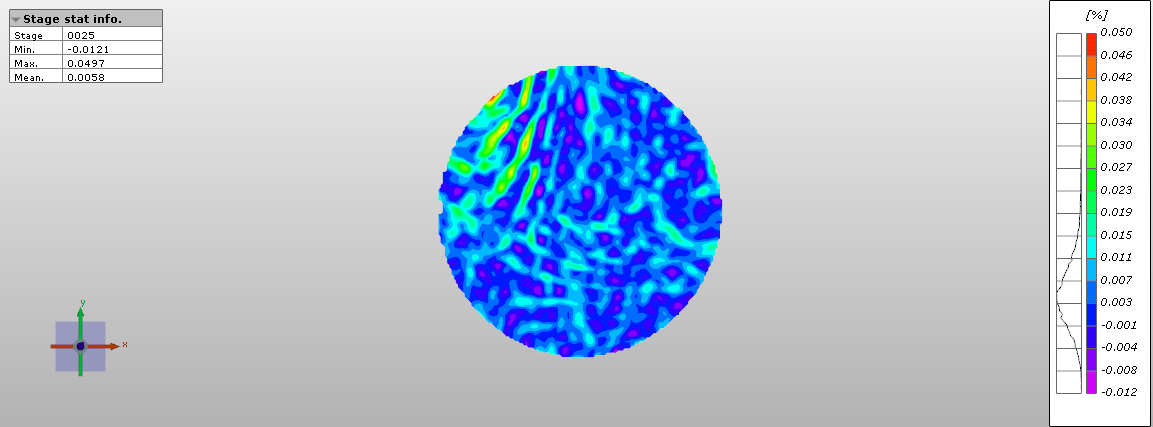

Supplement: S2 Data — (ZIP) [file pone.0294258.s002.zip › SNAPSERIES003/p0025.bmp]

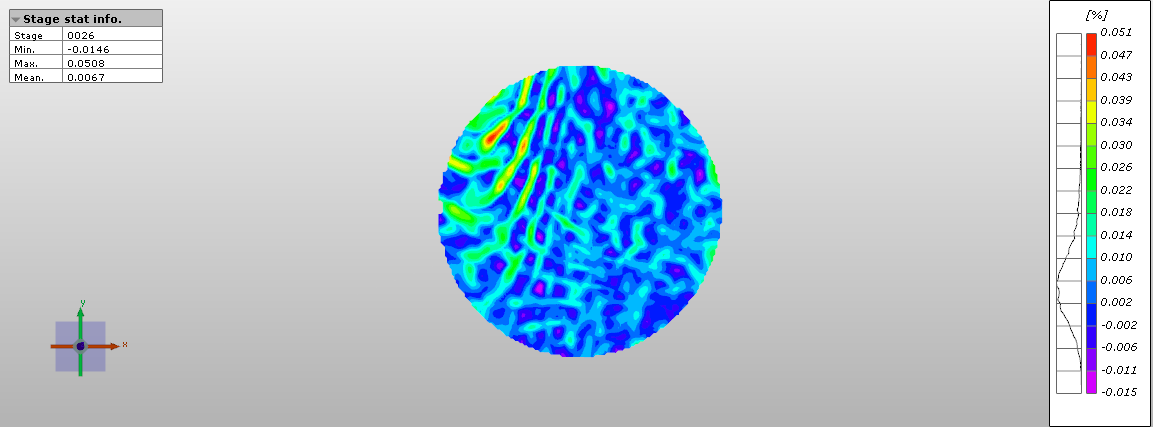

Supplement: S2 Data — (ZIP) [file pone.0294258.s002.zip › SNAPSERIES003/p0026.bmp]

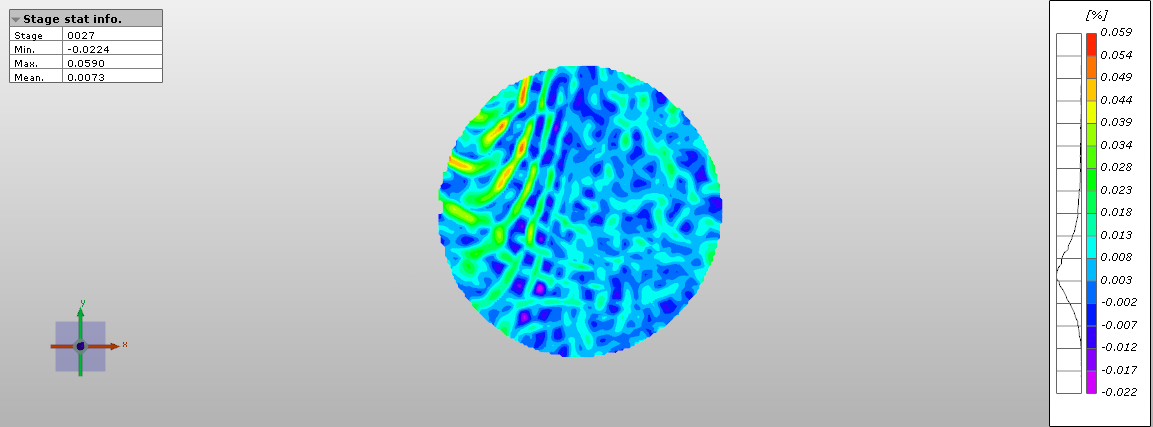

Supplement: S2 Data — (ZIP) [file pone.0294258.s002.zip › SNAPSERIES003/p0027.bmp]

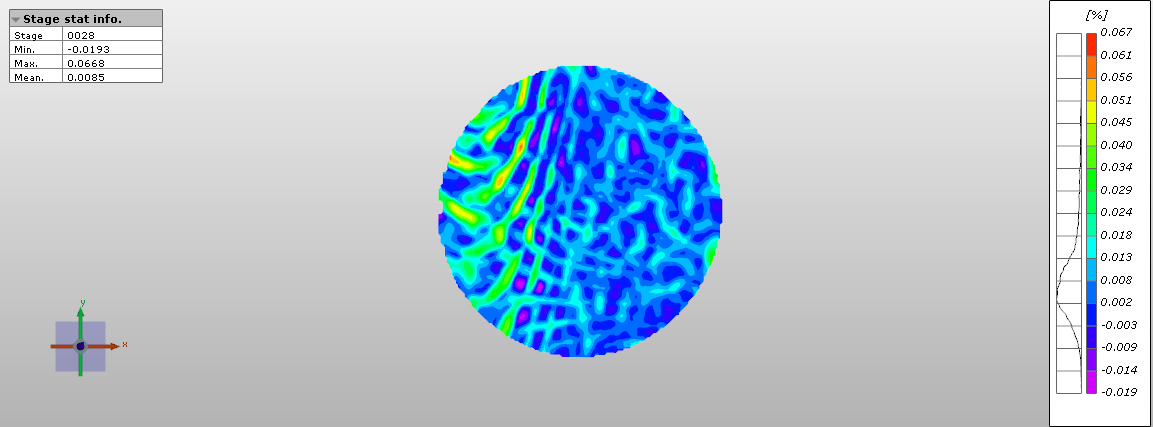

Supplement: S2 Data — (ZIP) [file pone.0294258.s002.zip › SNAPSERIES003/p0028.bmp]

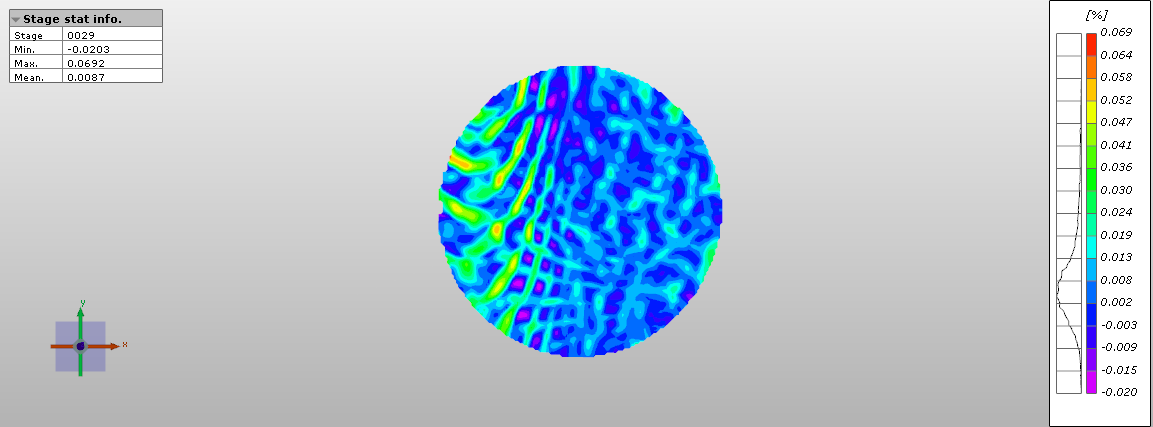

Supplement: S2 Data — (ZIP) [file pone.0294258.s002.zip › SNAPSERIES003/p0029.bmp]

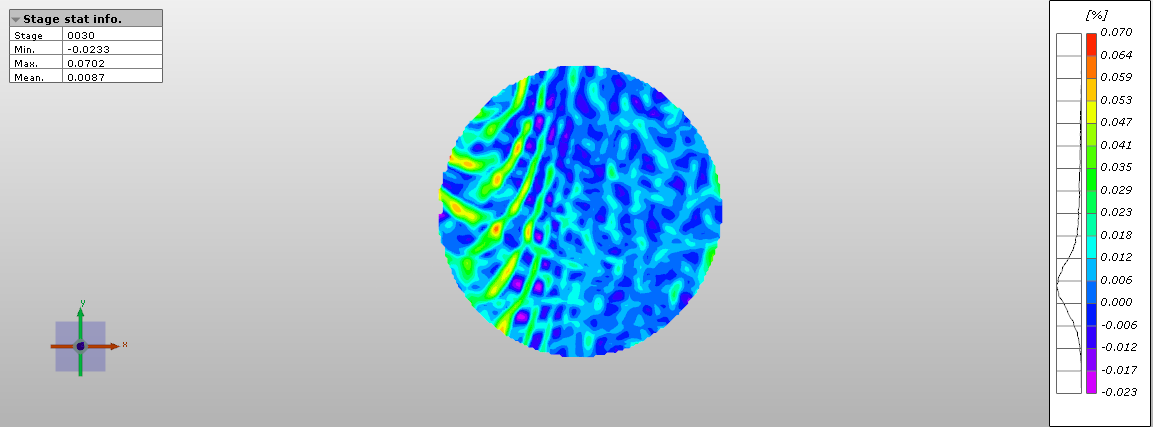

Supplement: S2 Data — (ZIP) [file pone.0294258.s002.zip › SNAPSERIES003/p0030.bmp]

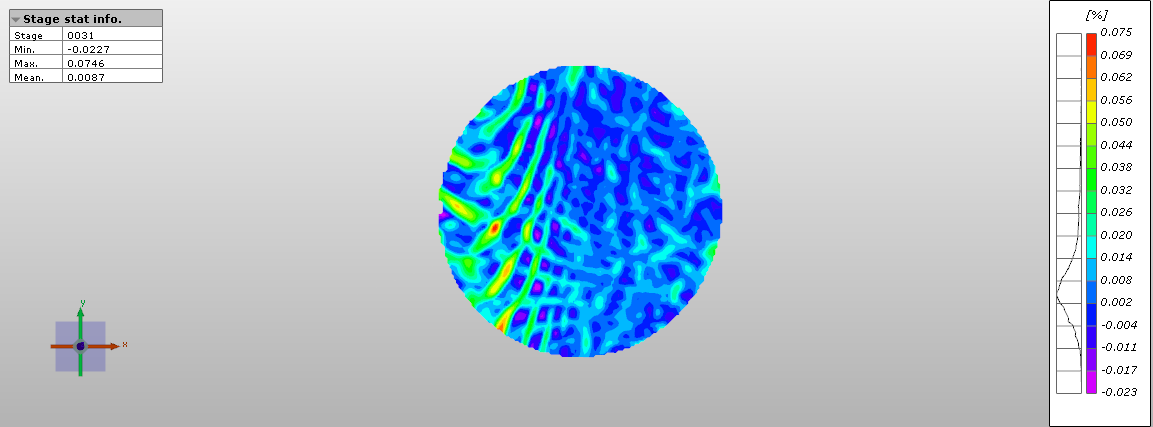

Supplement: S2 Data — (ZIP) [file pone.0294258.s002.zip › SNAPSERIES003/p0031.bmp]

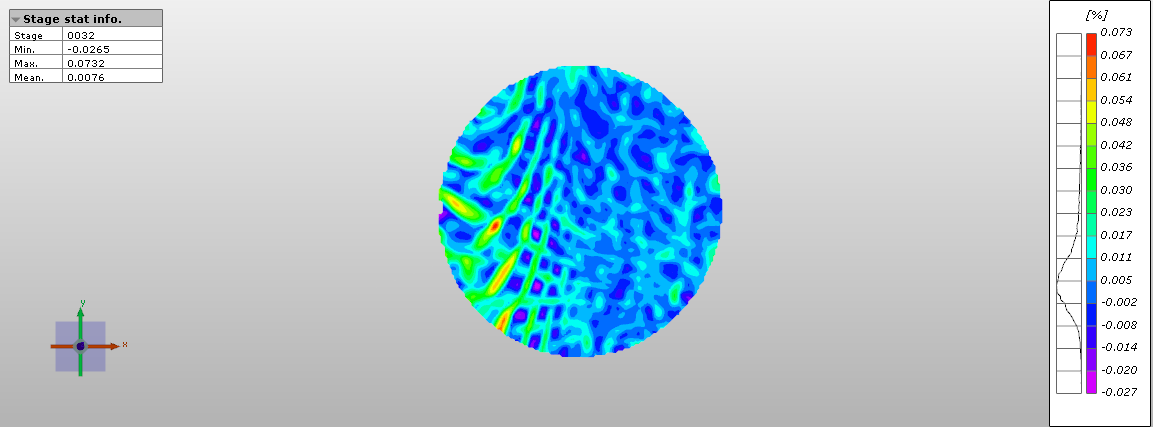

Supplement: S2 Data — (ZIP) [file pone.0294258.s002.zip › SNAPSERIES003/p0032.bmp]

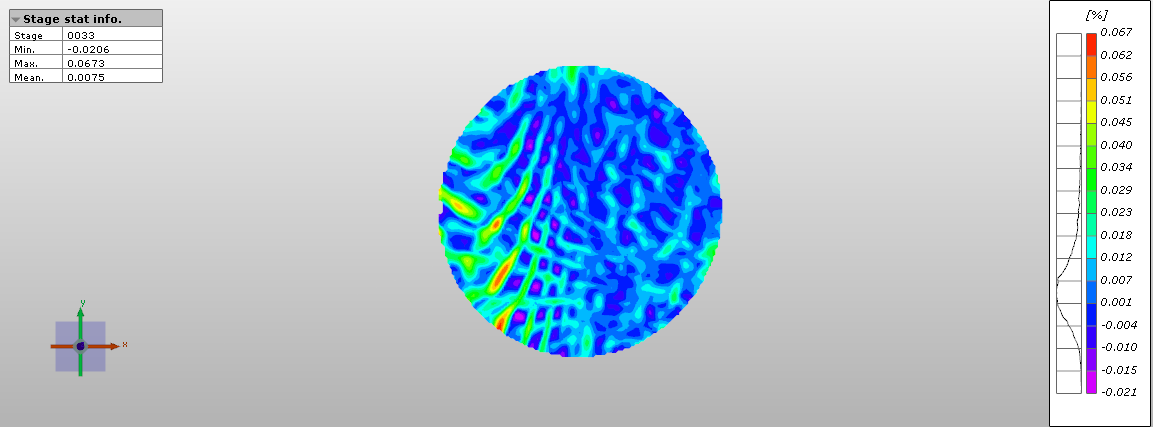

Supplement: S2 Data — (ZIP) [file pone.0294258.s002.zip › SNAPSERIES003/p0033.bmp]

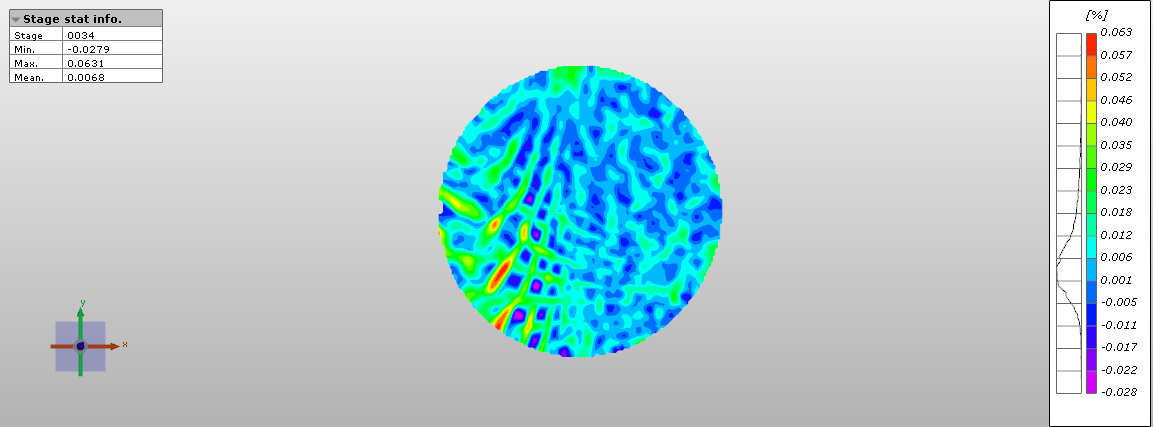

Supplement: S2 Data — (ZIP) [file pone.0294258.s002.zip › SNAPSERIES003/p0034.bmp]

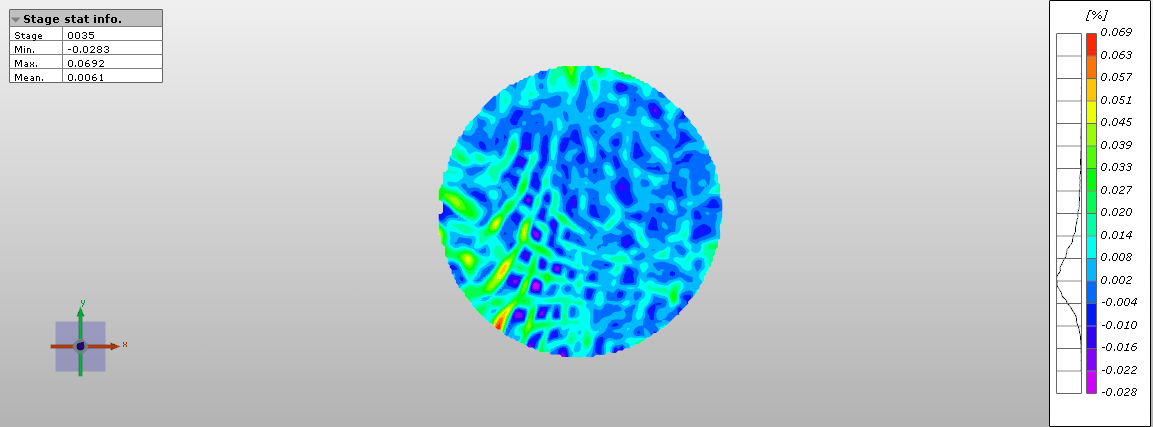

Supplement: S2 Data — (ZIP) [file pone.0294258.s002.zip › SNAPSERIES003/p0035.bmp]

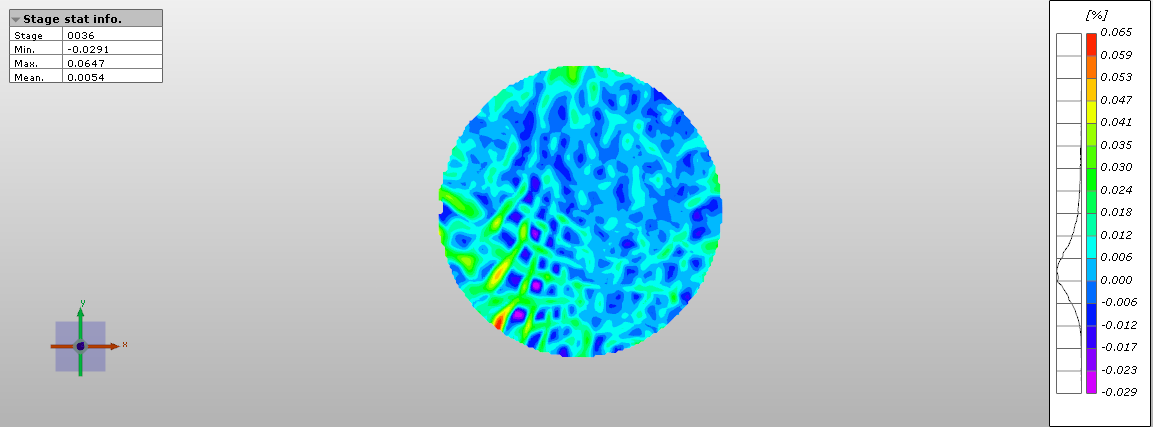

Supplement: S2 Data — (ZIP) [file pone.0294258.s002.zip › SNAPSERIES003/p0036.bmp]

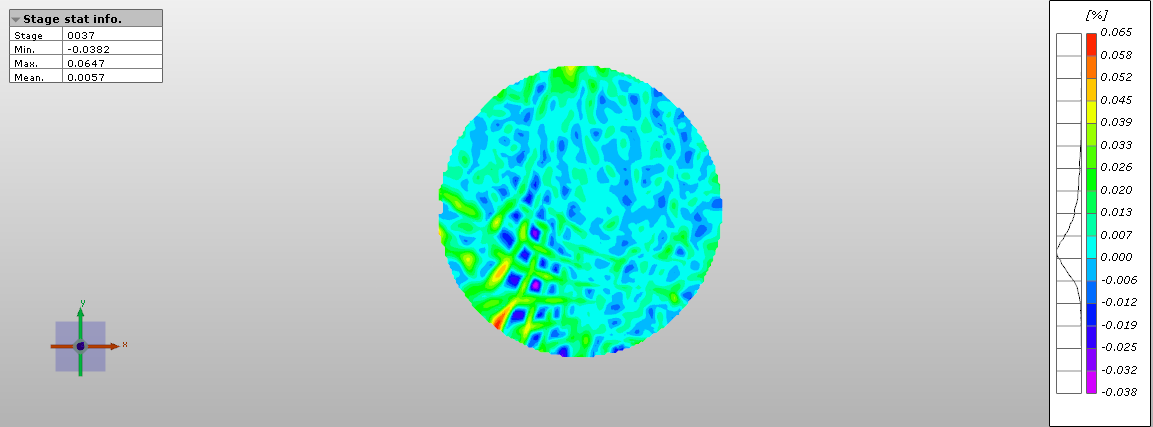

Supplement: S2 Data — (ZIP) [file pone.0294258.s002.zip › SNAPSERIES003/p0037.bmp]

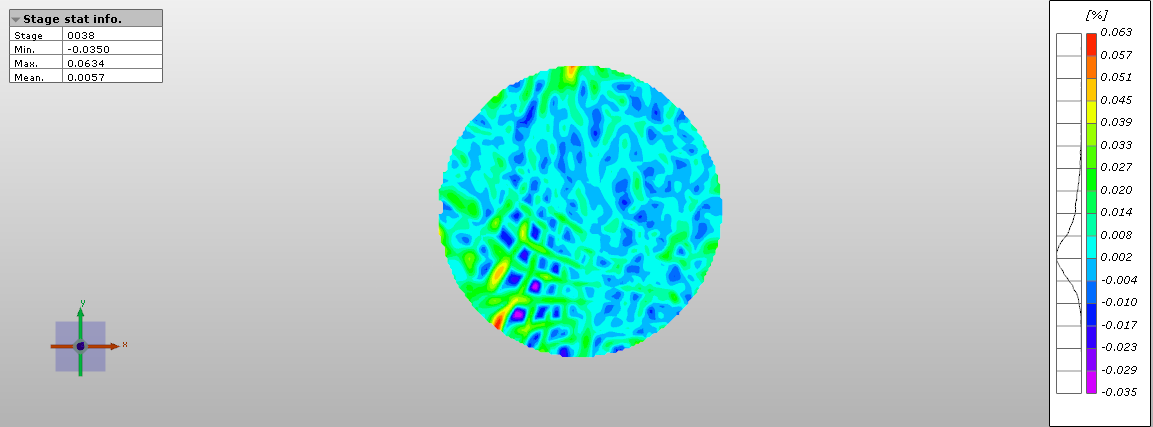

Supplement: S2 Data — (ZIP) [file pone.0294258.s002.zip › SNAPSERIES003/p0038.bmp]

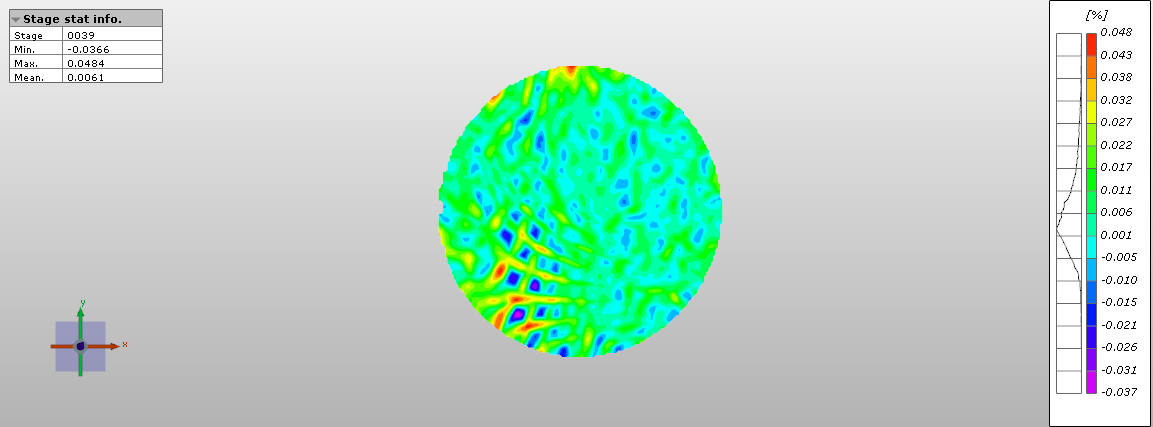

Supplement: S2 Data — (ZIP) [file pone.0294258.s002.zip › SNAPSERIES003/p0039.bmp]

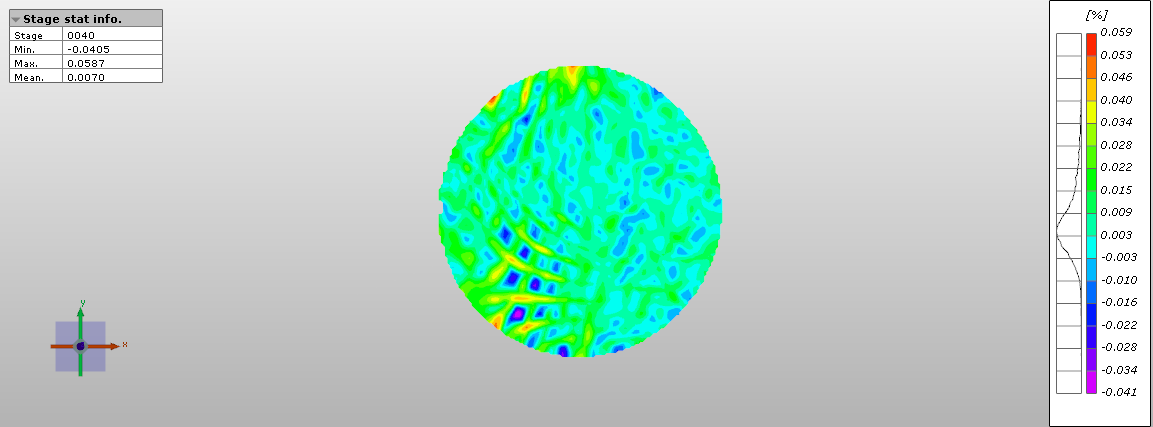

Supplement: S2 Data — (ZIP) [file pone.0294258.s002.zip › SNAPSERIES003/p0040.bmp]

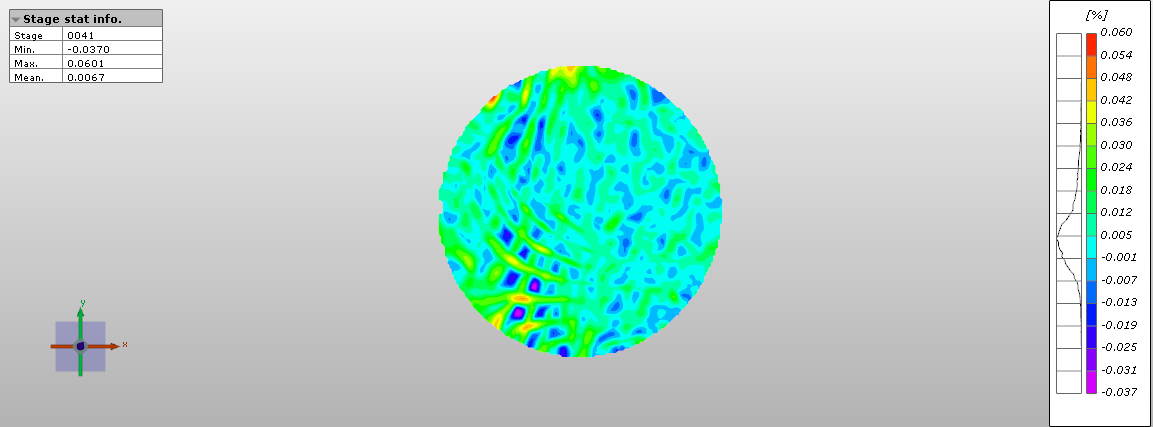

Supplement: S2 Data — (ZIP) [file pone.0294258.s002.zip › SNAPSERIES003/p0041.bmp]

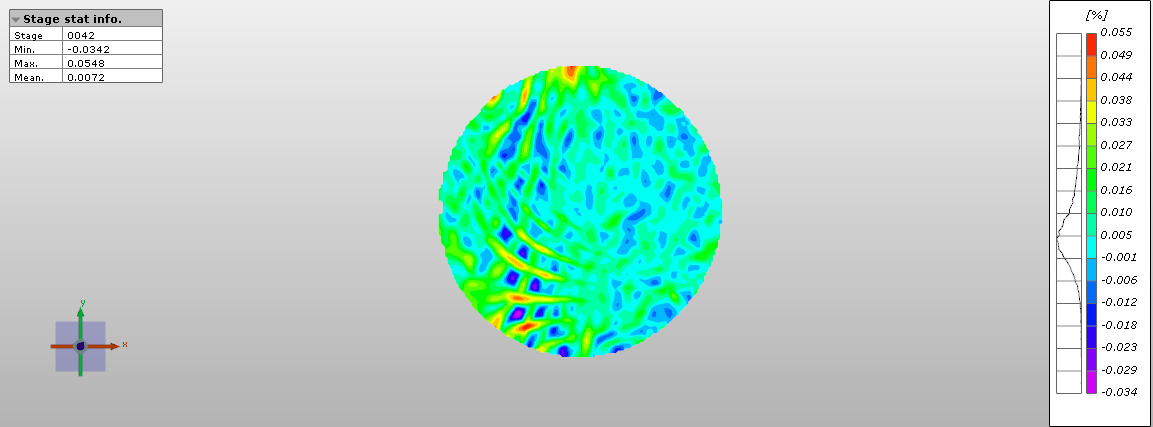

Supplement: S2 Data — (ZIP) [file pone.0294258.s002.zip › SNAPSERIES003/p0042.bmp]

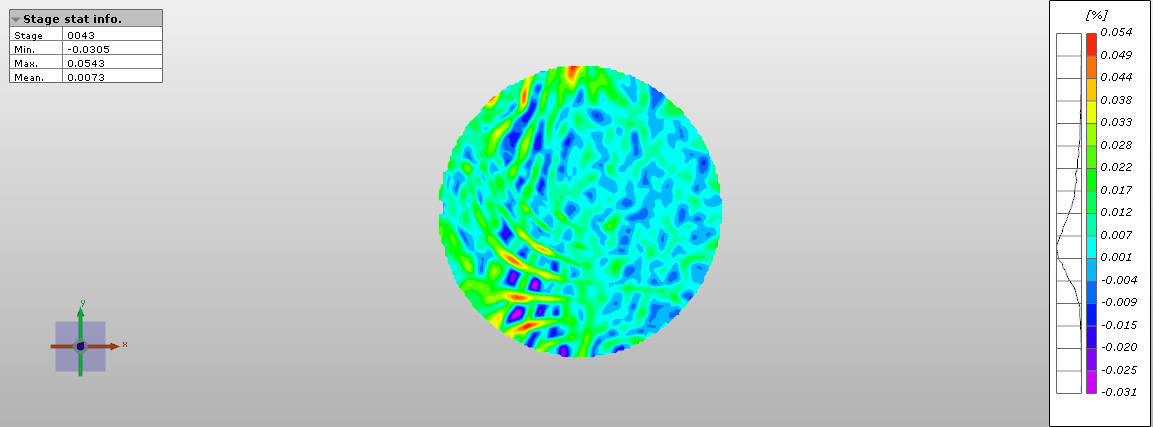

Supplement: S2 Data — (ZIP) [file pone.0294258.s002.zip › SNAPSERIES003/p0043.bmp]

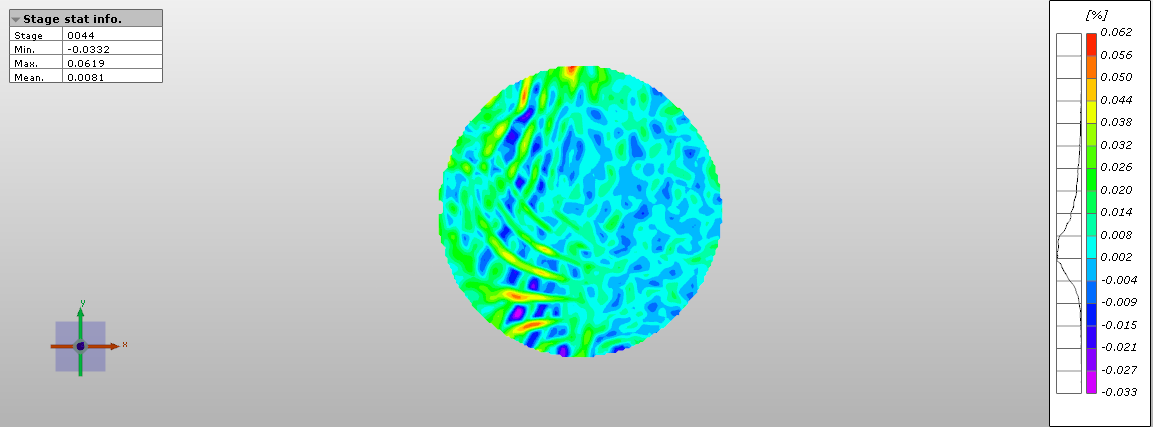

Supplement: S2 Data — (ZIP) [file pone.0294258.s002.zip › SNAPSERIES003/p0044.bmp]

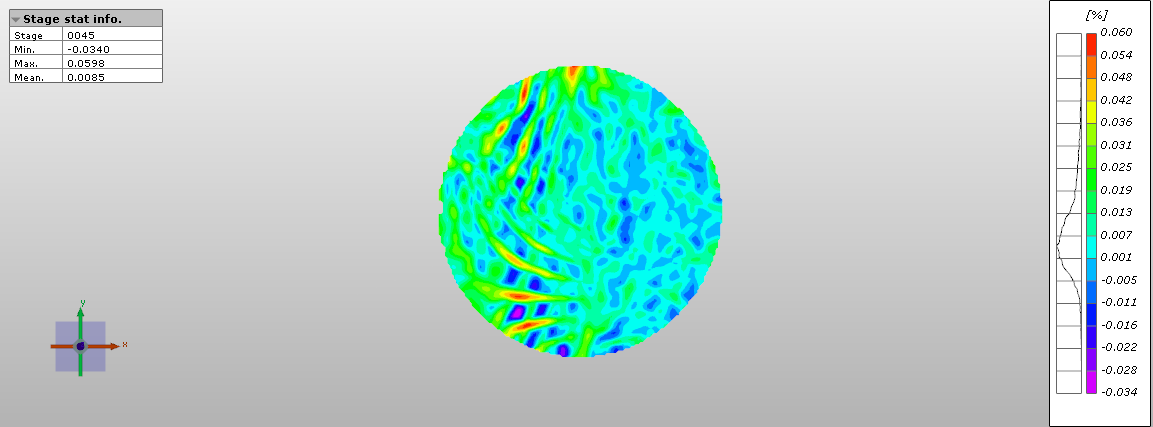

Supplement: S2 Data — (ZIP) [file pone.0294258.s002.zip › SNAPSERIES003/p0045.bmp]

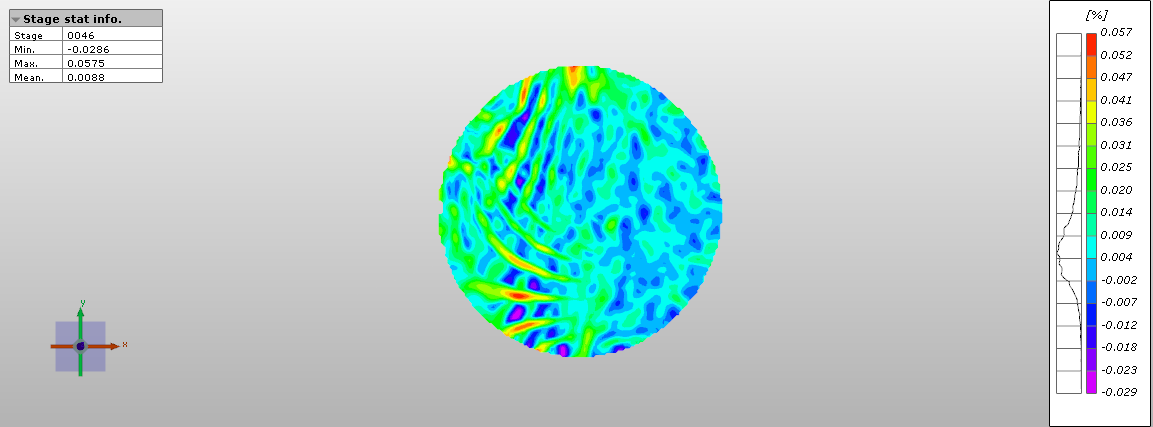

Supplement: S2 Data — (ZIP) [file pone.0294258.s002.zip › SNAPSERIES003/p0046.bmp]

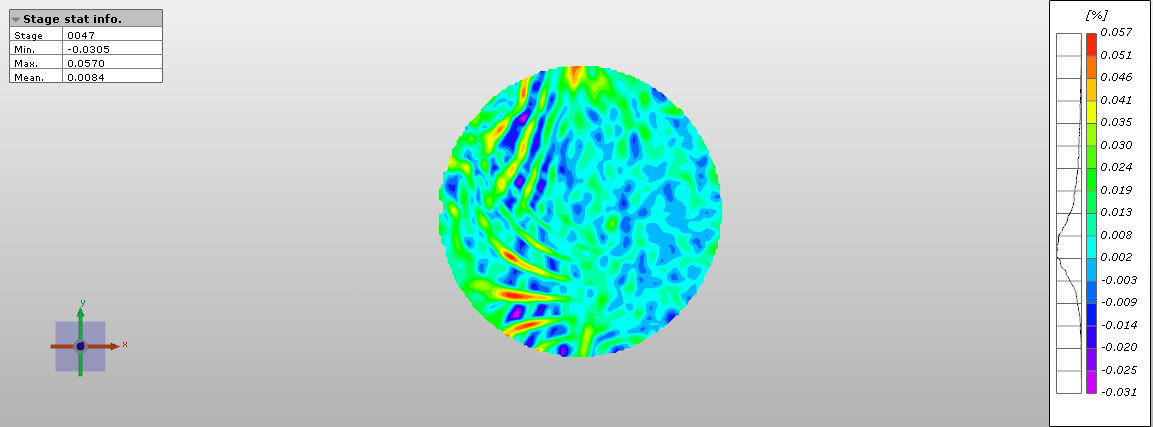

Supplement: S2 Data — (ZIP) [file pone.0294258.s002.zip › SNAPSERIES003/p0047.bmp]

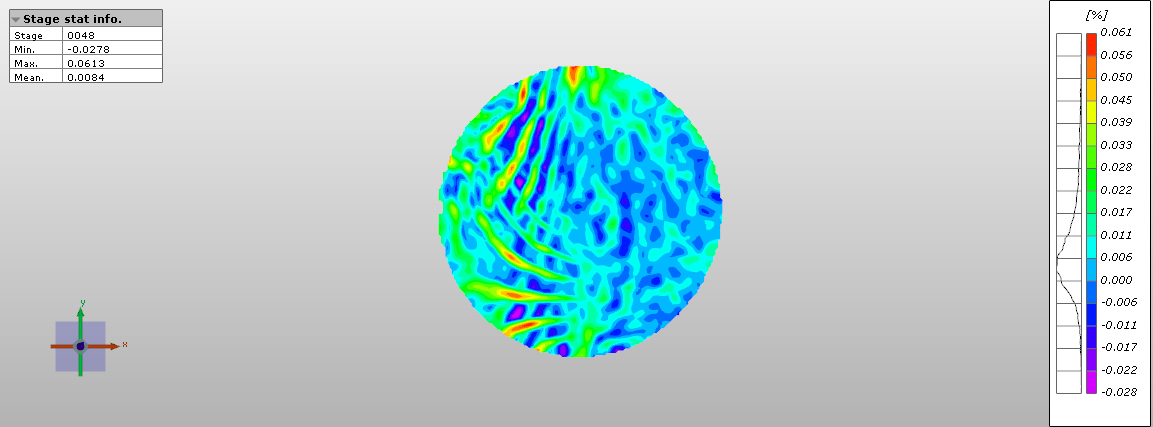

Supplement: S2 Data — (ZIP) [file pone.0294258.s002.zip › SNAPSERIES003/p0048.bmp]

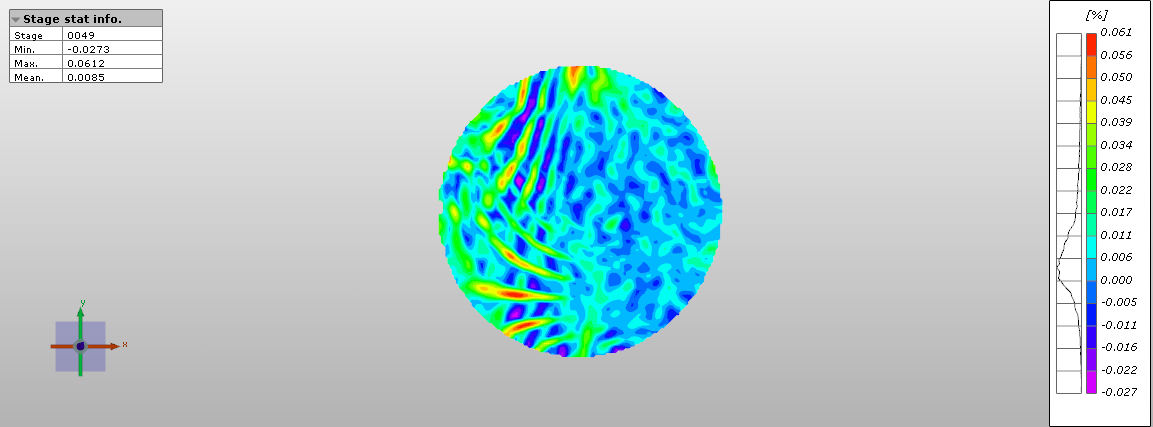

Supplement: S2 Data — (ZIP) [file pone.0294258.s002.zip › SNAPSERIES003/p0049.bmp]

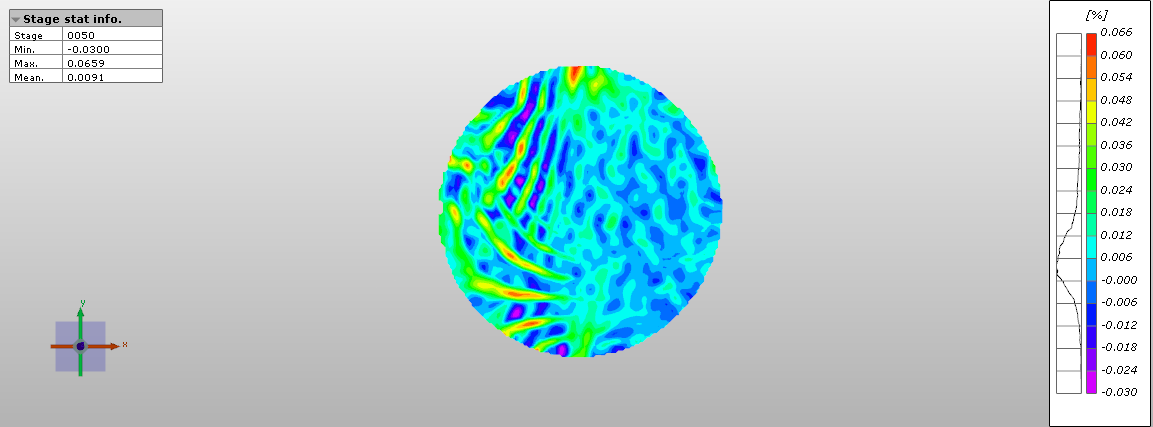

Supplement: S2 Data — (ZIP) [file pone.0294258.s002.zip › SNAPSERIES003/p0050.bmp]

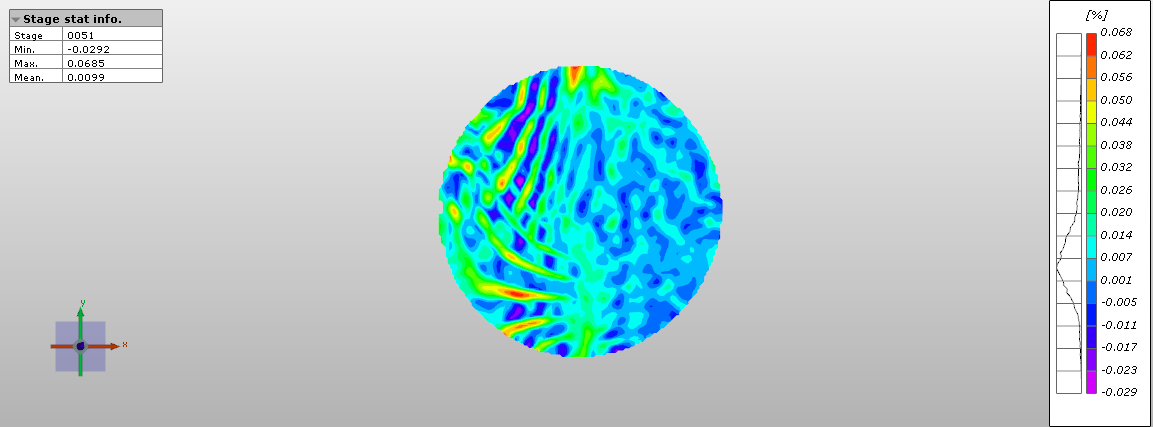

Supplement: S2 Data — (ZIP) [file pone.0294258.s002.zip › SNAPSERIES003/p0051.bmp]

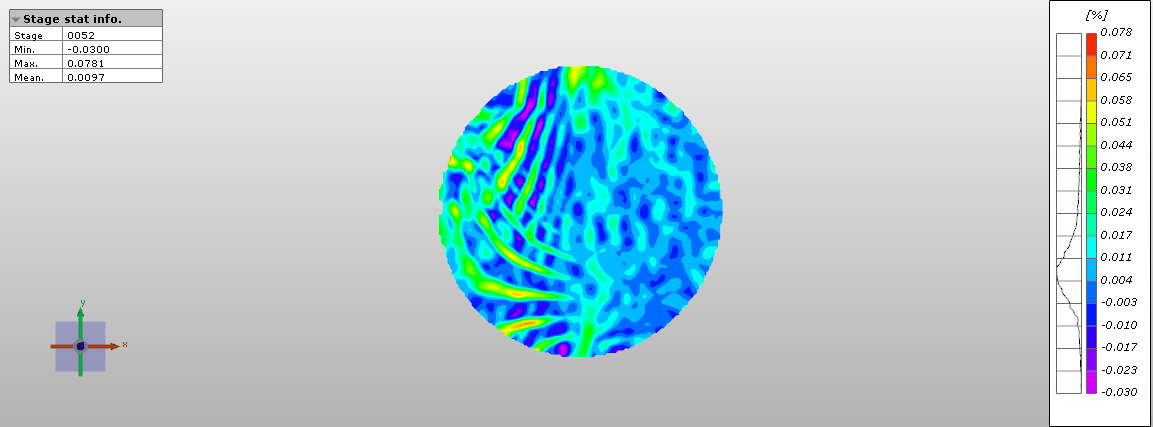

Supplement: S2 Data — (ZIP) [file pone.0294258.s002.zip › SNAPSERIES003/p0052.bmp]

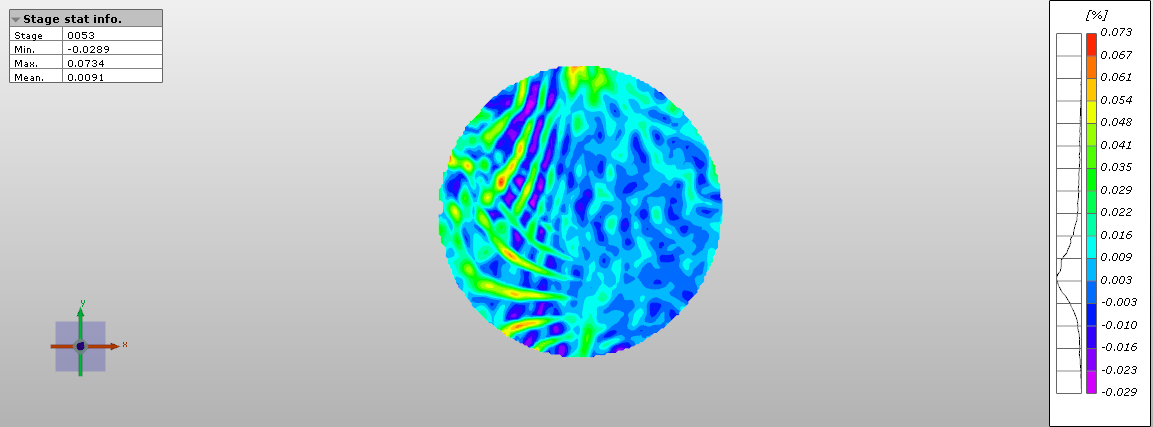

Supplement: S2 Data — (ZIP) [file pone.0294258.s002.zip › SNAPSERIES003/p0053.bmp]

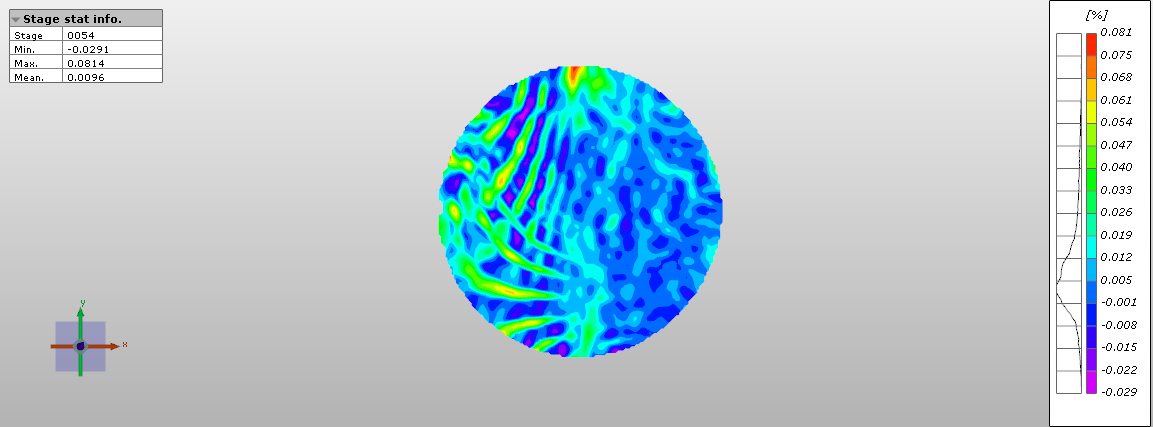

Supplement: S2 Data — (ZIP) [file pone.0294258.s002.zip › SNAPSERIES003/p0054.bmp]

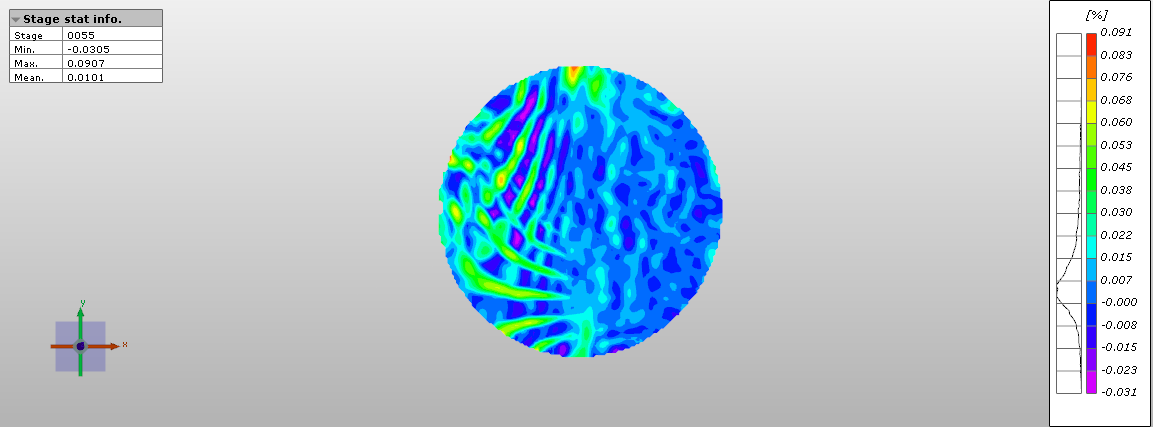

Supplement: S2 Data — (ZIP) [file pone.0294258.s002.zip › SNAPSERIES003/p0055.bmp]

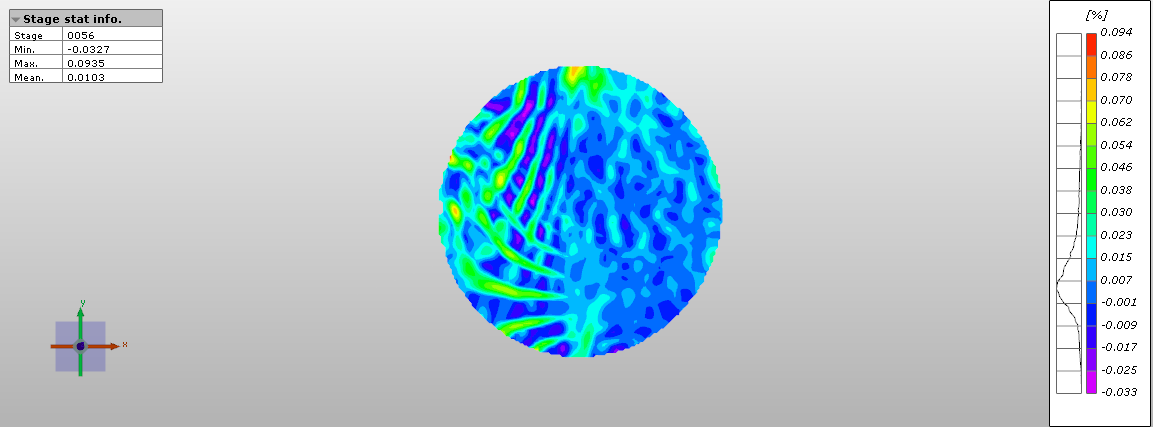

Supplement: S2 Data — (ZIP) [file pone.0294258.s002.zip › SNAPSERIES003/p0056.bmp]

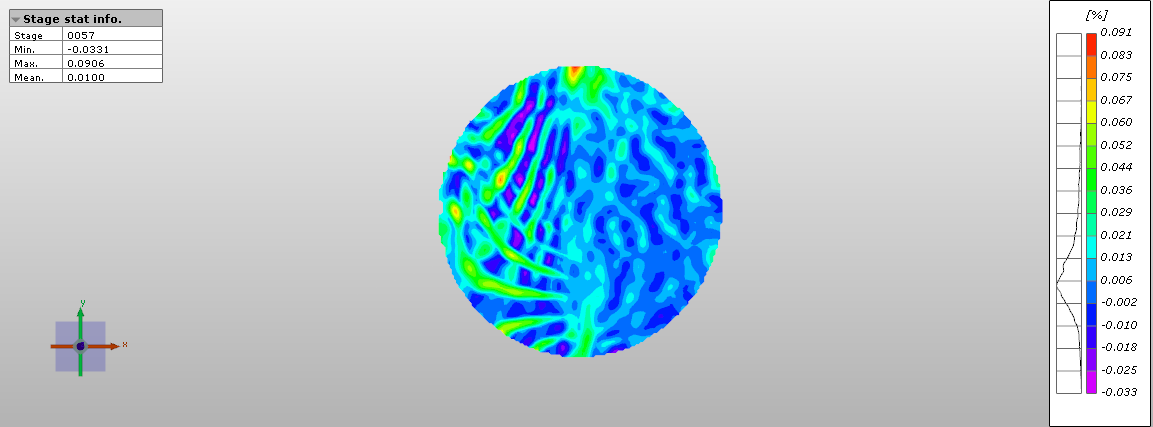

Supplement: S2 Data — (ZIP) [file pone.0294258.s002.zip › SNAPSERIES003/p0057.bmp]

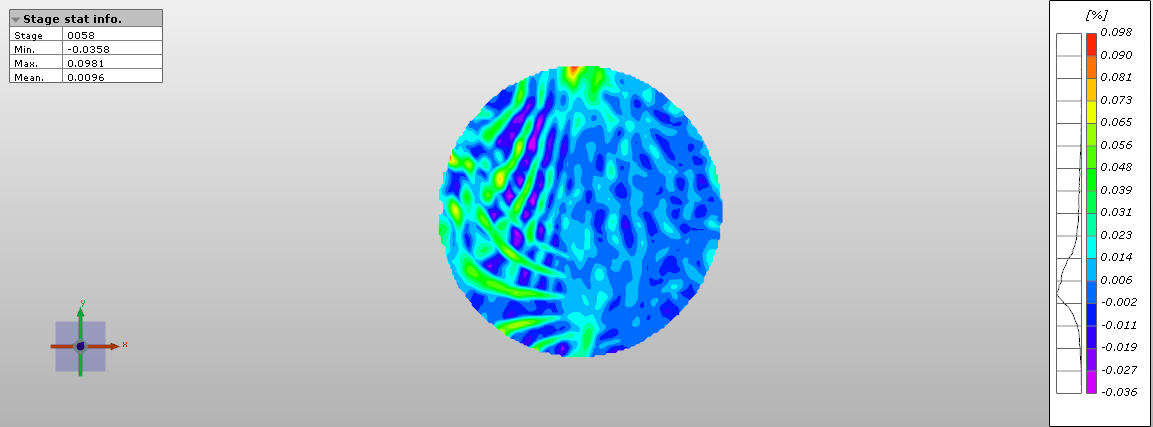

Supplement: S2 Data — (ZIP) [file pone.0294258.s002.zip › SNAPSERIES003/p0058.bmp]

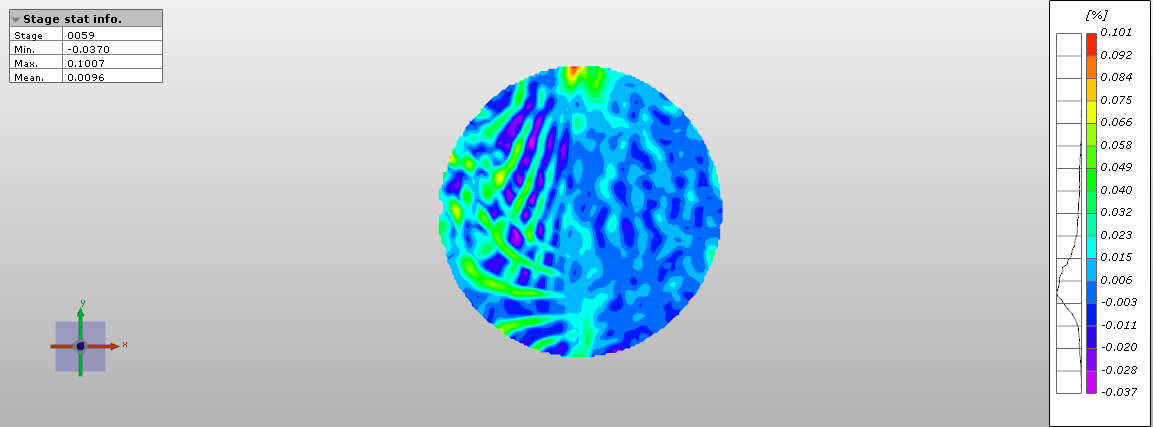

Supplement: S2 Data — (ZIP) [file pone.0294258.s002.zip › SNAPSERIES003/p0059.bmp]

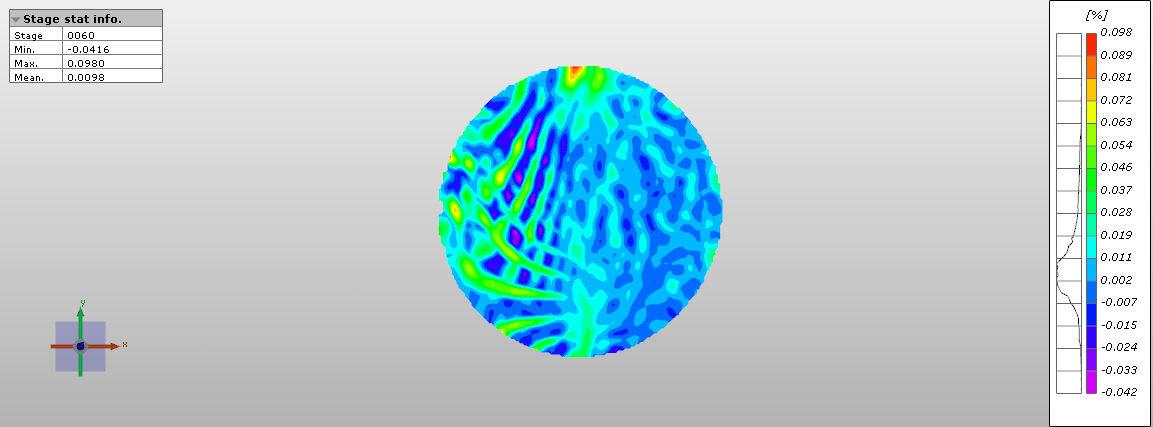

Supplement: S2 Data — (ZIP) [file pone.0294258.s002.zip › SNAPSERIES003/p0060.bmp]

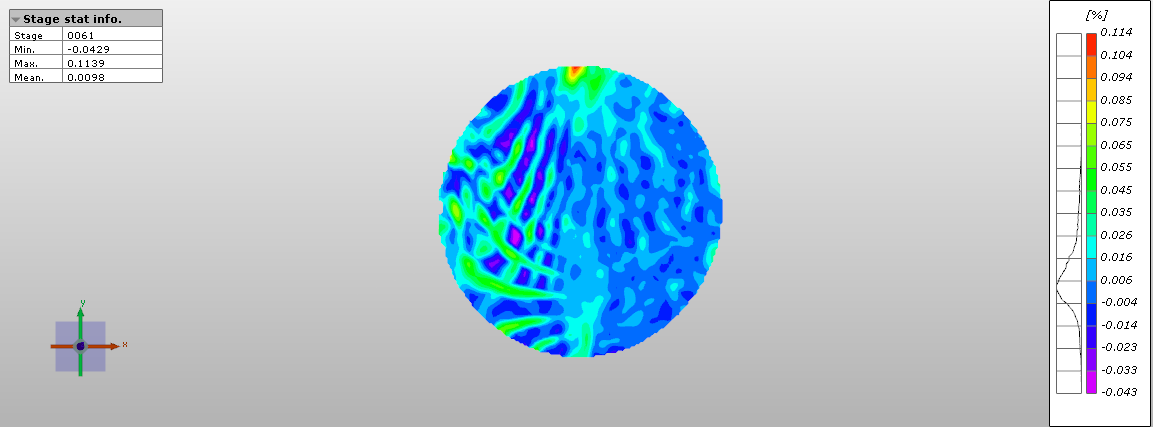

Supplement: S2 Data — (ZIP) [file pone.0294258.s002.zip › SNAPSERIES003/p0061.bmp]

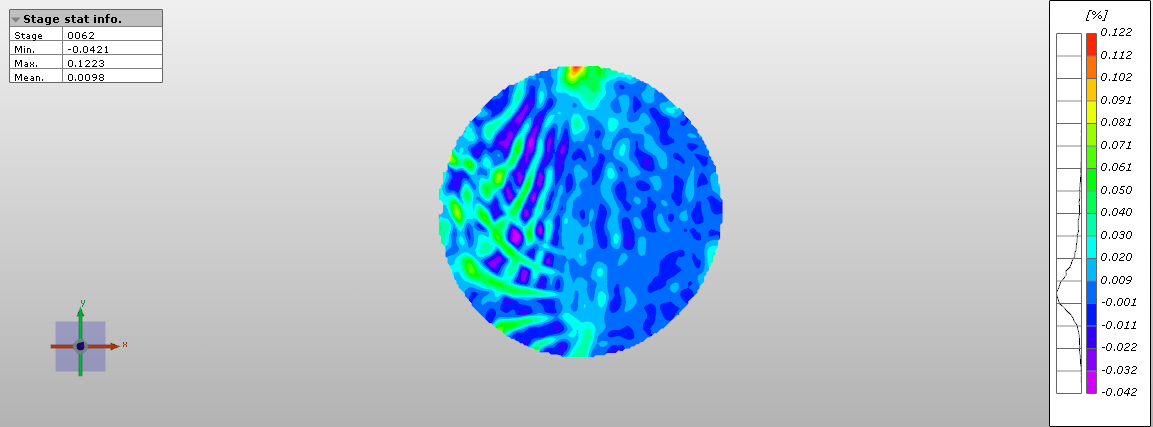

Supplement: S2 Data — (ZIP) [file pone.0294258.s002.zip › SNAPSERIES003/p0062.bmp]

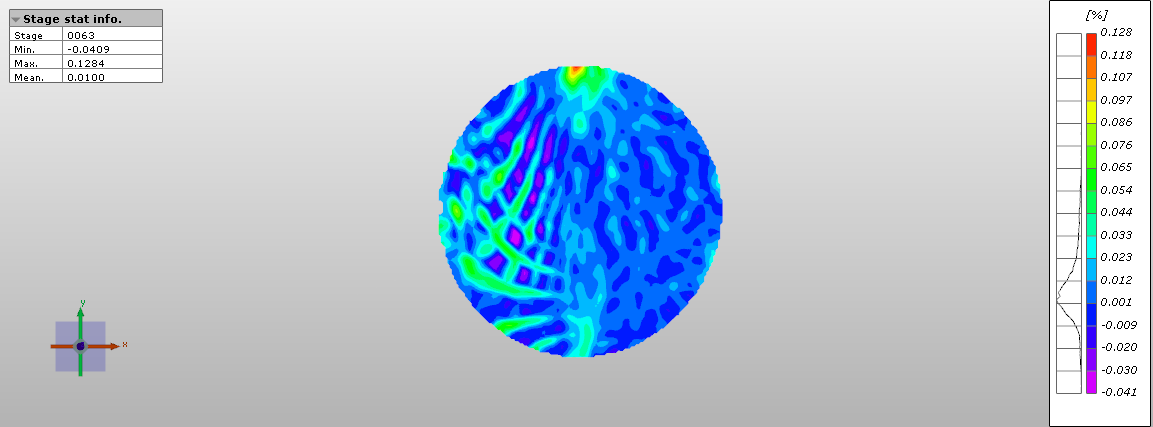

Supplement: S2 Data — (ZIP) [file pone.0294258.s002.zip › SNAPSERIES003/p0063.bmp]

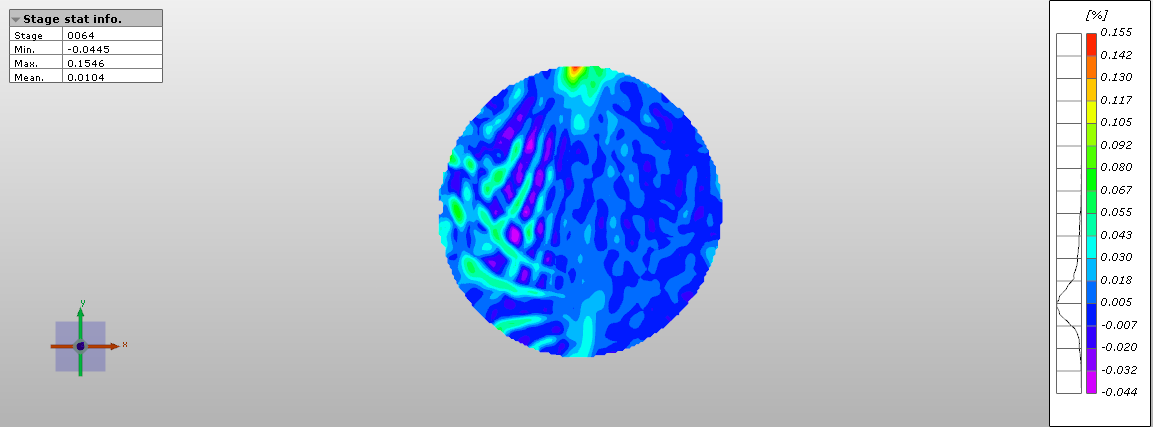

Supplement: S2 Data — (ZIP) [file pone.0294258.s002.zip › SNAPSERIES003/p0064.bmp]

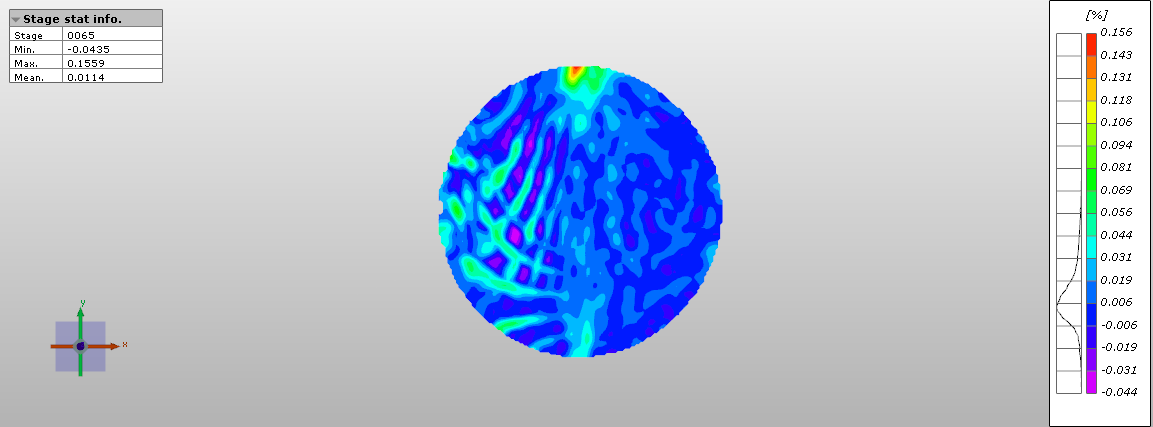

Supplement: S2 Data — (ZIP) [file pone.0294258.s002.zip › SNAPSERIES003/p0065.bmp]

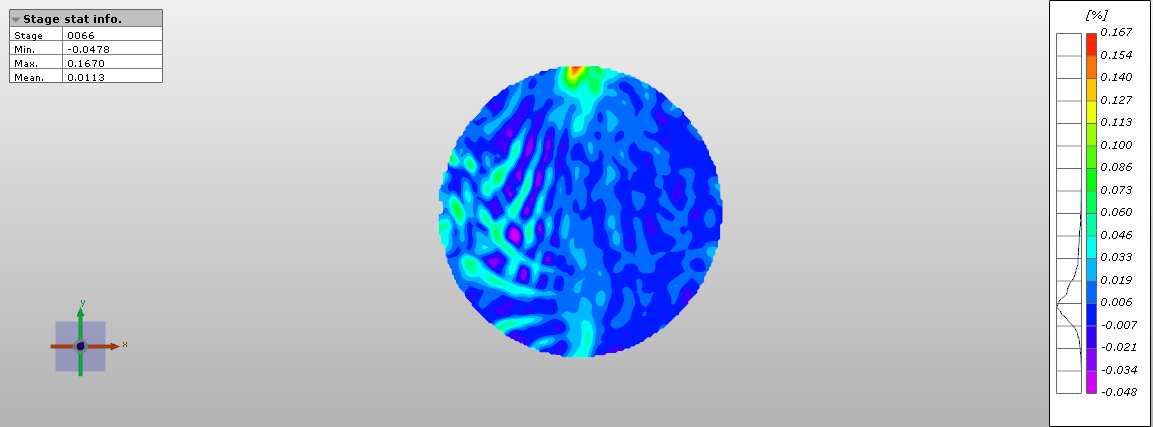

Supplement: S2 Data — (ZIP) [file pone.0294258.s002.zip › SNAPSERIES003/p0066.bmp]

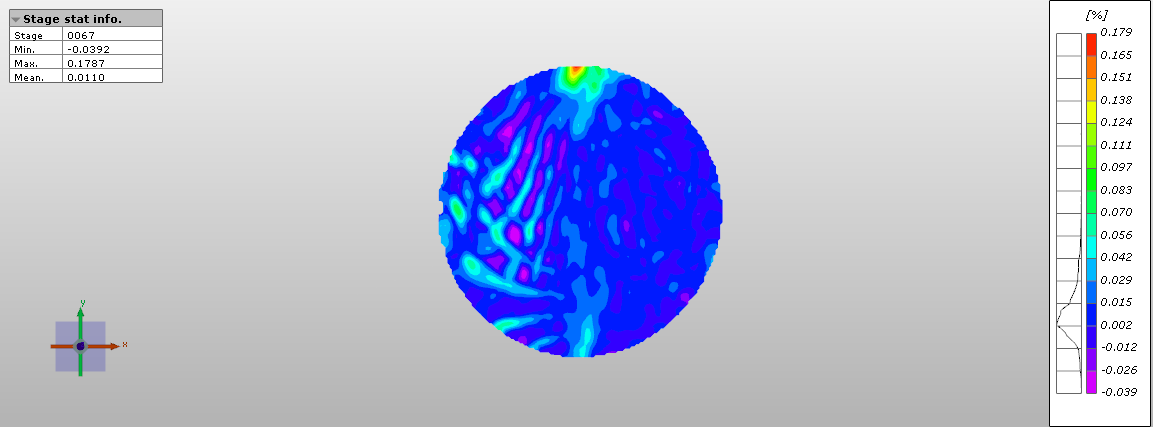

Supplement: S2 Data — (ZIP) [file pone.0294258.s002.zip › SNAPSERIES003/p0067.bmp]

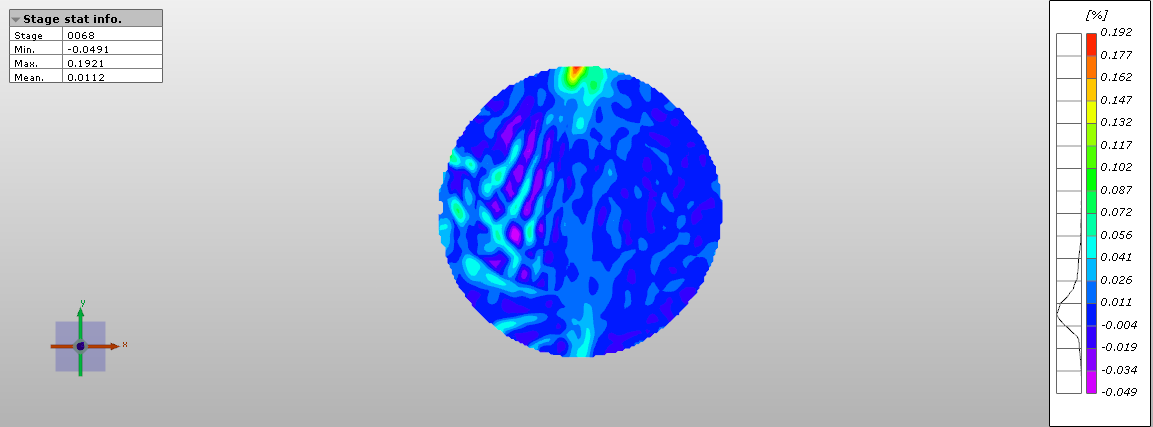

Supplement: S2 Data — (ZIP) [file pone.0294258.s002.zip › SNAPSERIES003/p0068.bmp]

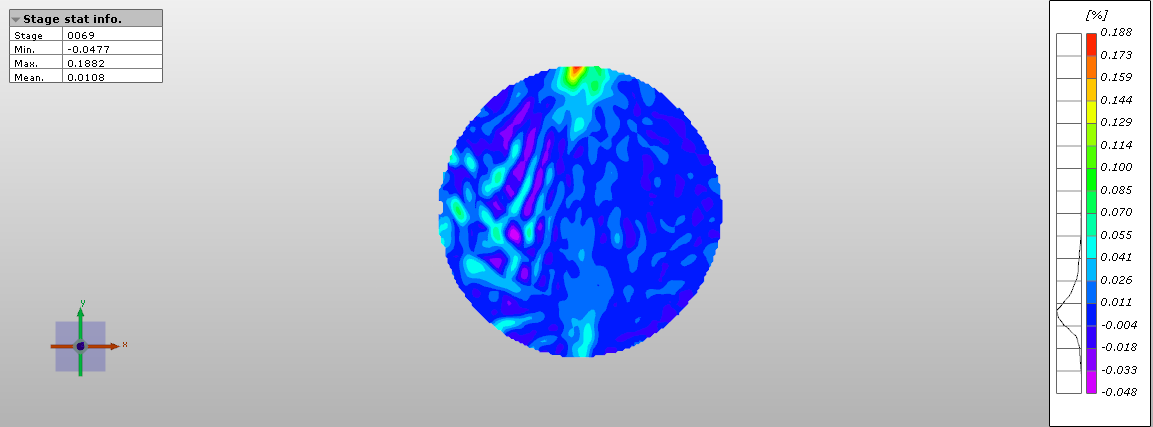

Supplement: S2 Data — (ZIP) [file pone.0294258.s002.zip › SNAPSERIES003/p0069.bmp]

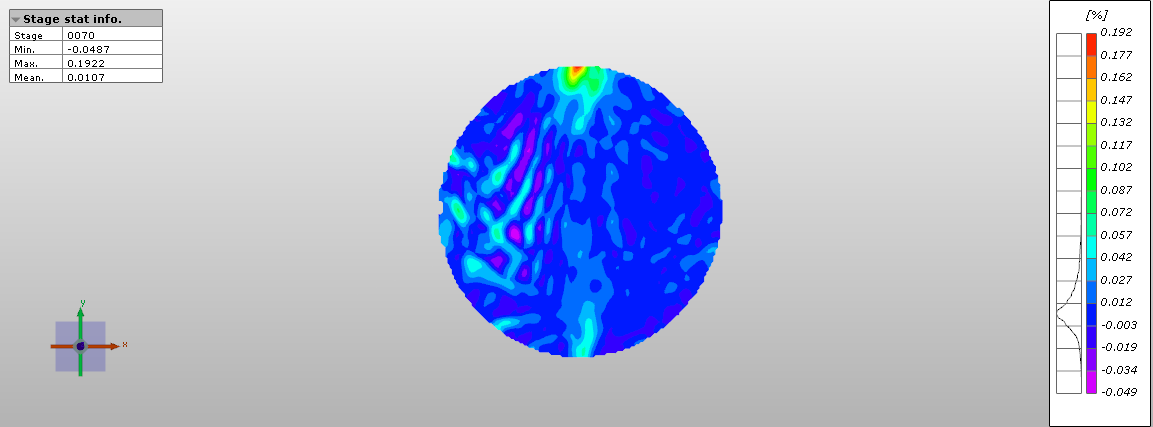

Supplement: S2 Data — (ZIP) [file pone.0294258.s002.zip › SNAPSERIES003/p0070.bmp]

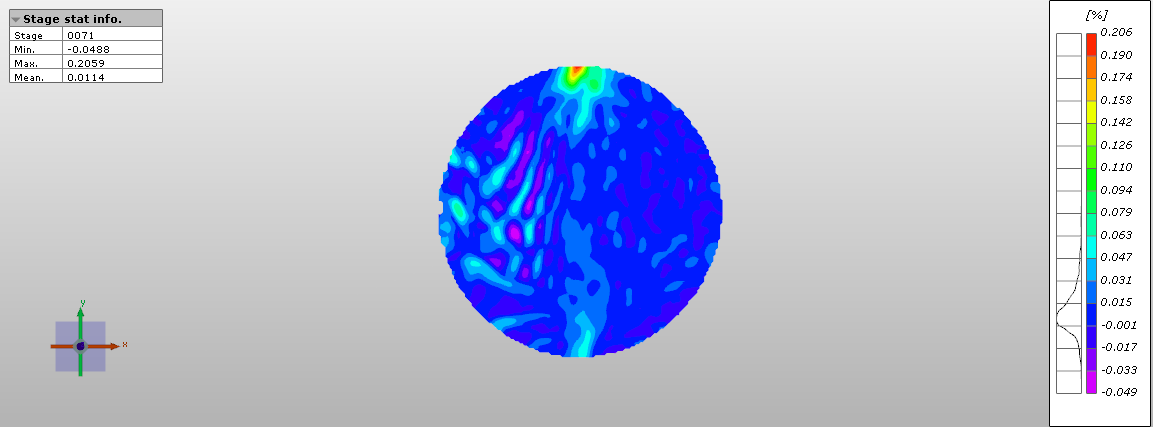

Supplement: S2 Data — (ZIP) [file pone.0294258.s002.zip › SNAPSERIES003/p0071.bmp]

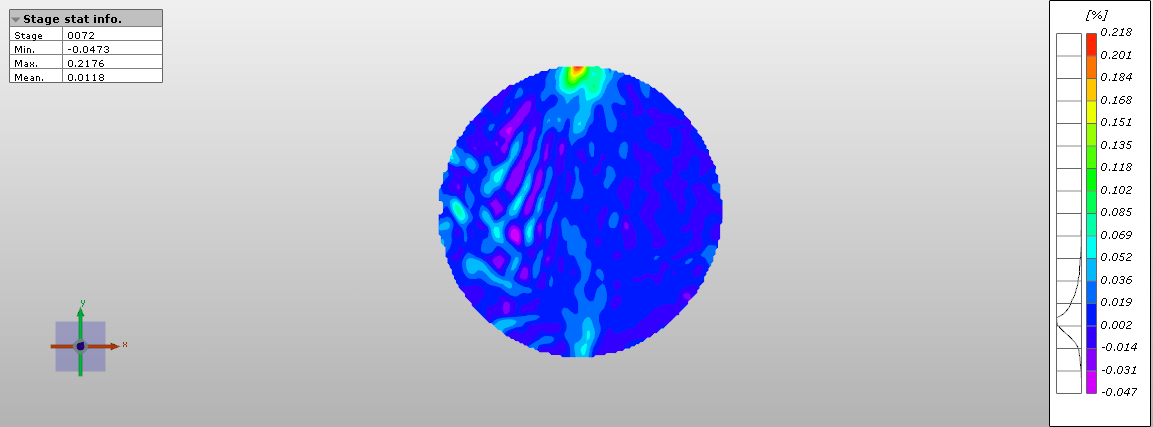

Supplement: S2 Data — (ZIP) [file pone.0294258.s002.zip › SNAPSERIES003/p0072.bmp]

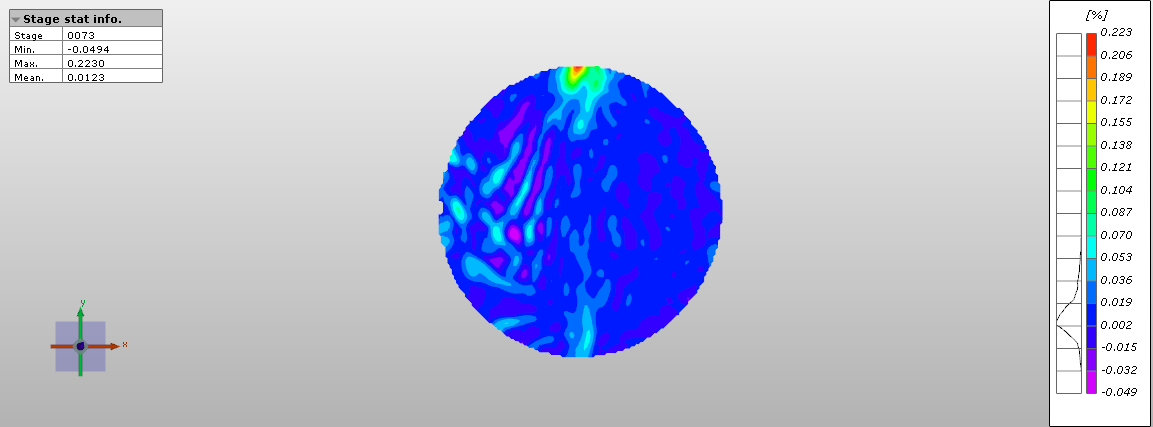

Supplement: S2 Data — (ZIP) [file pone.0294258.s002.zip › SNAPSERIES003/p0073.bmp]

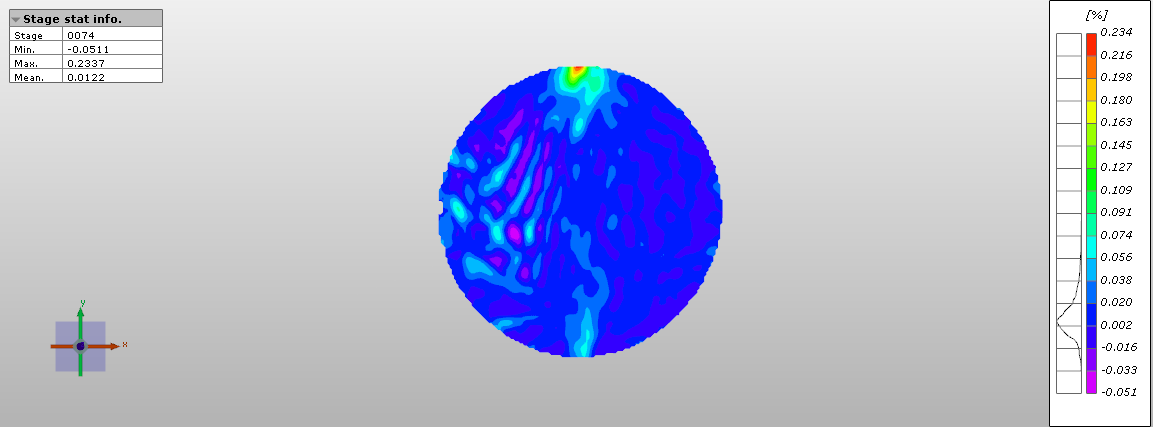

Supplement: S2 Data — (ZIP) [file pone.0294258.s002.zip › SNAPSERIES003/p0074.bmp]

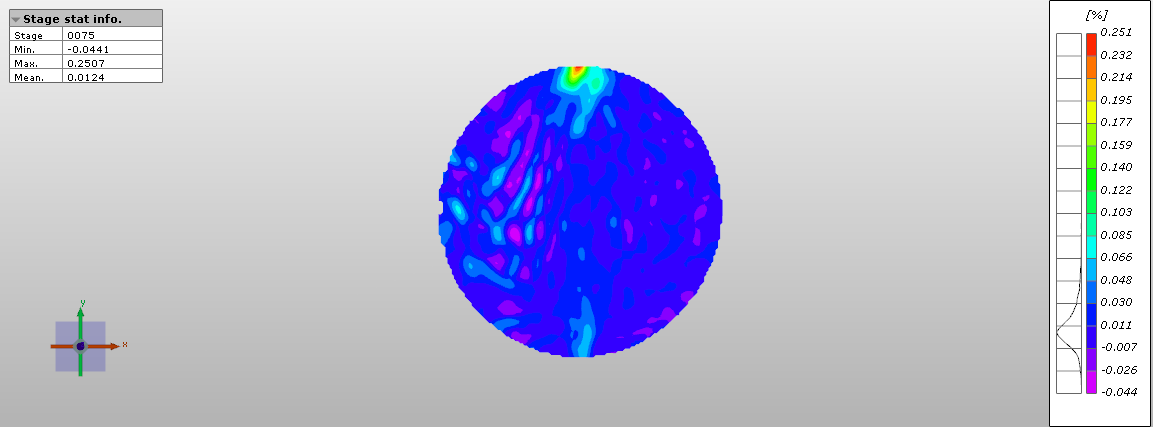

Supplement: S2 Data — (ZIP) [file pone.0294258.s002.zip › SNAPSERIES003/p0075.bmp]

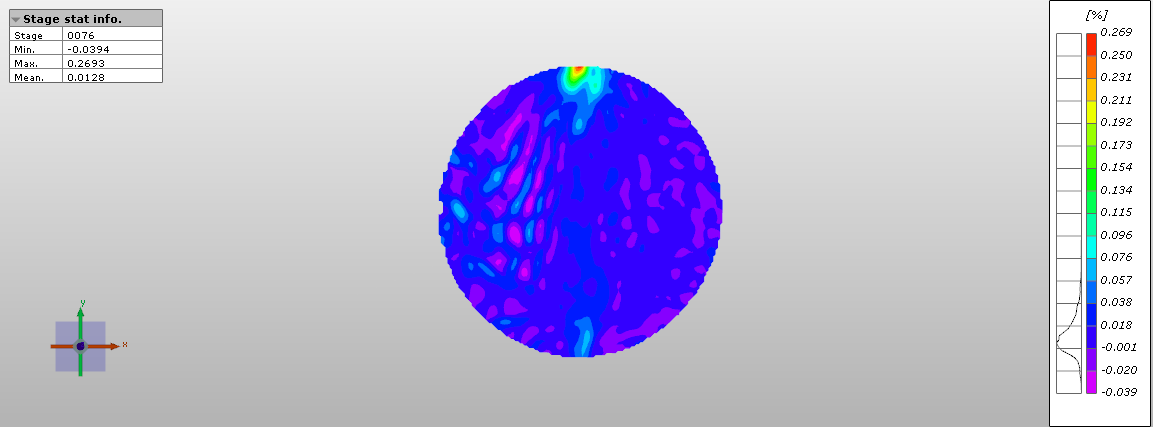

Supplement: S2 Data — (ZIP) [file pone.0294258.s002.zip › SNAPSERIES003/p0076.bmp]

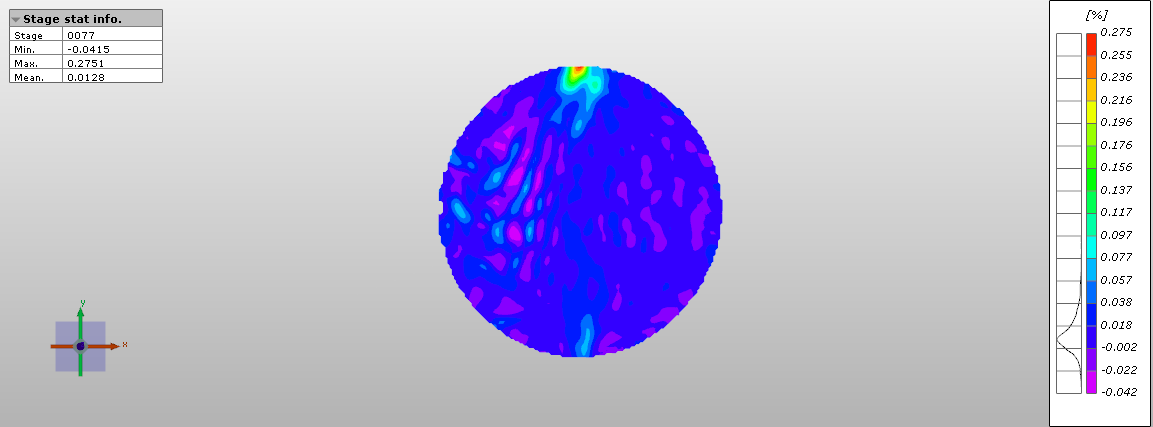

Supplement: S2 Data — (ZIP) [file pone.0294258.s002.zip › SNAPSERIES003/p0077.bmp]

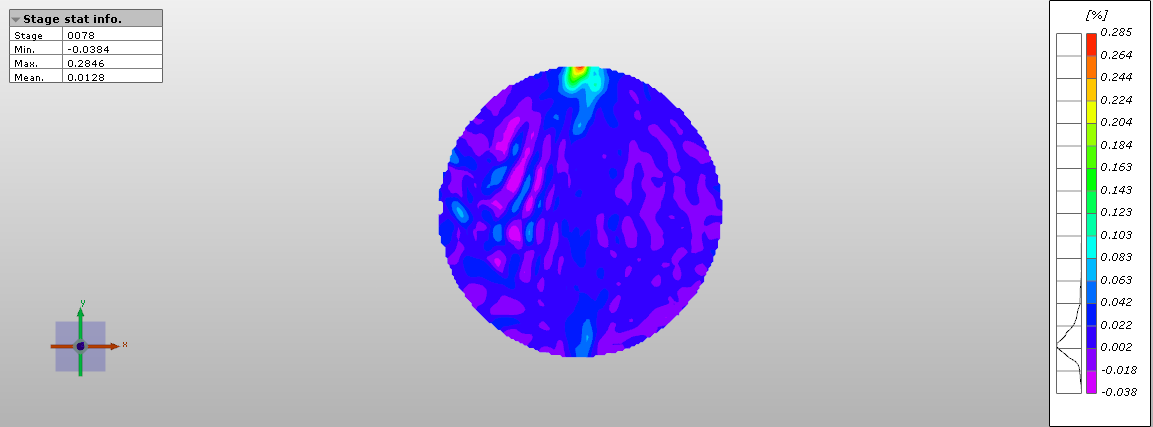

Supplement: S2 Data — (ZIP) [file pone.0294258.s002.zip › SNAPSERIES003/p0078.bmp]

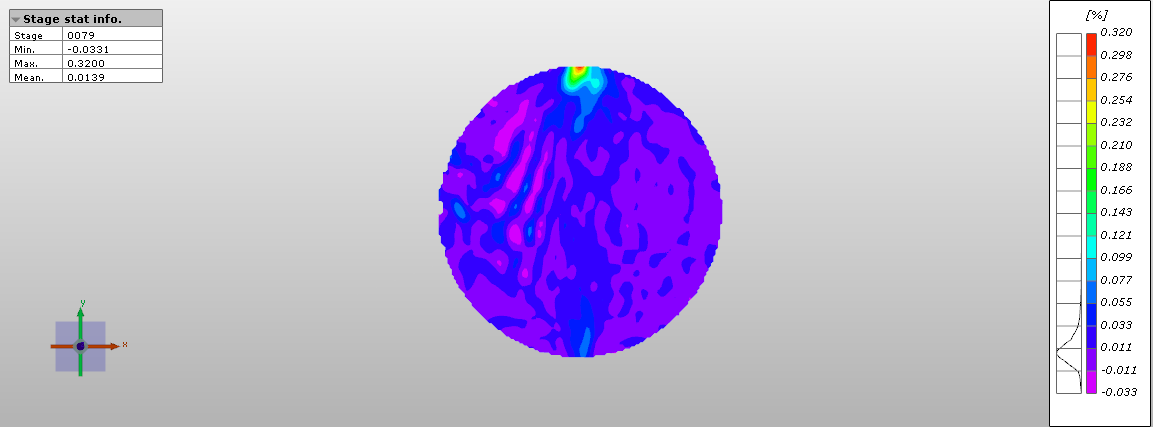

Supplement: S2 Data — (ZIP) [file pone.0294258.s002.zip › SNAPSERIES003/p0079.bmp]

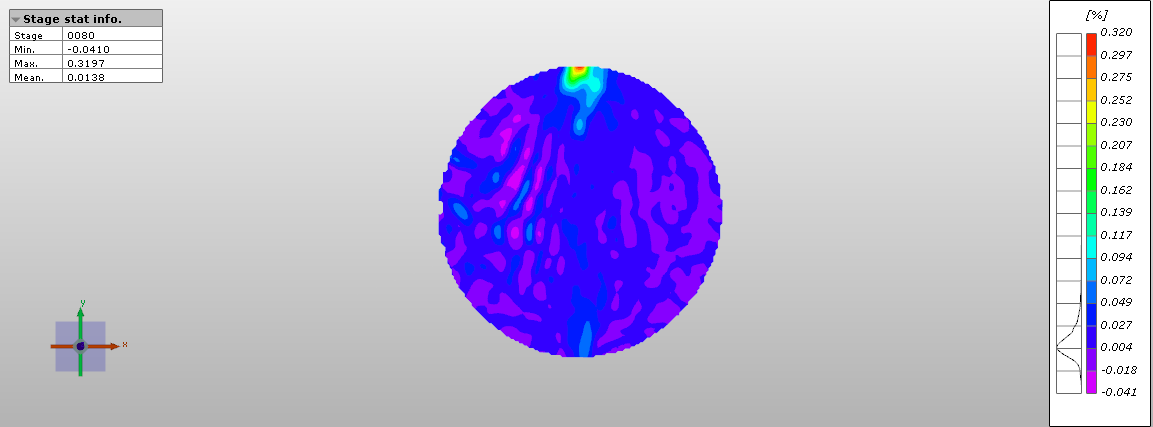

Supplement: S2 Data — (ZIP) [file pone.0294258.s002.zip › SNAPSERIES003/p0080.bmp]

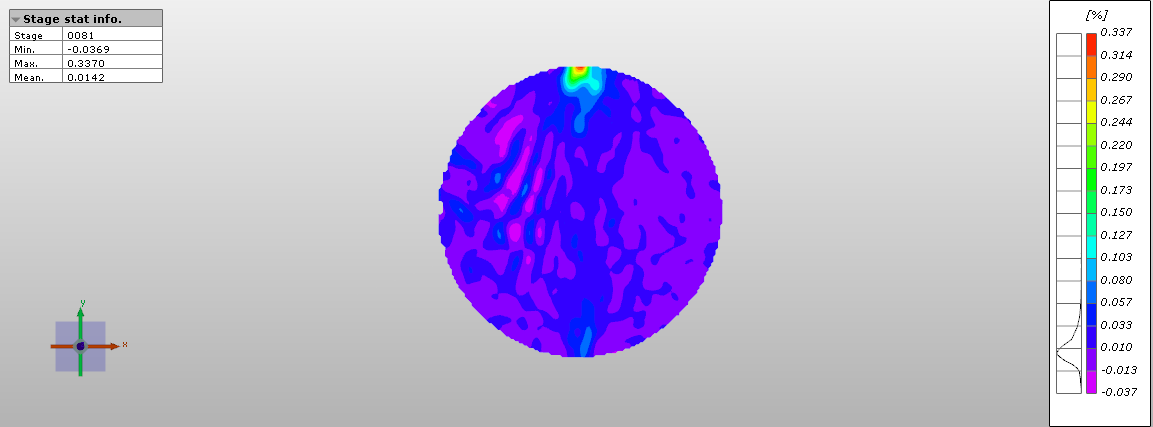

Supplement: S2 Data — (ZIP) [file pone.0294258.s002.zip › SNAPSERIES003/p0081.bmp]

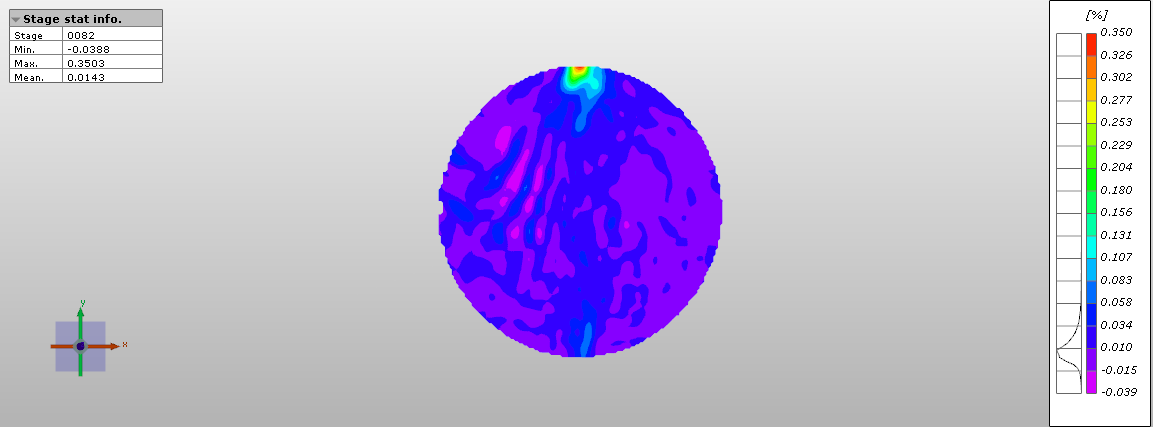

Supplement: S2 Data — (ZIP) [file pone.0294258.s002.zip › SNAPSERIES003/p0082.bmp]

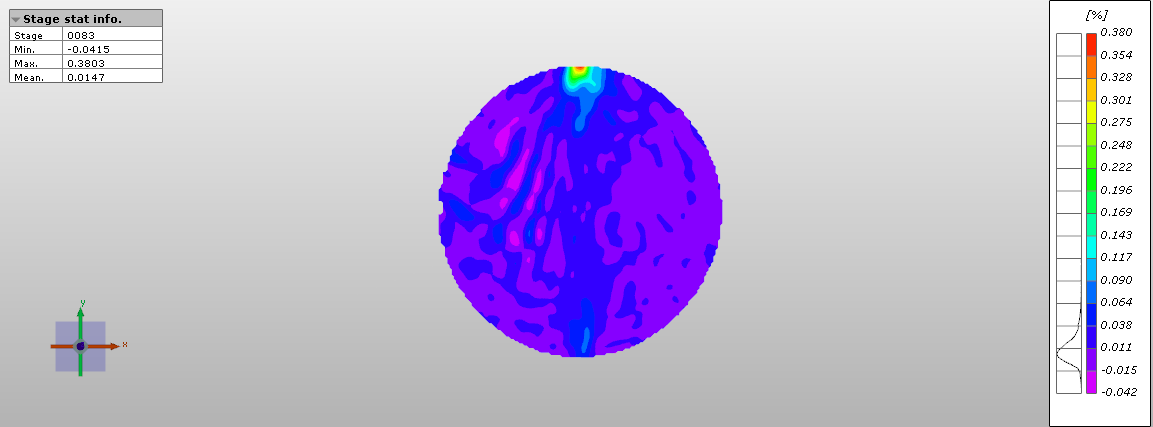

Supplement: S2 Data — (ZIP) [file pone.0294258.s002.zip › SNAPSERIES003/p0083.bmp]

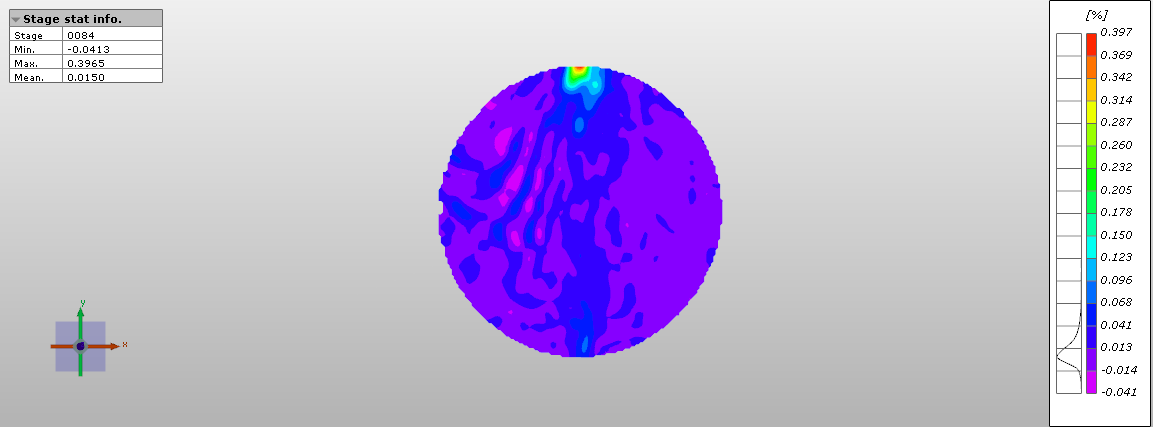

Supplement: S2 Data — (ZIP) [file pone.0294258.s002.zip › SNAPSERIES003/p0084.bmp]

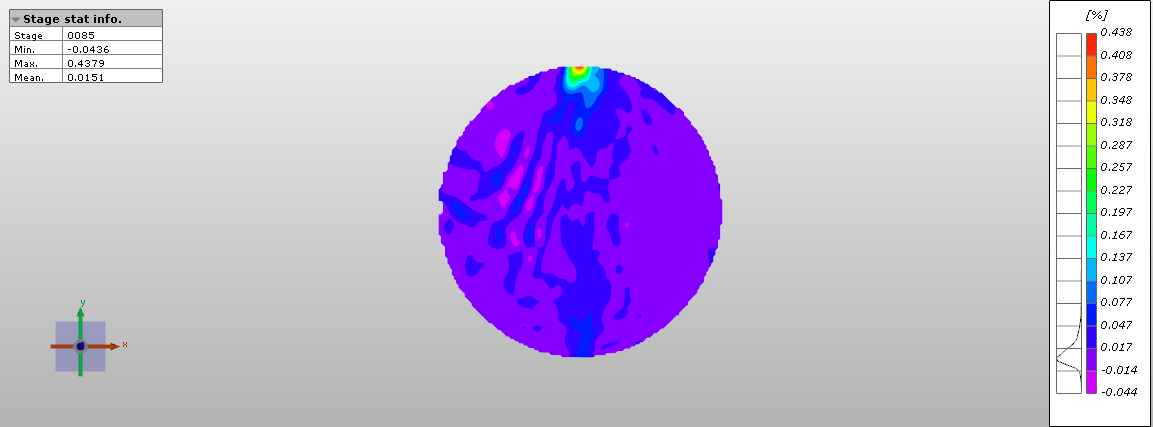

Supplement: S2 Data — (ZIP) [file pone.0294258.s002.zip › SNAPSERIES003/p0085.bmp]

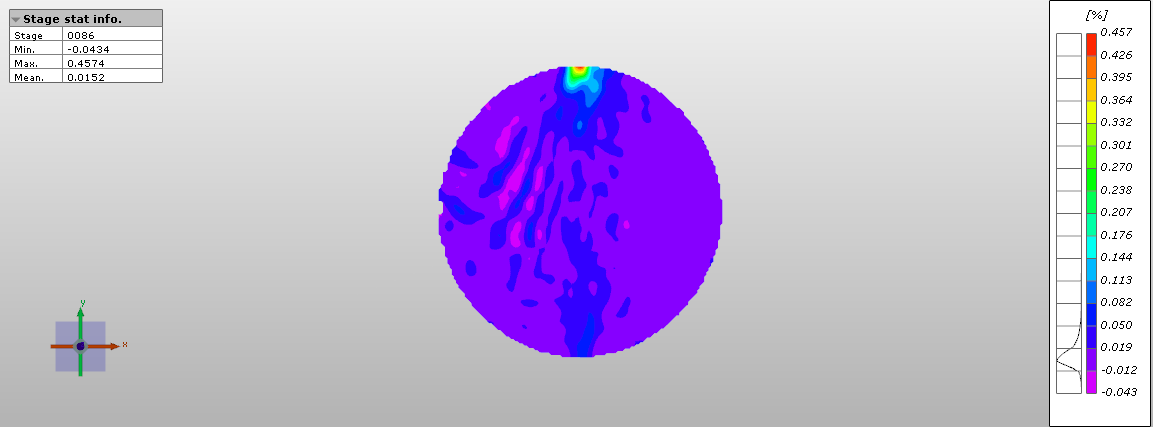

Supplement: S2 Data — (ZIP) [file pone.0294258.s002.zip › SNAPSERIES003/p0086.bmp]

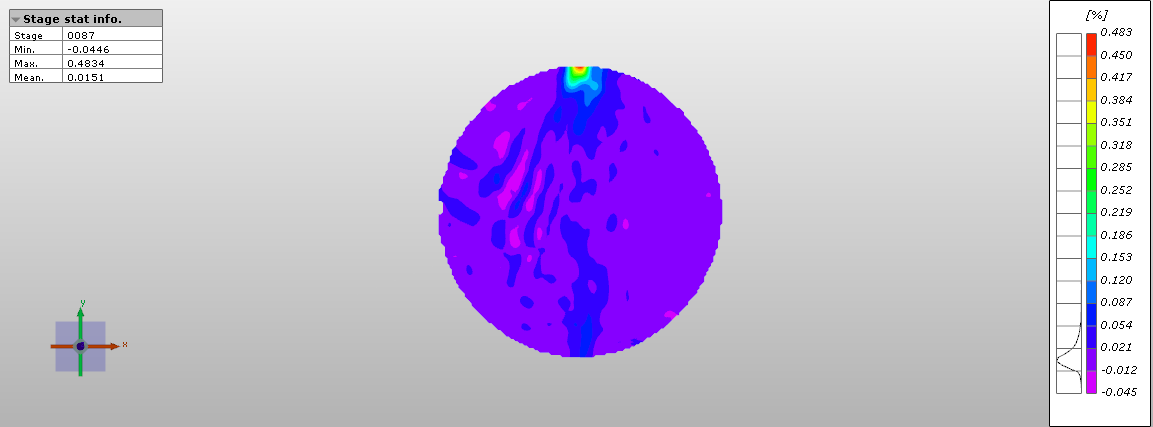

Supplement: S2 Data — (ZIP) [file pone.0294258.s002.zip › SNAPSERIES003/p0087.bmp]

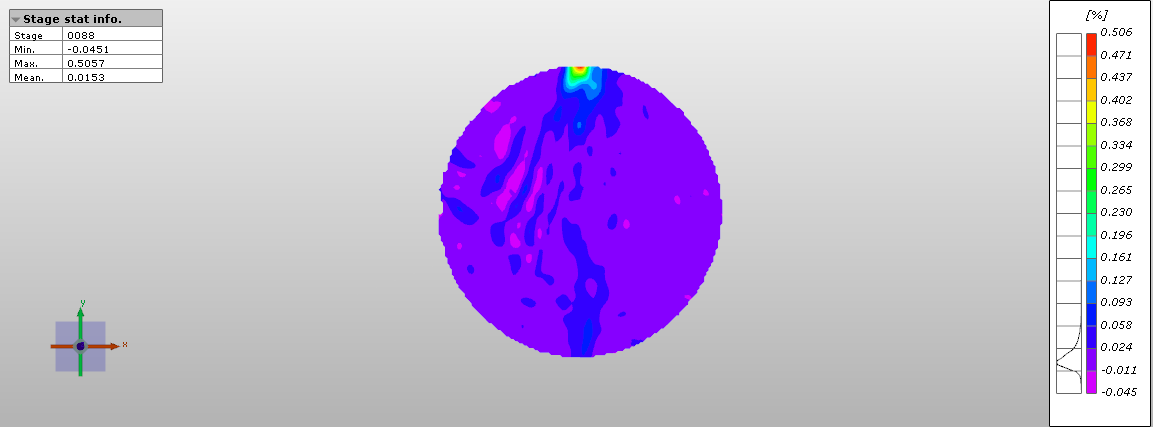

Supplement: S2 Data — (ZIP) [file pone.0294258.s002.zip › SNAPSERIES003/p0088.bmp]

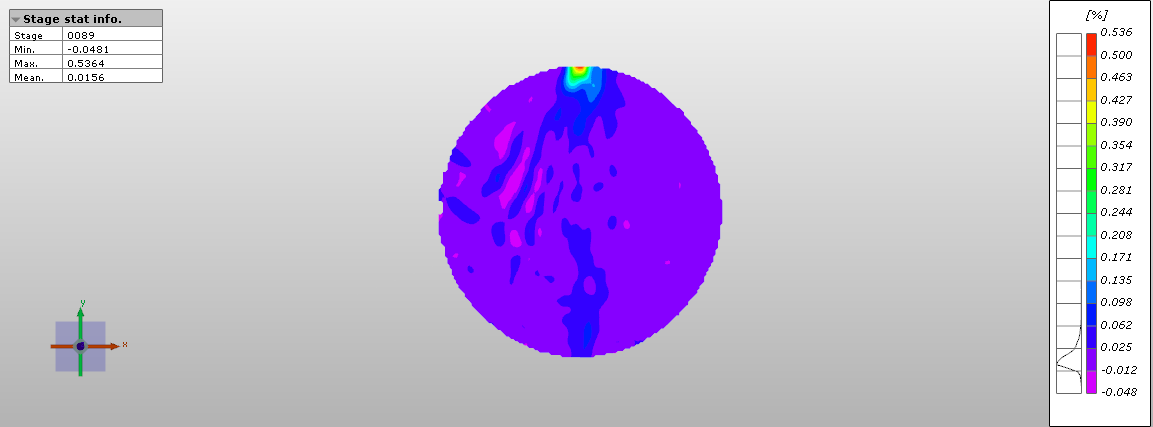

Supplement: S2 Data — (ZIP) [file pone.0294258.s002.zip › SNAPSERIES003/p0089.bmp]

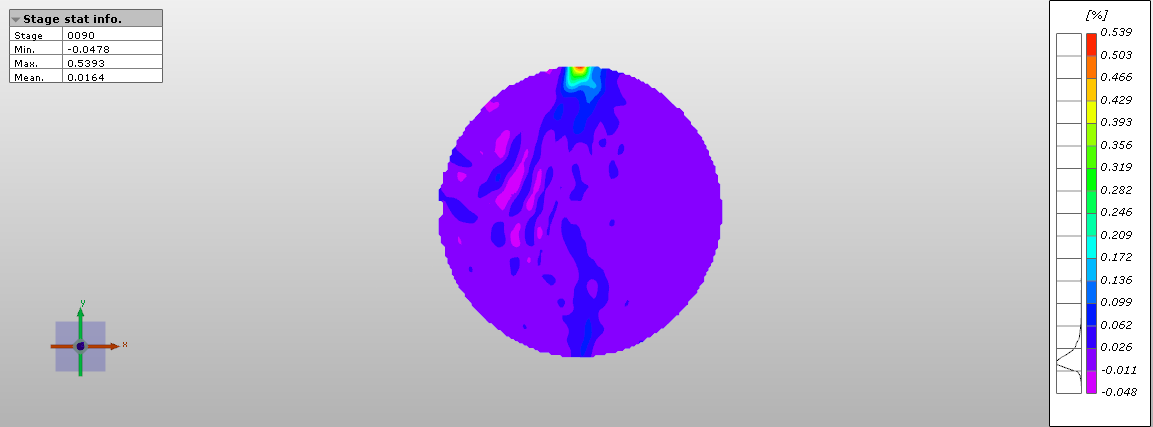

Supplement: S2 Data — (ZIP) [file pone.0294258.s002.zip › SNAPSERIES003/p0090.bmp]

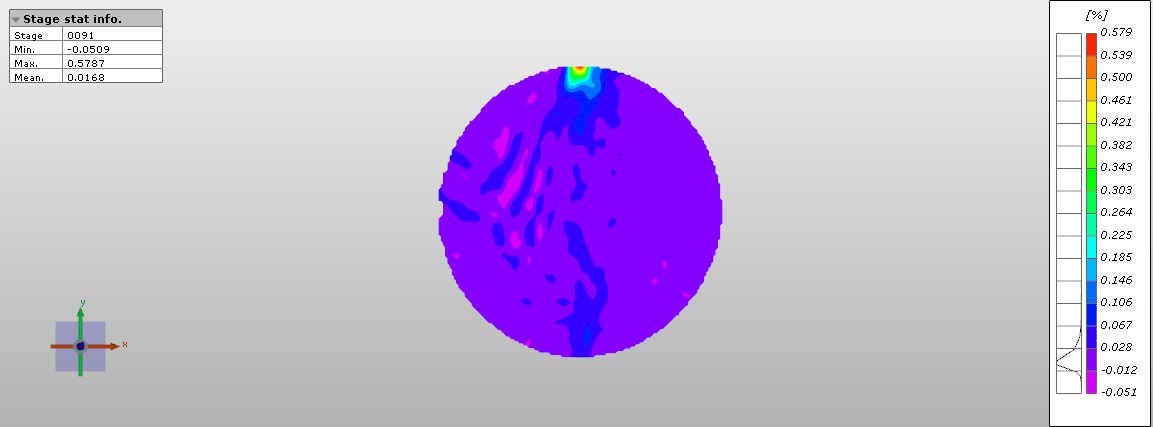

Supplement: S2 Data — (ZIP) [file pone.0294258.s002.zip › SNAPSERIES003/p0091.bmp]

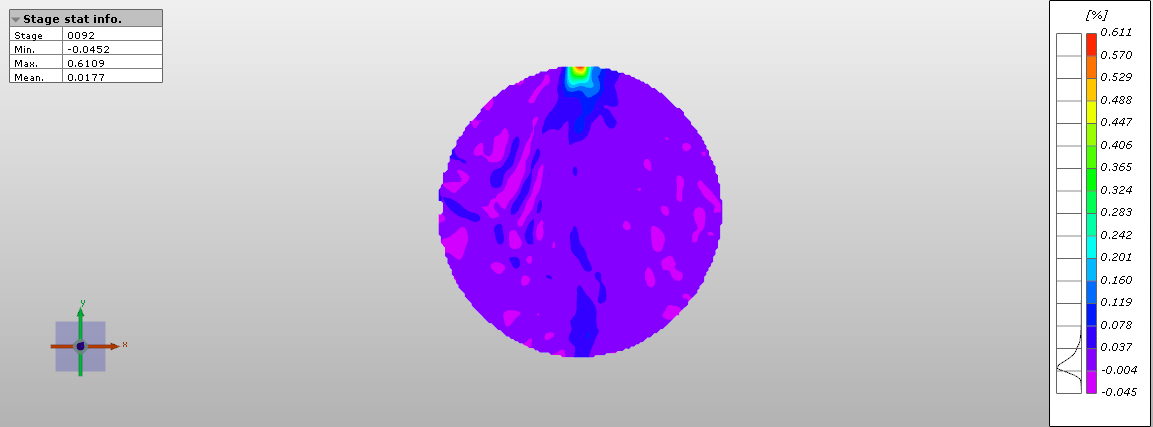

Supplement: S2 Data — (ZIP) [file pone.0294258.s002.zip › SNAPSERIES003/p0092.bmp]

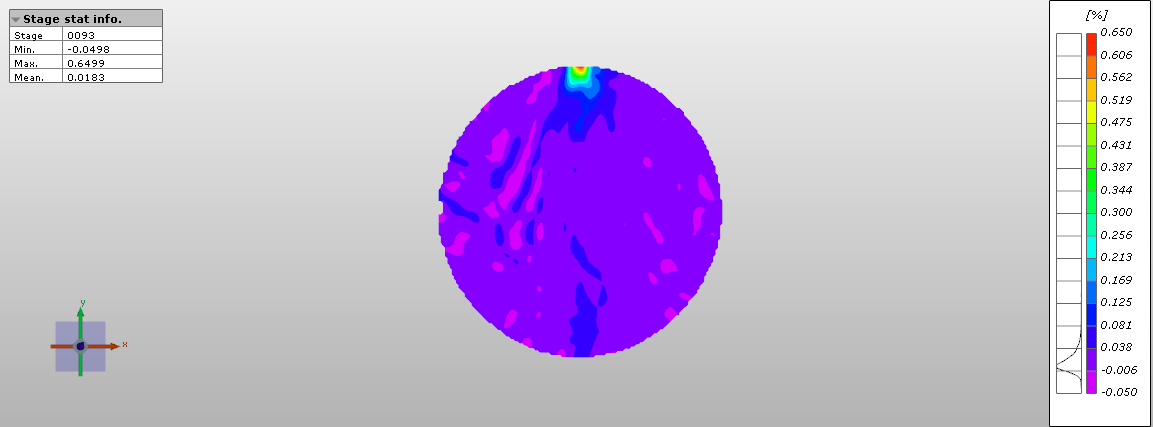

Supplement: S2 Data — (ZIP) [file pone.0294258.s002.zip › SNAPSERIES003/p0093.bmp]

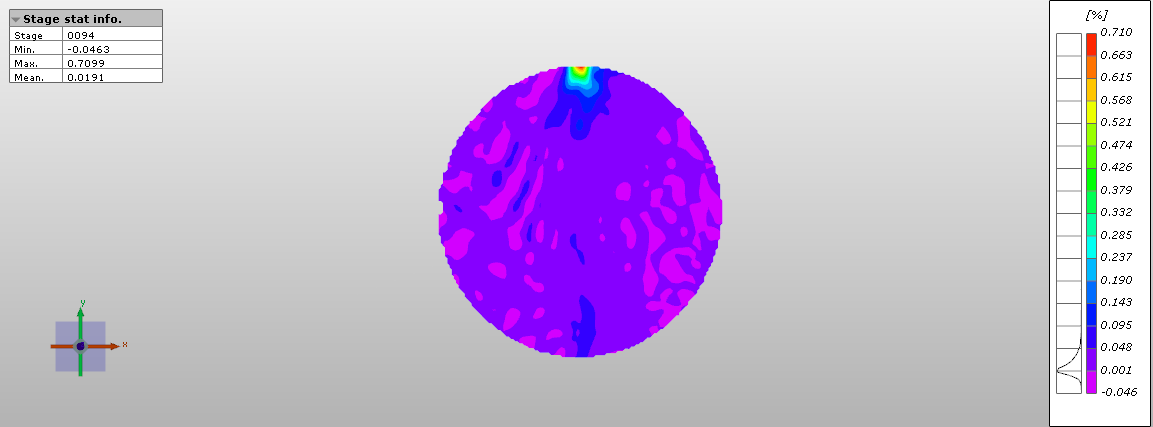

Supplement: S2 Data — (ZIP) [file pone.0294258.s002.zip › SNAPSERIES003/p0094.bmp]

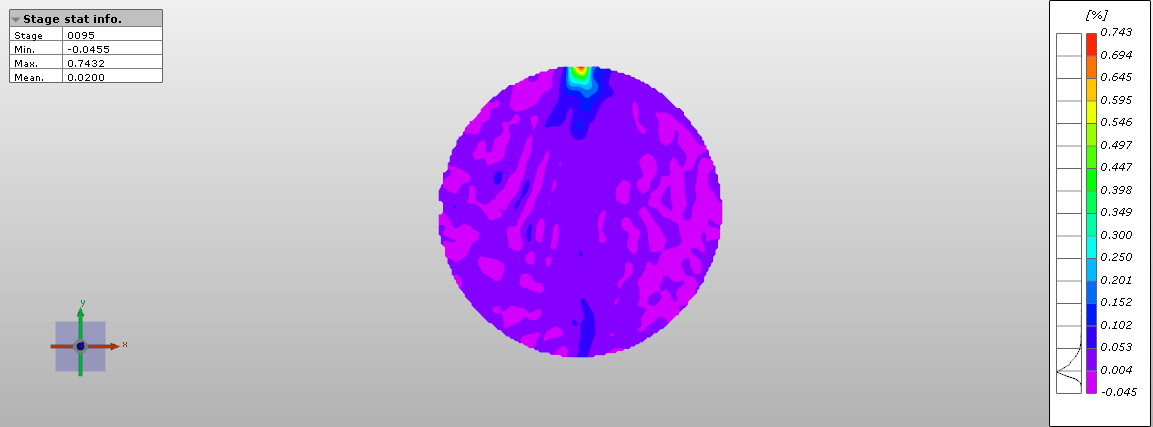

Supplement: S2 Data — (ZIP) [file pone.0294258.s002.zip › SNAPSERIES003/p0095.bmp]

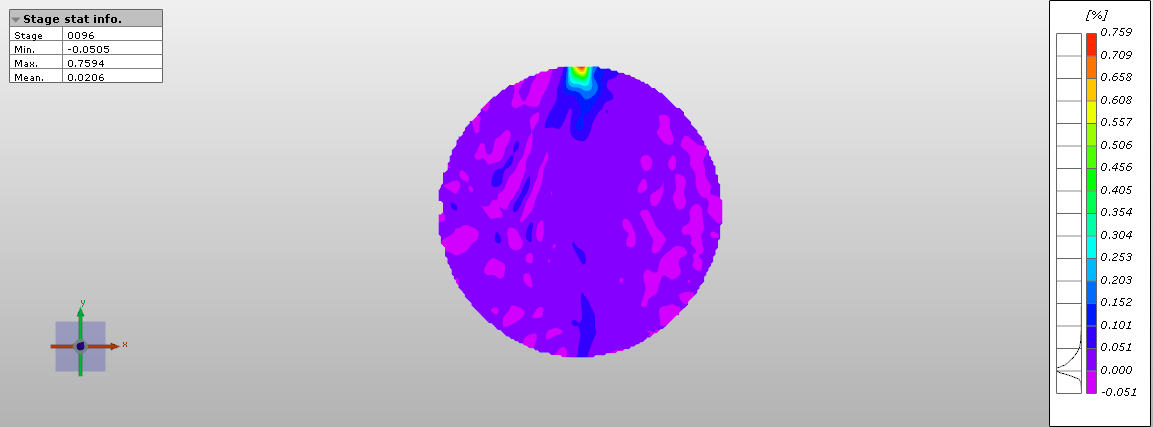

Supplement: S2 Data — (ZIP) [file pone.0294258.s002.zip › SNAPSERIES003/p0096.bmp]

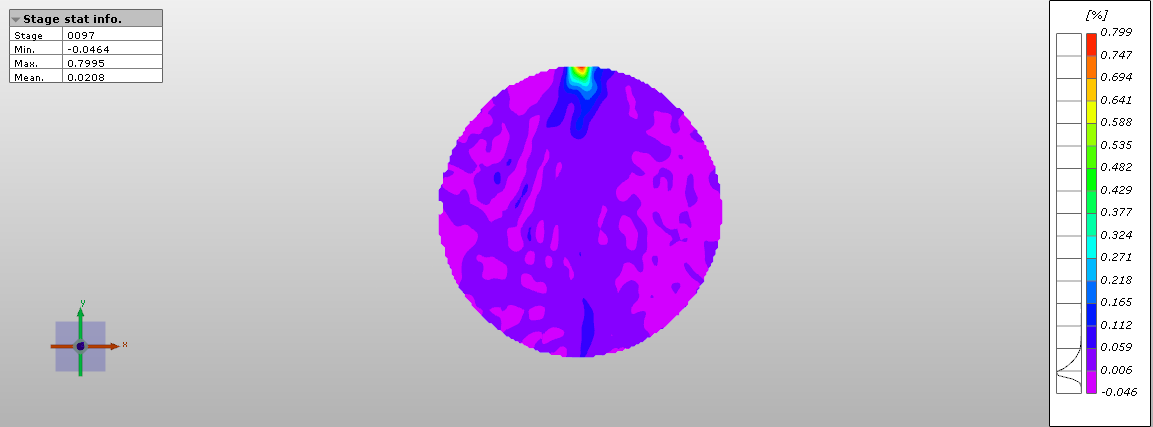

Supplement: S2 Data — (ZIP) [file pone.0294258.s002.zip › SNAPSERIES003/p0097.bmp]

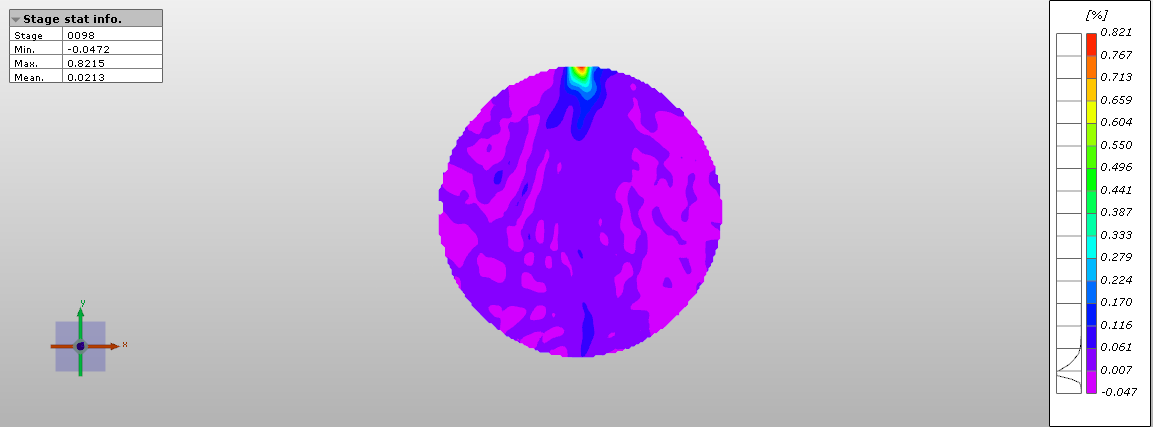

Supplement: S2 Data — (ZIP) [file pone.0294258.s002.zip › SNAPSERIES003/p0098.bmp]

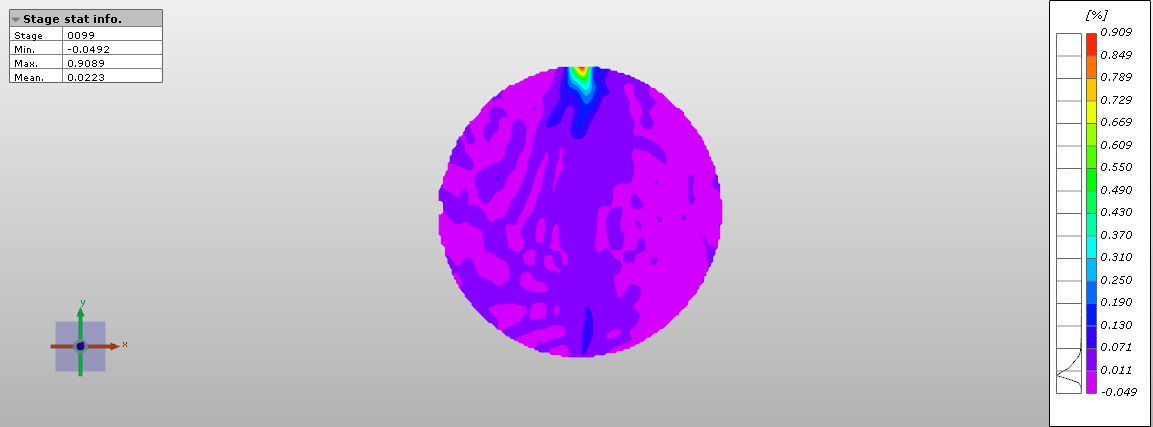

Supplement: S2 Data — (ZIP) [file pone.0294258.s002.zip › SNAPSERIES003/p0099.bmp]
